# Oxa-Michael-initiated cascade reactions of levoglucosenone

**DOI:** 10.3762/bjoc.18.151

**Published:** 2022-10-13

**Authors:** Julian Klepp, Thomas Bousfield, Hugh Cummins, Sarah V A-M Legendre, Jason E Camp, Ben W Greatrex

**Affiliations:** 1 School of Rural Medicine, University of New England, Armidale, NSW, 2351, Australia; 2 Department of Chemical Sciences, School of Applied Sciences, University of Huddersfield, Queensgate, Huddersfield, United Kingdom

**Keywords:** cascade reactions, green chemistry, levoglucosenone, oxa-Michael reaction

## Abstract

The reactions of aromatic aldehydes and levoglucosenone promoted by methoxide gives bridged α,β-unsaturated ketones, formed by a series of oxa-Michael-initiated cascade reactions in yields of up to 91% (14 examples). A complex series of equilibria operate during the reaction, and the formation of the bridged species is thermodynamically favored, except in the case of 5-methylfurfural and pyrrole-2-carboxaldehyde. This is the first report detailing this type of aldol/Michael cascade involving oxa-Michael initiation.

## Introduction

(−)-Levoglucosenone (**1**) is formed from the acid-catalyzed pyrolysis of cellulose along with minor amounts of furfural and 5-methylfurfural [1–3]. It has emerged as a promising starting material for enantioselective synthesis from materials derived from biomass pyrolysis, due to its reactive functionality, and the chirality which derives from glucose [4–7]. Reactions of **1** where the α,β-unsaturated ketone participates as an electrophile are usually completely diastereoselective, as the approach of the nucleophile is controlled by the oxymethylene bridge [8–10]. Many selective nucleophilic additions are known, and the reaction has been applied to the synthesis of disaccharides [11], the pheromone eldanolide [12], and flavoring compounds such as whiskey lactone [13]. The use of heteronucleophiles can also be used to initiate cascade processes in **1**, such as the Baylis–Hillman reaction [14], and the Rauhut–Currier reaction which gives dimers such as **2** as well as higher oligomers [10,15].

**Figure 1:**
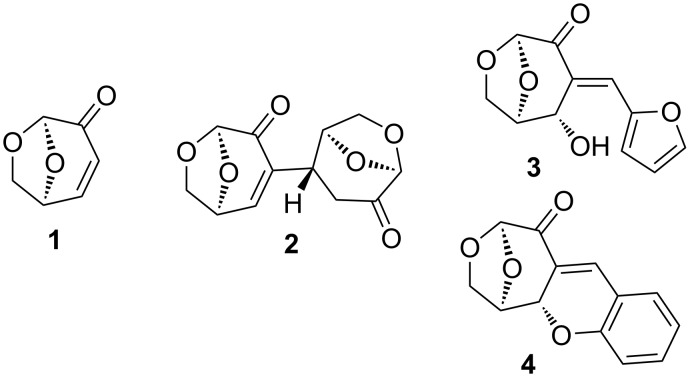
Levoglucosenone (**1**), known dimerization product **2**, and adducts **3** and **4**.

An oxa-Michael-initiated three-component intermolecular reaction of **1** with furfural and water has been reported to result in enone **3** (Figure 1) [16]. The reaction is interesting as both furfural and **1** are present along with water in crude biomass pyrolysates, and so the reaction could affect yields of **1** [3,17–18]. Samet and co-workers have reported a similar condensation of **1** with salicylaldehyde resulting in chiral chromene derivative **4** [19–20]. These types of oxa-Michael initiated aldol condensations were also of interest to us due to the previous work conducted on aldol adducts of **1** [14,21], and the potential to generate bio-derived chiral materials with reactive functional groups. We envisaged that the development of a larger library of adducts similar to **3** would be possible, and so have investigated the reaction of **1** with aromatic aldehydes in the presence of base in alcohol. It was found that the reaction outcome was dependent on the type of aldehyde used, and the reactions gave unanticipated products. We now disclose a hitherto unreported mode of reaction between α,β-unsaturated ketones and aromatic aldehydes promoted by base.

## Results and Discussion

The reactions of **1** with aromatic aldehydes and sodium methoxide in methanol were investigated at ambient and elevated temperatures (Table 1). In the reaction of benzaldehyde and **1** at 60° C, the sole product from the reaction contained resonances in the ^1^H NMR spectrum which supported a structure derived from a 2:1 ratio of **1**/benzaldehyde, and no structures analogous to **3** were obtained. The ^1^H NMR spectrum of the product had two non-equivalent vinylic protons β to the carbonyls at δ 6.74 and 6.61 ppm, and the H-3 resonances present in the starting material were absent. The 4-oxo-6,8-dioxabicyclo[3.2.1]octane ring-system has characteristic resonances, with a singlet for H5 around δ 5.40 ppm, the H1 signal at δ 4.50–5.00 ppm, and only H7α in the methylene coupled to H1 due to an approximate 90° dihedral angle for H7β. The doubling of these characteristic signals indicated that two of these bicyclic ring-systems were present and intact. HRMS analysis gave a sodiated ion at *m*/*z* 363.0837 indicating a formula of C_19_H_16_O_6_ which is consistent with structure **5a**. The two bicyclic ring-systems in **5a** are diastereotopic and therefore not chemically equivalent, and this was evident in the NMR spectra.

**Table 1:** Reactions of enone **1** and aldehydes promoted by NaOMe in MeOH.

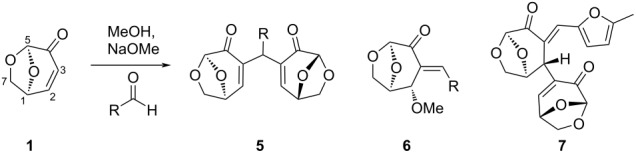			

Entry^a^	Aldehyde	Equiv^b^	Product (yield)^c^

1	PhCHO	1	**5a** (49)
2	PhCHO	0.5	**5a** (91)
3	PhCHO	2	**5a** (trace)
4^d^	PhCHO	0.5	**5a** (0)
5	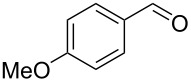	0.5	**5b** (67)
6	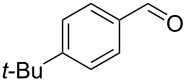	0.5	**5c** (65)
7	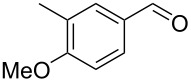	0.5	**5d** (89)
8	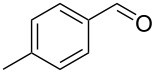	0.5	**5e** (79)
9	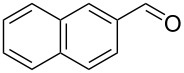	0.5	**5f** (83)
10	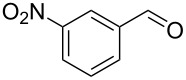	0.5	**5g** (84)
11	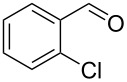	0.5	**5h** (83)
12	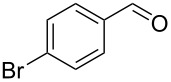	0.5	**5i** (83)
13	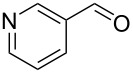	0.5	**5j** (81)
14	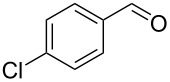	0.5	**5k** (77)
15	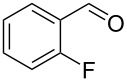	0.5	**5l** (87)
16	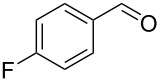	0.5	**5m** (82)
17	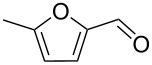	0.5	**6n** (8) **7** (4)
18	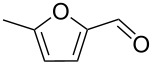	0.75	**6n** (30)

^a^Reactions were performed with 500 mg of LGO in 5 mL of 1.0 M NaOMe solution and heated to 60 °C for 24 hours; ^b^equivalents of aldehyde; ^c^isolated yield; ^d^1.0 M NaOH in EtOH.

The temperature played a significant role in the formation of coupled product **5a**. At ambient temperature and in the initial stages of the reaction at elevated temperature, a complex mixture resulted which consisted of the methanol addition product and many other species (NMR). When this complex mixture was heated to 60 °C, the mixture resolved and the major product was the bridged species. The equivalents of benzaldehyde and **1** were not critical in the reaction as both 2:1 and 1:1 ratios of **1**/benzaldehyde led to the bridged species **5a** as the major product (Table 1, entries 1 and 2), however, excess aldehyde slowed the formation of **5a** (Table 1, entry 3). The use of hydroxide in ethanol failed to yield any **5a** indicating the sensitivity of the reaction to conditions (Table 1, entry 4). The most effective procedure was to combine the reagents in a 2:1 ratio of **1**/aldehyde in a 1.0 M solution of NaOMe in MeOH and then heat the mixture (Table 1, entries 2, and 5–17).

**Scheme 1:**
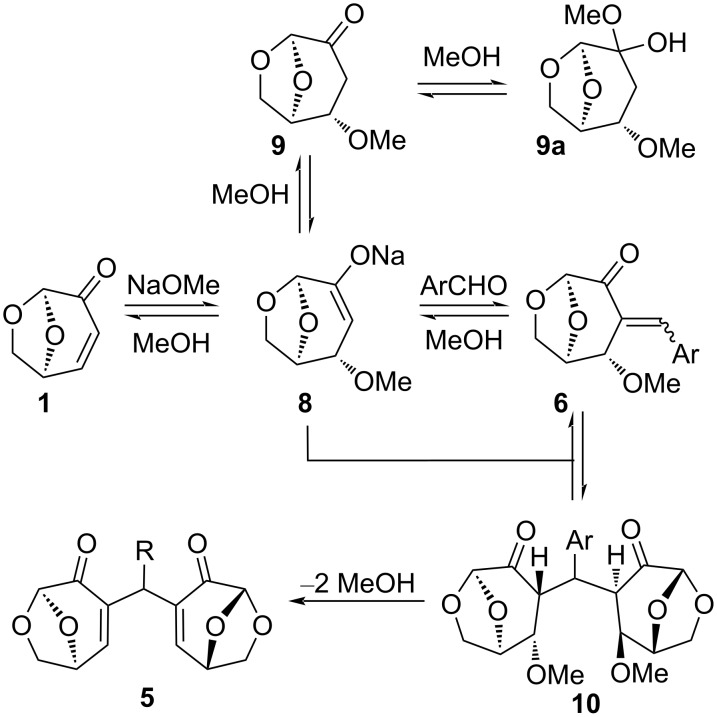
Proposed pathway for the formation of **5**.

Once conditions for the synthesis of **5a** had been identified, the scope of the reaction was explored with other aromatic aldehydes (Table 1). Purification of the products was straightforward as in many cases the bridged species could be isolated by precipitation with water and then recrystallization. The reaction of mildly electron-rich anisaldehyde and 4-methoxy-3-methylbenzaldehyde resulted in the bridged products **5b** and **5d** in good yields (Table 1, entries 5 and 7). Electron-poor aromatic aldehydes including 3-nitrobenzaldehyde and 3-pyridine carboxaldehyde also afforded good yields of the expected products **5g** and **5j** (Table 1, entries 10 and 13). The reaction of 5-methylfurfural afforded a low yield of **7**, and the aldol condensation product **6n** was also isolated (Table 1, entry 17), analogous to that reported for the reaction of **1**, furfural and hydroxide in water [16]. The isolation of **6n** was attributed to a slow second conjugate addition of the enolate (the reaction of **6** and **8**, Scheme 1), while **7** was formed via an endocyclic elimination of methanol rather than exocyclic elimination of water, and regioisomeric Rauhut–Currier reaction. Compound **7** was found to be unstable after isolation, possibly due to intermolecular reactions of the electron-poor olefin and furan ring. When a slight excess of **1** was used, only **6n** was isolated albeit in only 30% yield (Table 1, entry 18). Similarly, the reactions of electron-rich piperonal and 3,4-dimethoxybenzaldehyde failed following multiple attempts, which indicated the importance of the electronics of the aldehyde. The failure was probably in the latter parts of the sequence as aldol adducts from these aldehydes and the reduced ketone **12** are known [14]. The reactions of cinnamaldehyde, propanal, and pyrrole carboxaldehyde with **1** also failed to yield bridged species, and the complex mixtures that resulted from these reactions were not further examined.

**Figure 2:**
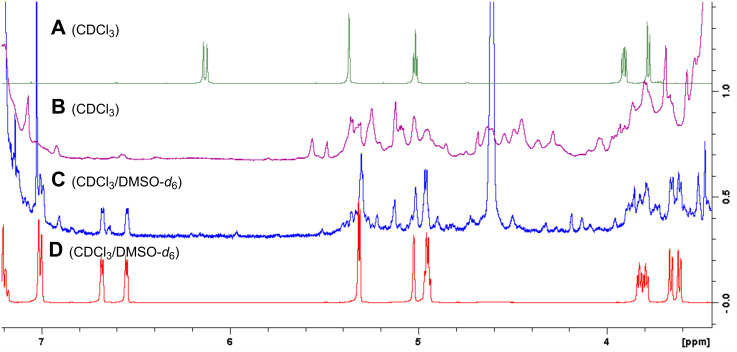
^1^H NMR spectra (500 MHz) of **1** (A), 1:1 **1**/PhCHO reaction mixture at 1 h at 60° C (B), mixture after 24 h at 60 °C (C), and product **5a** (D). DMSO-*d*_6_ was used in CDCl_3_ to dissolve precipitated product.

Mechanistically, the reaction is presumed to start with an oxa-Michael initiated aldol reaction promoted by a methoxide nucleophile giving enone **6** via enolate **8** (Scheme 1). A Rauhut–Currier-type reaction of **6** with the addition of another equivalent of **8**, followed by a subsequent double β-elimination leads to the observed product **5**. When the reaction was followed by NMR by sampling the mixture, consumption of **1** was rapid and intermediate mixtures were complex, suggesting that a series of equilibria are in operation (Figure 2). To gain further insight, CD_3_ONa was added to a mixture of **1** and benzaldehyde in CD_3_OD. The consumption of **1**, as measured by the disappearance of olefinic signals, was immediate upon addition of base and a compound assigned as **9a** appeared. The major intermediate was assigned as the hemiacetal **9a** rather than ketone **9**, due to the 0.31 ppm upfield shift for H5 (δ 4.83 ppm, CD_3_OD) relative to known ketone **9** (δ 5.14 ppm, CDCl_3_) [22]. Other hemiketal type structures may be responsible for some of the complexity as the reaction progresses, as this addition is common to the 6,8-dioxabicyclo[3.2.1]octan-4-one ring system. It is interesting that even with equimolar benzaldehyde and **1**, the 2:1 bridged species **5a** and not the 1:1 product **6a** was the major product (Table 1, entry 1 and Figure 2), presumably due to the equilibria and thermodynamics favoring adduct **5**. The Hantzsch dihydropyridine synthesis, and aldol/Michael sequences such as the reaction of hydroxytropolones with aromatic aldehydes give similar bridged α,β-unsaturated ketones; however, these reactions do not involve initiation by an oxa-Michael addition [23–24].

**Scheme 2:**
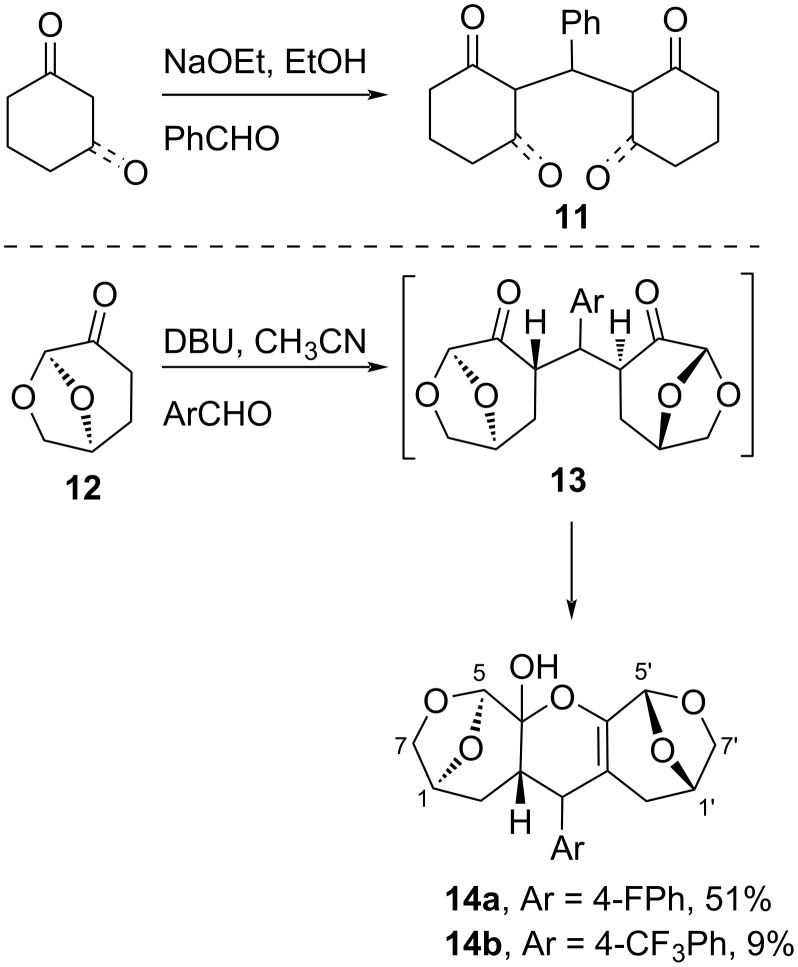
Known reactions giving **11**, and reactions of dihydrolevoglucosenone **12** and aromatic aldehydes with DBU.

The reaction between **1** and aromatic ketones under basic conditions is analogous to the well-known aldol/Michael cascade reaction observed between aldehydes and enolates giving di- and tetraketones **11** promoted by base (Scheme 2) [25]. The equivalent reaction has not been reported for dihydrolevoglucosenone (Cyrene™) **12**, and it was thought that the chiral 1,5-diketone products could be used to construct catalysts or ligands. The aldol/Michael cascade using conditions for the aldol reaction from **12** gave moderate to low yields of bridged species, most effectively with electron-poor aromatic aldehydes, and reactions had to be monitored to optimize conversions and prevent decomposition (Scheme 2) [14]. Analysis of the NMR spectra indicated that instead of the open chain diketones **13**, the products existed as the pentacyclic hemiacetals **14**.

The ^13^C NMR spectrum for **14a** had resonances at δ 144.5 and 104.4 ppm, consistent with an enol ether. In the 2D HMBC spectrum, crosspeaks between these double bond carbons were seen with the H2′-methylene and the H5′-acetal resonances. The characteristic ring-system resonances for the 6,8-dioxabicyclo[3.2.1]octane ring-systems were still present in the ^1^H NMR spectrum, with the H5 and H5′ acetal signals appearing as singlets at δ 5.39 and 5.30 ppm, and the H1/H1′ resonances each coupling to only one of the neighboring H7/H7′ methylene protons due to an approximate 90° dihedral angle.

## Conclusion

This work has described a novel reaction of a cyclic biomass-derived α,β-unsaturated ketone with aromatic aldehydes. The ready availability of levoglucosenone in large quantities from biomass could make compounds such as **5** of interest for green chiral materials applications.

## Supporting Information

File 1 Experimental details for all compounds including ^1^H and ^13^C NMR spectra.

File 2 ^1^H and ^13^C FID data and other spectra for new compounds.
