# Supplementary material for: Oxa-Michael-initiated cascade reactions of levoglucosenone
Source: Beilstein J Org Chem. 2022 Oct 13;18:1457–62. doi: 10.3762/bjoc.18.151 (PMC9577383; doi:10.3762/bjoc.18.151)

BEILSTEIN JOURNAL OF ORGANIC CHEMISTRY

Supporting Information

for

Oxa-Michael-initiated cascade reactions of levoglucosenone

Julian Klepp, Thomas Bousfield, Hugh Cummins, Sarah V. A.-M. Legendre,
Jason E. Camp and Ben W. Greatrex

Beilstein J. Org. Chem. **2022,** *18,* 1457–1462. doi:10.3762/bjoc.18.151

Experimental details for all compounds including ^{1}H and ^{13}C NMR spectra

License and Terms: This is a supporting information file under the terms of the Creative Commons Attribution License (https://creativecommons.org/licenses/by/4.0). Please note that the reuse, redistribution and reproduction in particular requires that the author(s) and source are credited and that individual graphics may be subject to special legal provisions.
The license is subject to the *Beilstein Journal of Organic Chemistry* terms and conditions: (https://www.beilstein-journals.org/bjoc/terms)

Contents

Contents S2

General experimental S4

General procedure for preparation of 5. ..... S4

^{1}H NMR (500 MHz, $CDCl_3$) for **5a** S17

^{13}C NMR (125 MHz, $CDCl_3$) for **5a** S18

COSY NMR for **5a** with assignments S19

HSQC NMR for **5a** with assignments S20

HMBC NMR spectrum for **5a** S21

^{1}H NMR (500 MHz, $CDCl_3$) for **5b** S22

^{13}C NMR (125 MHz, $CDCl_3$) for **5b** S23

^{1}H NMR (500 MHz, $CDCl_3$) for **5c** S24

^{13}C NMR (125 MHz, $CDCl_3$) for **5c** S25

^{1}H NMR (500 MHz, $CDCl_3$) for **5d** S26

^{13}C NMR (125 MHz, $CDCl_3$) for **5d** S27

^{1}H NMR (500 MHz, $CDCl_3$) for **5e** S28

^{13}C NMR (125 MHz, $CDCl_3$) for **5e** S29

^{1}H NMR (500 MHz, $CDCl_3$) for **5f** S30

^{13}C NMR (125 MHz, $CDCl_3$) for **5f** S31

^{1}H NMR (500 MHz, $CDCl_3$) for **5g** S32

^{13}C NMR (125 MHz, $CDCl_3$) for **5g** S33

^{1}H NMR (500 MHz, $CDCl_3$) for **5h** S34

^{13}C NMR (125 MHz, $CDCl_3$) for **5h** S35

^{1}H NMR (500 MHz, $CDCl_3$) for **5i** S36

^{13}C NMR (125 MHz, $CDCl_3$) for **5i** S37

^{1}H NMR (500 MHz, $CDCl_3$) for **5j** S38

^{13}C NMR (125 MHz, $CDCl_3$) for **5j** S39

^{1}H NMR (500 MHz, $CDCl_3$) for **5k** S40

^{13}C NMR (125 MHz, $CDCl_3$) for **5k** S41

^{1}H NMR (500 MHz, $CDCl_3$) for **5l** S42

^{13}C NMR (125 MHz, $CDCl_3$) for **5l** S43

^{1}H NMR (500 MHz, $CDCl_3$) for **5m** S44

^{13}C NMR (125 MHz, $CDCl_3$) for **5m** S45

^{1}H NMR (500 MHz, $CDCl_3$) for **6** S46

^{13}C NMR (125 MHz, $CDCl_3$) for **6** S47

^{1}H NMR (500 MHz, $CDCl_3$) for **7** S48

^{13}C NMR (125 MHz, $CDCl_3$) for **7** S49

HMBC spectrum of **7** showing diagnostic crosspeak between the C7 methylene and C2 .... S51

^{1}H NMR (500 MHz, $CDCl_3$) for **14a** S52

^{13}C NMR (125 MHz, $CDCl_3$) for **14a** S53

^{1}H NMR (500 MHz, $CDCl_3$) for **14b** S54

^{13}C NMR (125 MHz, $CDCl_3$) for **14b** S55

General experimental

All reactions were carried out under N_2 unless indicated. Solvents were removed using a rotary evaporator with a bath temperature of 40 °C and pressure between 10 and 700 mbar. Solvents were distilled before use. (−)-Levoglucosenone (**1**) was prepared using published procedures and 6,8-dioxabicyclo[3.2.1]octan-4-one (Cyrene,™ **12**) was obtained from Sigma-Aldrich. All other reagents were commercially available and were used as purchased. Except as otherwise indicated, reactions were magnetically stirred and monitored by NMR-spectroscopy or thin layer chromatography (TLC) using silica plates (silica gel 60 F254). Visualization occurred by fluorescence quenching under UV light and/or by staining with permanganate solution. Flash-chromatography was performed on silica-gel 60, using a regulated moderate air pressure. ^{1}H NMR were recorded at 298 K on a 500 MHz *Bruker Avance III* spectrometer and the spectra were referenced to $CDCl_3$ (δ 7.26 ppm) or TMS (δ 0.00 ppm). ^{13}C NMR spectra were recorded with 126 MHz and the residual solvent ($CDCl_3$, δ 77.0 ppm) was used as reference. NMR were assigned using COSY, NOESY, HSQC and HMBC experiments and the multiplicity was defined as follows: *s* = singlet, *d* = doublet, *t* = triplet, *q* = quartet, *m* = multiplet or unresolved, *br* = broad signal. IR spectra were recorded on a *Perkin Elmer* spectrometer using an ATR cell and the signals are given in wavenumbers (cm^{-1}). Optical rotation was measured on a *Rudolph Research Analytical* Autopol 1 polarimeter operating at the sodium D line. Melting points were measured using open glass capillaries and are uncorrected. HRMS were recorded in positive ESI mode (source temperature 80 °C, desolvation temperature 150 °C, capillary 2.5 kV).

General procedure for preparation of 5. (−)-Levoglucosenone (500 mg, 1.0 equiv, 3.96 mmol) and the aldehyde (0.5 equiv, 1.98 mmol) were mixed and then a 1.0 M NaOMe solution in MeOH (5 mL) was added. The resulting solution was heated to 60 °C for 24 h, then allowed to cool and 1.0 M HCl (15 mL) was added and the precipitate collected. The solid was then

dissolved or suspended in CH_2Cl_2 (5 mL) and hexanes (25 mL) were added. The resulting precipitate was collected, washed with cold hexanes (3 × 5 mL) and then further purified as specified.

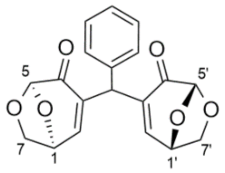

(1*S*,1'*S*,5*R*,5'*R*)-3,3'-(Phenylmethylene)bis(6,8-dioxabicyclo[3.2.1]oct-2-en-4-one) (5a). The reaction of **1** (500 mg, 3.96 mmol) with benzaldehyde (210 mg, 1.98 mmol) according to the general procedure, followed by recrystallization from $CHCl_3$ afforded **5a** (612 mg, 91%) as a colorless solid. mp 169-171 °C; $[\alpha]_D^{25}$ −239 (c 1.0 in CH_2Cl_2); ^{1}H NMR (500 MHz, $CDCl_3$): δ 7.34-7.29 (m, 2H, *m*-Ar*H*), 7.26-7.23 (m, 1H, *p*-Ar*H*), 7.09-7.05 (m, 2H, *o*-Ar*H*), 6.74 (dd, *J* = 5.0, 1.5 Hz, 1H, H2), 6.61 (dd, *J* = 5.0, 1.5 Hz, 1H, H2′), 5.38 (s, 1H, H5/5′), 5.37 (s, 1H, H5/5′), 5.09 (dd, *J* = 1.5, 1.5 Hz, 1H, C*H*Ar), 5.02 (dd, *J* = 4.9, 4.9 Hz, 1H, H1), 5.00 (dd, *J* = 4.9, 4.9 Hz, 1H, H1′), 3.89 (dd, *J* = 6.7, 4.9 Hz, 1H, H7α), 3.86 (dd, *J* = 6.7, 4.9 Hz, 1H, H7α′), 3.72 (d, *J* = 6.7 Hz, 1H, H7β), 3.68 (d, *J* = 6.7 Hz, 1H, H7β′); ^{13}C NMR (125 MHz, $CDCl_3$): δ 187.2 (C4/4′), 187.0 (C4/4′), 144.6 (C2′), 143.2 (C2), 138.5 (C3/3′), 138.2 (C3/3′), 137.5 (*i*-ArC), 128.8, 128.6, 127.3, 101.19 (C5/5′), 101.15 (C5/5′), 72.3 (C1/1′), 72.2 (C1/1′), 66.6 (C7/7′), 66.5 (C7/7′), 41.2 (*C*HPh); FT-IR (DCM) 3050, 2986, 1697, 1264, 1114, 897 cm$^{-1;}$ MS (ESI) *m/z* 363.1 [M + Na]$^+$; ESI-HRMS Calcd for [M + Na]$^+$; $C_{19}H_{16}O_6Na$: 363.0839; found: 363.0837.

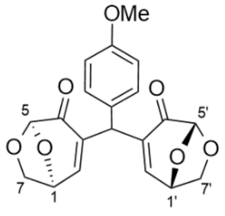

(1*S*,1'*S*,5*R*,5'*R*)-3,3'-((4-Methoxyphenyl)methylene)bis(6,8-dioxabicyclo[3.2.1]oct-2-en-4-one) (5b). The reaction of **1** (500 mg, 3.96 mmol) with *p*-anisaldehyde (270 mg, 1.98 mmol) according to the general procedure, and then crystallization from $CHCl_3$ afforded **5b** (489 mg, 67%)

as a colorless solid. mp 181-183 °C; $[\alpha]_D^{25}$ −335 (c 1.0 in CH_2Cl_2); ^1H NMR (500 MHz, $CDCl_3$): δ 7.00-6.96 (m, 2H, ArCH), 6.87-6.83 (m, 2H, ArCH), 6.72 (dd, *J* = 4.8, 1.3 Hz, 1H, H2/2′), 6.61 (dd, *J* = 4.8, 1.3 Hz, 1H, H2/2′), 5.37 (s, 1H, H5/5′), 5.36 (s, 1H, H5/5′), 5.03 (dd, *J* = 1.3, 1.3 Hz, 1H, C*H*Ar), 5.01 (ddd, *J* = 4.8, 4.7 Hz, 1H, H2/2′), 5.00 (dd, *J* = 4.8, 4.7 Hz, 1H, H2/2′), 3.88 (dd, *J* = 6.9, 4.7 Hz, 1H, H7α/7α′), 3.86 (dd, *J* = 6.9, 4.7 Hz, 1H, H7α/7α′), 3.78 (s, 3H, OC*H*3), 3.72 (d, *J* = 6.9 Hz, 1H, H7β/7β′), 3.67 (d, *J* = 6.9 Hz, 1H, H7β/7β′); ^{13}C NMR (125 MHz, $CDCl_3$): δ 187.3 (C4/4′), 187.1 (C4/4′), 158.8 (*C*OMe), 144.3 (C2/2′), 142.8 (C2/2′), 138.7 (C3/3′), 138.5 (C3/3′), 129.6 (Ar), 129.3 (Ar), 114.2 (Ar), 101.2 (C5/5′), 101.1 (C5/5′), 72.4 (C1/1′), 72.2 (C1/1′), 66.6 (C7/7′), 66.5 (C7/7′), 55.3 (O*C*H3), 40.4 (*C*HAr); FT-IR (neat) 3005, 2987, 1691, 1274, 1266, 1101 cm^{-1}; MS (ESI) *m/z* 393.1 $[M + Na]^+$; ESI-HRMS Calcd for $[M + Na]^+$; $C_{20}H_{18}O_7Na$: 393.0945; found: 393.0959.

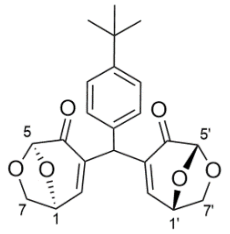

(1*S*,1'*S*,5*R*,5'*R*)-3,3'-((4-(*tert*-Butyl)phenyl)methylene)bis(6,8-dioxabicyclo[3.2.1]oct-2-en-4-one) (5c). The reaction of **1** (500 mg, 3.96 mmol) with 4-*tert*-butylbenzaldehyde (321 mg, 1.98 mmol) according to the general procedure, and then additional purification by column chromatography on silica (EtOAc:hexanes 1:1) and recrystallization with EtOAc:hexanes (1:1) afforded **5c** (510 mg, 65%) as a colorless solid. mp 168-170 °C; $[\alpha]_D^{25}$ −346 (c 1.0 in CH_2Cl_2); ^1H NMR (500 MHz, $CDCl_3$): δ 7.32-7.29 (m, 2H, ArH), 6.99-6.95 (m, 2H, ArH), 6.73 (dd, *J* = 5.0, 1.3 Hz, 1H, H2/2′), 6.62 (dd, *J* = 5.0, 1.3 Hz, 1H, H2/2′), 5.36 (s, 1H, H5/5′), 5.35 (s, 1H, H5/5′), 5.05 (br s, 1H, C*H*Ar), 5.00 (dd, *J* = 5.0, 5.0 Hz, 1H, H1/1′), 4.99 (dd, *J* = 5.0, 5.0 Hz, 1H, H1/1′), 3.86 (dd, *J* = 6.8, 5.0 Hz, 1H, H7α/7α′), 3.83 (dd, *J* = 6.8, 5.0 Hz, 1H, H7α/7α′), 3.70 (d, *J* = 6.8 Hz, 1H, H7β/7β′), 3.68 (d, *J* = 6.8 Hz, 1H, H7β/7β′), 1.28 (s, 9H, *t*-Bu); ^{13}C NMR (125 MHz, $CDCl_3$): δ 187.3 (C4/4′), 187.1 (C4/4′),

150.1, 144.4 (C2/2′), 143.1 (C2/2′), 138.5 (C3/3′), 138.3 (C3/3′), 134.1, 128.2, 125.7, 101.2 (C5/5′), 101.1 (C5/5′), 72.3 (C1/1′), 72.2 (C1/1′), 66.6 (C7/7′), 66.5 (C7/7′), 40.6 (*C*HAr), 34.4, 31.3; FT-IR (DCM) 2964, 1732, 1371, 1260, 982, 856 cm^{-1}; MS (ESI) *m/z* 397.1 $[M + H]^+$; ESI-HRMS Calcd for $[M + H]^+$; $C_{23}H_{25}O_6$: 397.1646; found: 397.1645.

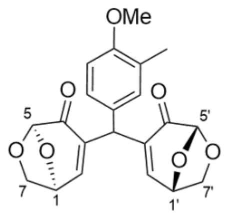

(1*S*,1'*S*,5*R*,5'*R*)-3,3'-((4-Methoxy-3-methylphenyl)methylene)bis(6,8-dioxabicyclo[3.2.1]oct-2-en-4-one) (5d). The reaction of **1** (500 mg, 3.96 mmol) with 3-methyl-*p*-anisaldehyde (297 mg, 1.98 mmol) according to the general procedure, and then washing with MeOH (3 × 5mL) afforded **5d** (677 mg, 89%) as a colorless solid. mp 165-167 °C; $[\alpha]_D^{25}$ −220 (c 1.0 in CH_2Cl_2); ^{1}H NMR (500 MHz, $CDCl_3$): δ 6.84-6.80 (m, 2H, ArH), 6.76-6.73 (m, 1H, ArH), 6.71 (dd, *J* = 5.0, 1.2 Hz, 1H, H2/2′), 6.61 (dd, *J* = 5.0, 1.4 Hz, 1H, H2/2′), 5.36 (s, 1H, H5/5′), 5.35 (s, 1H, H5/5′), 5.00 (dd, *J* = 5.0, 5.0 Hz, 1H, H1/1′), 5.00-4.98 (m, 2H, H1/1′/C*H*Ar), 3.88 (dd, *J* = 6.8, 5.0 Hz, 1H, H7α/7α′), 3.85 (dd, *J* = 6.8, 5.0 Hz, 1H, H7α/7α′), 3.79 (s, 3H, OCH_3), 3.71 (d, *J* = 6.8 Hz, 1H, H7β/7β′), 3.67 (d, *J* = 6.8 Hz, 1H, H7β/7β′), 2.17 (s, 3H, ArCH_3); ^{13}C NMR (125 MHz, $CDCl_3$): δ 187.3 (C4/4′), 187.2 (C4/4′), 156.9, 144.3 (C2/2′), 142.7 (C2/2′), 138.8 (C3/3′), 138.6 (C3/3′), 130.9, 128.7, 127.1, 126.7, 110.0, 101.2 (C5/5′), 101.1 (C5/5′), 72.4 (C1/1′), 72.2 (C1/1′), 66.6 (C7/7′), 66.5 (C7/7′), 55.3 (OMe), 40.4 (*C*HAr), 16.4 (*C*H_3Ar; FT-IR (neat) 2350, 1705, 1688, 1499, 1236, 980, 897 $cm^{-1;}$ MS (ESI) *m/z* 407.1 $[M + Na]^+$; ESI-HRMS Calcd for $[M + Na]^+$; $C_{21}H_{20}O_7Na$: 407.1101; found: 407.1106.

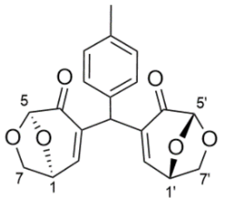

(1*S*,1'*S*,5*R*,5'*R*)-3,3'-(*p*-Tolylmethylene)bis(6,8-dioxabicyclo[3.2.1]oct-2-en-4-one) (5e). The reaction of **1** (500 mg, 3.96 mmol) with tolualdehyde (238 mg, 1.98 mmol) according to the general procedure afforded **5e** (554 mg, 79%) as a yellow solid. mp 175-177 °C; $[\alpha]_D^{25}$ −298 (c 1.0 in CH_2Cl_2); ^{1}H NMR (500 MHz, $CDCl_3$): δ 7.14-7.10 (m, 2H, ArH), 6.97-6.93 (m, 2H, ArH), 6.73 (dd, *J* = 4.8, 1.1 Hz, 1H, H2/2′), 6.61 (dd, *J* = 4.8, 1.1 Hz, 1H, H2/2′), 5.37 (s, 1H, H5/5′), 5.36 (s, 1H, H5/5′), 5.04 (br s, 1H, C*H*Ar), 5.00 (dd, *J* = 4.8, 4.8 Hz, 1H, H1/1′), 4.99 (dd, *J* = 4.8, 4.8 Hz, 1H, H1/1′), 3.88 (dd, *J* = 6.8, 4.6 Hz, 1H, H7α/7α′), 3.85 (dd, *J* = 6.8, 4.6 Hz, 1H, H7α/7α′), 3.71 (d, *J* = 6.8 Hz, 1H, H7β/7β′), 3.67 (d, *J* = 6.8 Hz, 1H, H7β/7β′), 2.31 (s, 3H, ArC*H*$_3$); ^{13}C NMR (125 MHz, $CDCl_3$): δ 187.3(C4/4′), 187.1 (C4/4′), 144.4 (C2/2′), 143.0 (C2/2′), 138.6 (C3/3′), 138.4 (C3/3′), 137.0, 134.3, 129.5, 128.5, 101.2 (C5/5′), 101.1 (C5/5′), 72.3 (C1/1′), 72.2 (C1/1′), 66.6 (C7/7′), 66.5 (C7/7′), 40.8 (*C*HAr), 21.0 (*C*H$_3$Ar); FT-IR (DCM) 3004, 2988, 1697, 1274, 1267, 912 cm^{-1}; MS (ESI) *m/z* 355.1 [M + Na]$^+$; ESI-HRMS Calcd for [M + H]$^+$; $C_{20}H_{19}O_6$: 355.1176; found: 355.1164.

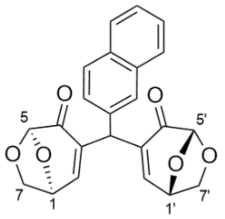

(1*S*,1'*S*,5*R*,5'*R*)-3,3'-(Naphthalen-2-ylmethylene)bis(6,8-dioxabicyclo[3.2.1]oct-2-en-4-one) (5f). The reaction of **1** (500 mg, 3.96 mmol) with 2-naphthaldehyde (309 mg, 1.98 mmol) according to the general procedure afforded **5f** (641 mg, 83%) as a colorless solid. mp 174-176 °C; $[\alpha]_D^{25}$ −389 (c 1.0 in CH_2Cl_2); ^{1}H NMR (500 MHz, $CDCl_3$): δ 7.84-7.76 (m, 3H, ArH), 7.52-7.44 (m, 3H, ArH), 7.23-7.19 (m, 1H, ArH), 6.86 (dd, *J* = 4.8, 1.2 Hz, 1H, H2/2′), 6.62 (dd, *J* = 4.8, 1.2 Hz, 1H, H2/2′), 5.40 (s, 1H, H5/5′), 5.39 (s, 1H, H5/5′), 5.26 (br s, 1H, C*H*Ar), 5.05 (dd, *J* = 4.8, 4.8 Hz, 2H, H1/1′), 5.00 (dd, *J* = 4.8, 4.8 Hz, 1H, H1/1′), 3.90 (dd, *J* = 6.7, 4.8 Hz, 1H, H7α/7α′), 3.86 (dd, *J* = 6.7, 4.8 Hz, 1H, H7α/7α′), 3.75 (d, *J* = 6.7 Hz, 1H, H7β/7β′), 3.68 (d, *J* = 6.7 Hz,

1H, H7β/7β′); ^{13}C NMR (125 MHz, $CDCl_3$): δ 187.2(C4/4′), 187.0 (C4/4′), 144.9 (C2/2′), 143.3 (C2/2′), 138.4 (C3/3′), 138.1 (C3/3′), 135.1, 133.3, 132.6, 128.7, 127.8, 127.6, 127.2, 126.9, 126.3, 126.1, 101.20 (C5/5′), 101.16 (C5/5′), 72.4 (C1/1′), 72.3 (C1/1′), 66.6 (C7/7′), 66.5 (C7/7′), 41.3 (*C*HAr); FT-IR (DCM) 3005, 2987, 1708, 1689, 1274, 1267, 903 cm^{-1}; MS (ESI) *m/z* $[M + Na]^+$ 413.1; ESI-HRMS Calcd for $[M + Na]^+$; $C_{23}H_{18}O_6Na$: 413.0996; found: 413.0967.

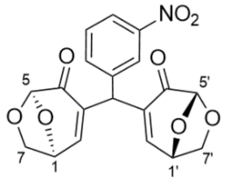

(1*S*,1'*S*,5*R*,5'*R*)-3,3'-((3-Nitrophenyl)methylene)bis(6,8-dioxabicyclo[3.2.1]oct-2-en-4-one) (5g). The reaction of **1** (500 mg, 3.96 mmol) with 3-nitrobenzaldehyde (295 mg, 1.98 mmol) according to the general procedure afforded **5g** (640 mg, 84%) as a brown solid. mp 173-175 °C; $[\alpha]_D^{25}$ −295 (c 1.0 in CH_2Cl_2); ^{1}H NMR (500 MHz, $CDCl_3$): δ 8.14 (ddd, *J* = 8.0, 2.3, 1.3 Hz, 1H, ArH), 7.93-7.90 (m, 1H, ArH), 7.52 (dd, *J* = 8.0, 8.0 Hz, 1H, ArH), 7.47 (ddd, *J* = 8.0, 1.3, 1.3 Hz, 1H, ArH), 6.78 (dd, *J* = 5.0, 1.3 Hz, 1H, H2/2′), 6.67 (dd, *J* = 5.0, 1.3 Hz, 1H, H2/2′), 5.40 (s, 1H, H5/5′), 5.39 (s, 1H, H5/5′), 5.19 (br s, 1H, C*H*Ar), 5.05 (dd, *J* = 5.0, 5.0 Hz, 1H, H1/1′), 5.05 (dd, *J* = 5.0, 5.0 Hz, 1H, H1/1′), 3.92 (dd, *J* = 6.9, 5.0 Hz, 1H, H7α/7α′), 3.89 (dd, *J* = 6.9, 5.0 Hz, 1H, H7α/7α′), 3.75 (d, *J* = 6.9 Hz, 1H, H7β/7β′), 3.72 (d, *J* = 6.9 Hz, 1H, H7β/7β′); ^{13}C NMR (125 MHz, $CDCl_3$): δ 186.8 (2C, C4/4′), 148.5, 144.9 (C2/2′), 144.2 (C2/2′), 139.9, 137.31 (C3/3′), 137.27 (C3/3′), 135.0, 129.9, 123.1, 122.6, 101.04 (C5/5′), 101.00 (C5/5′), 72.3 (C1/1′), 72.2 (C1/1′), 66.63 (C7/7′), 66.55 (C7/7′), 41.1 (*C*HAr); FT-IR (DCM) 2982, 1694, 1522, 1349, 1260, 1089, 897 $cm^{-1;}$ MS (ESI) *m/z* 408.1 $[M + Na]^+$; ESI-HRMS Calcd for $[M + Na]^+$; $C_{19}H_{15}O_8NNa$: 408.0690; found: 408.0700.

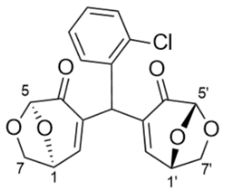

(1*S*,1'*S*,5*R*,5'*R*)-3,3'-((2-Chlorophenyl)methylene)bis(6,8-dioxabicyclo[3.2.1]oct-2-en-4-one) (5h). The reaction of **1** (500 mg, 3.96 mmol) with 2-chlorobenzaldehyde (278 mg, 1.98 mmol) according to the general procedure, followed by washing the precipitate with MeOH (3 × 5 mL) afforded **5h** (586 mg, 83%) as a yellow solid. mp 189-191 °C; $[\alpha]_D^{25}$ −384 (c 1.0 in CH_2Cl_2); ^{1}H NMR (500 MHz, $CDCl_3$): δ 7.42-7.37 (m, 1H, ArH), 7.24-7.17 (m, 2H, ArH), 6.94 (dd, *J* = 6.6, 2.0 Hz, 1H, ArH), 6.76 (dd, *J* = 4.8, 1.1 Hz, 1H, H2/2′), 6.51 (dd, *J* = 4.8, 1.1 Hz, 1H, H2/2′), 5.42 (br s, 1H, H5/5′), 5.40 (br s, 2H, H5/5′), 5.04 (dd, *J* = 4.8, 4.8 Hz, 1H, H1/1′), 4.99 (dd, *J* = 4.8, 4.8 Hz, 1H, H1/1′), 3.90, (dd, *J* = 6.7, 4.8 Hz, 1H, H7α/7α′), 3.88 (dd, *J* = 6.7, 4.8 Hz, 1H, H7α/7α′), 3.75-3.71 (m, 2H, H7β/7β′); ^{13}C NMR (125 MHz, $CDCl_3$): δ 186.9 (C4/4′), 186.8 (C4/4′), 144.6 (C2/2′), 144.1 (C2/2′), 137.4 (C3/3′), 137.1 (C3/3′), 136.0, 134.3, 130.3, 128.8, 128.7, 126.8, 101.18 (C5/5′), 101.17 (C5/5′), 72.3 (C1/1′), 72.2 (C1/1′), 66.6 (C7/7′), 66.5 (C7/7′), 38.6 (*C*HAr); FT-IR (DCM) 2962. 2868, 1702, 1267, 982 cm^{-1}; MS (ESI) *m/z* 397.1 $[M + Na]^+$; ESI-HRMS Calcd for $[M + H]^+$; $C_{19}H_{16}O_6Cl$: 375.0630; found: 375.0626.

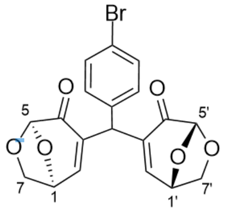

(1*S*,1'*S*,5*R*,5'*R*)-3,3'-((4-Bromophenyl)methylene)bis(6,8-dioxabicyclo[3.2.1]oct-2-en-4-one) (5i). The reaction of **1** (500 mg, 3.96 mmol) with 4-bromobenzaldehyde (366 mg, 1.98 mmol) according to the general procedure, followed by washing with MeOH (3 × 5 mL) afforded **5i** (688 mg, 83%) as a yellow solid. mp 183-185 °C; $[\alpha]_D^{25}$ −327 (c 1.0 in CH_2Cl_2); ^{1}H NMR (500 MHz, $CDCl_3$): δ 7.47-7.43 (m, 2H, ArH), 6.97-6.93 (m, 2H, ArH), 6.74 (dd, *J* = 4.8, 1.3 Hz, 1H, H2/2′), 6.61 (dd, *J* = 4.8, 1.3 Hz, 1H, H2/2′), 5.38 (s, 1H, H5/5′), 5.37 (s, 1H, H5/5′), 5.04 (br s, 1H, C*H*Ar), 5.03-5.00 (m, 2H, H1/1′), 3.89 (dd, *J* = 6.9, 4.8 Hz, 1H, H7α/7α′), 3.86 (dd, *J* = 6.9, 4.8 Hz, 1H, H7α/7α′), 3.72 (d, *J* = 6.9 Hz, 1H, H7β/7β′), 3.67 (d, *J* = 6.9 Hz, 1H, H7β/7β′); ^{13}C NMR

(125 MHz, $CDCl_3$): δ 187.0 (C4/4′), 186.9 (C4/4′), 144.7 (C2/2′), 143.4 (C2/2′), 138.0 (C3/3′), 137.8 (C3/3′), 136.7, 132.0, 130.3, 121.4, 101.13 (C5/5′), 101.09 (C5/5′), 72.3 (C1/1′), 72.2 (C1/1′), 66.6 (C7/7′), 66.5 (C7/7′), 40.8 (*C*HAr); FT-IR (DCM) 2970, 2242, 1697, 1114, 909, 897 cm^{-1}; MS (ESI) *m/z* 441.0 $[M + Na]^+$; ESI-HRMS Calcd for $[M + H]^+$; $C_{19}H_{16}O_6Br$: 419.0125; found: 419.0139.

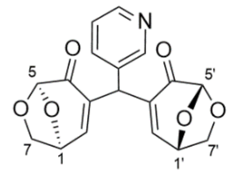

(1*S*,1'*S*,5*R*,5'*R*)-3,3'-(Pyridin-3-ylmethylene)bis(6,8-dioxabicyclo[3.2.1]oct-2-en-4-one) (5j). The reaction of **1** (500 mg, 3.96 mmol) with 3-pyridinecarboxaldehyde (212 mg, 1.98 mmol) according to the general procedure followed by crystallization from $CHCl_3$ afforded **5j** (545 mg, 81%) as a colorless solid. mp 189-191 °C; $[\alpha]_D^{25}$ −312 (c 1.0 in CH_2Cl_2); ^1H NMR (500 MHz, $CDCl_3$): δ 8.54-8.51 (m, 1H, ArH), 8.37 (br s, 1H, ArH), 7.45-7.38 (m, 1H, ArH), 7.29-7.25 (m, 1H, ArH), 6.78 (d, *J* = 5.0 Hz, 1H, H2/2′), 6.66 (d, *J* = 5.0 Hz, 1H, H2/2′), 5.40 (s, 1H, H5/5′), 5.39 (s, 1H, H5/5′), 5.10 (s, 1H, C*H*Ar), 5.03 (dd, *J* = 5.0, 5.0 Hz, 1H, H1/1′), 5.03 (dd, *J* = 5.0, 5.0 Hz, 1H, H1/1′), 3.90 (dd, *J* = 6.9, 5.0 Hz, 1H, H7α/7α′), 3.88 (dd, *J* = 6.9, 5.0 Hz, 1H, H7α/7α′), 3.73 (d, *J* = 6.9 Hz, 1H, H7β/7β′), 3.69 (d, *J* = 6.9 Hz, 1H, H7β/7β′); ^{13}C NMR (125 MHz, $CDCl_3$): δ 186.9 (C4/4′), 186.8 (C4/4′), 150.0, 148.8, 144.8 (C2/2′), 143.8 (C2/2′), 137.5 (C3/3′), 137.4 (C3/3′), 136.1, 133.3, 123.6, 101.1 (C5/5′), 101.0 (C5/5′), 72.3 (C1/1′), 72.2 (C1/1′), 66.6 (C7/7′), 66.5 (C7/7′), 39.2 (*C*HAr); FT-IR (DCM) 3054, 3004, 1709, 1422, 1273, 950 $cm^{-1;}$ MS (ESI) *m/z* 342.1 $[M + H]^+$; ESI-HRMS Calcd for $[M + H]^+$; $C_{18}H_{16}O_6N$: 342.0972; found: 342.0965.

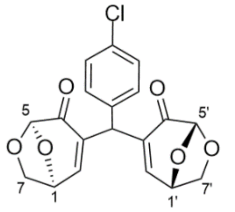

(1*S*,1'*S*,5*R*,5'*R*)-3,3'-((4-Chlorophenyl)methylene)bis(6,8-dioxabicyclo[3.2.1]oct-2-en-4-one) (5k). The reaction of **1** (500 mg, 3.96 mmol) with 4-chlorobenzaldehyde (278 mg, 1.98 mmol) according to the general procedure, with the product further washed with MeOH (3 × 5 mL) afforded **5k** (571 mg, 77%) as a yellow solid. mp 192-194 °C; $[\alpha]_D^{25}$ −335 (c 1.0 in CH_2Cl_2); ^{1}H NMR (500 MHz, $CDCl_3$): δ 7.31-7.27 (m, 2H, ArH), 7.03-6.98 (m, 2H, ArH), 6.73 (dd, *J* = 4.8, 1.4 Hz, 1H, H2/2′), 6.61 (dd, *J* = 4.8, 1.4 Hz, 1H, H2/2′), 5.38 (s, 1H, H5/5′), 5.37 (s, 1H, H5/5′), 5.05 (br s, 1H, C*H*Ar), 5.04-5.00 (m, 2H, H1/1′), 3.89 (dd, *J* = 6.6, 4.8 Hz, 1H, H7α/7α′), 3.86 (dd, *J* = 6.6, 4.8 Hz, 1H, H7α/7α′), 3.72 (d, *J* = 6.6 Hz, 1H, H7β/7β′), 3.67 (d, *J* = 6.6 Hz, 1H, H7β/7β′); ^{13}C NMR (125 MHz, $CDCl_3$): δ 187.1 (C4/4′), 186.9 (C4/4′), 144.7 (C2/2′), 143.4 (C2/2′), 138.1 (C3/3′), 137.9 (C3/3′), 136.1, 133.3, 129.9, 129.0, 101.13 (C5/5′), 101.09 (C5/5′), 72.3 (C1/1′), 72.2 (C1/1′), 66.6 (C7/7′), 66.5 (C7/7′), 40.8 (*C*HAr); FT-IR (DCM) 3004, 2987, 1710, 1460, 1267, 913 cm$^{-1;}$ MS (ESI) *m/z* 397.0 [M + Na]$^+$; ESI-HRMS Calcd for [M + Na]$^+$; $C_{19}H_{15}O_6ClNa$: 397.0449; found: 397.0457.

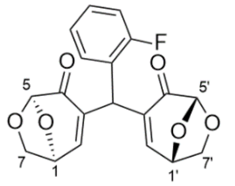

(1*S*,1'*S*,5*R*,5'*R*)-3,3'-((2-Fluorophenyl)methylene)bis(6,8-dioxabicyclo[3.2.1]oct-2-en-4-one) (5l). The reaction of **1** (500 mg, 3.96 mmol) with 2-fluorobenzaldehyde (245 mg, 1.98 mmol) according to the general procedure, followed by washing with MeOH (3 × 5 mL) afforded **5l** (617 mg, 87%) as a colorless solid. mp 161-163 °C; $[\alpha]_D^{25}$ −372 (c 1.0 in CH_2Cl_2); ^{1}H NMR (500 MHz, $CDCl_3$): δ 7.27-7.23 (m, 1H, ArH), 7.10 (m, 2H, ArH), 7.00-6.96 (m, 1H, ArH), 6.78 (dd, *J* = 4.9, 1.0 Hz, 1H, H2/2′), 6.65 (dd, *J* =4.9, 1.0 Hz, 1H, H2/2′), 5.39 (s, 1H, H5/5′), 5.38 (s, 1H, H5/5′), 5.30 (br s, 1H, C*H*Ar), 5.04-5.00 (m, 2H, H1/1′), 3.90 (dd, *J* = 6.8, 4.9 Hz, 1H, H7α/7α′), 3.87 (dd, *J* = 6.8, 4.9 Hz, 1H, H7α/7α′), 3.73 (d, *J* = 6.8 Hz, 1H, H7β/7β′), 3.71 (d, *J* = 6.8 Hz, 1H,

H7β/7β′); ^{13}C NMR (125 MHz, $CDCl_3$): δ 187.0 (C4/4′), 186.9 (C4/4′), 160.4 (*J* = 247 Hz), 144.2 (C2/2′), 143.7 (C2/2′), 137.0 (C3/3′), 136.9 (C3/3′), 129.6 (d, *J* = 3.7 Hz), 129.2 (d, *J* = 8.3 Hz), 125.1 (d, *J* = 14.0 Hz), 124.3 (d, *J* = 3.5 Hz), 116.2 (d, *J* = 21.8 Hz), 101.17 (C5/5′), 101.14 (C5/5′), 72.3 (C1/1′), 72.2 (C1/1′), 66.6 (C7/7′), 66.5 (C7/7′), 35.4 (d, *J* = 3.0 Hz, *C*HAr); FT-IR (neat) 2342, 1697, 1488, 1224, 1115, 974, 899 $cm^{-1;}$ ESI-HRMS Calcd for [M + Na]$^+$; $C_{19}H_{15}O_6FNa$: 381.0745; found: 381.0736.

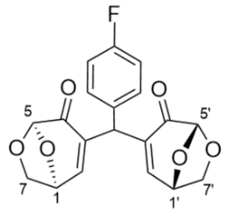

(1*S*,1'*S*,5*R*,5'*R*)-3,3'-((4-Fluorophenyl)methylene)bis(6,8-dioxabicyclo[3.2.1]oct-2-en-4-one) (5m). The reaction of **1** (500 mg, 3.96 mmol) with 4-fluorobenzaldehyde (245 mg, 1.98 mmol) according to the general procedure, followed by washing with MeOH (3 × 5 mL) afforded **5m** (581 mg, 82%) as a colorless solid. mp 157-159 °C; $[\alpha]_D^{25}$ −368 (c 1.0 in CH_2Cl_2); ^{1}H NMR (500 MHz, $CDCl_3$): δ 7.06-6.98 (m, 4H, ArH), 6.73 (dd, *J* = 4.9, 1.4 Hz, 1H, H2/2′), 6.60 (dd, *J* = 4.9, 1.4 Hz, 1H, H2/2′), 5.38 (s, 1H, H5/5′), 5.37 (s, 1H, H5/5′), 5.06 (br s, 1H, C*H*Ar), 5.02 (dd, *J* = 4.9, 4.9 Hz, 1H, H1/1′), 5.01 (dd, *J* = 4.9, 4.9 Hz, 1H, H1/1′), 3.89 (dd, *J* = 6.8, 4.9 Hz, 1H, H7α/7α′), 3.86 (dd, *J* = 6.8, 4.9 Hz, 1H, H7α/7α′), 3.71 (d, *J* = 6.8 Hz, 1H, H7β/7β′), 3.67 (d, *J* = 6.8 Hz, 1H, H7β/7β′); ^{13}C NMR (125 MHz, $CDCl_3$): δ 187.1 (C4/4′), 187.0 (C4/4′), 162.0 (d, *J* = 245 Hz), 144.6 (C2/2′), 143.2 (C2/2′), 138.3 (C3/3′), 138.2 (C3/3′), 133.2 (d, *J* = 3.6 Hz), 130.1 (d, *J* = 8.0 Hz), 115.8 (d, *J* = 20.0 Hz), 101.14 (C5/5′), 101.09 (C5/5′), 72.3 (C1/1′), 72.2 (C1/1′), 66.6 (C7/7′), 66.5 (C7/7′), 40.6 (*C*HAr); FT-IR (neat) 2350, 1696, 1506, 1115, 981, 814 $cm^{-1;}$ MS (ESI) *m/z* 381.1 [M + Na]$^+$; ESI-HRMS Calcd for [M + Na]$^+$; $C_{19}H_{15}O_6FNa$: 381.0745; found: 381.0725.

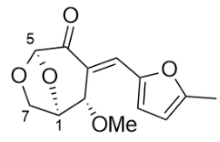

(1*R*,2*S*,3*E*,5*R*)-2-Methoxy-3-((5-methylfuran-2-yl)methylidene)-6,8-dioxabicyclo[3.2.1]octan-4-one (6n). ^{1}H NMR (500 MHz, $CDCl_3$): δ 7.55 (s, 1H, C=C*H*), 6.80 (d, *J* = 3.4 Hz, 1H, ArH), 6.22-6.20 (m, 1H, ArH), 5.32 (s, 1H, H5), 5.00 (ddd, *J* = 6.1, 1.7, 1.3 Hz, 1H, H1), 4.61 (br s, 1H, H2), 3.97 (dd, *J* = 7.6, 6.1, 1.7 Hz, 1H, H7α), 3.74 (dd, *J* = 7.6, 1.7 Hz, 1H, H7β), 3.56 (s, 3H, OCH_3), 2.41 (s, 3H, ArCH_3); ^{13}C NMR (125 MHz, $CDCl_3$): δ 188.3 (C4), 157.8, 149.5, 130.5 (=*C*H), 122.6, 122.1, 109.8, 101.0 (C5), 77.1 (C2), 73.7 (C1), 64.5 (C7), 56.2 (OMe), 14.1 (Me); FT-IR (neat) 2972, 2901, 1694, 1601, 1560, 990, 903 cm^{-1}; MS (ESI): *m/z* 273 (M + Na^{+}).

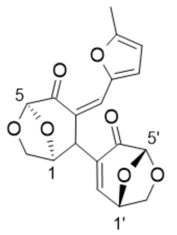

2-((1′*S*,5′*R*)-4-Oxo-6,8-dioxabicyclo[3.2.1]oct-2-en-3-yl)-3-((5-methylfuran-2-yl)methylidene)-(1*S*,5*R*)-6,8-dioxabicyclo[3.2.1]octan-4-one (7). A mixture of **1** (500 mg, 3.96 mmol) and 5-methylfurfural (218 mg, 1.98 mmol) in 1 M NaOMe in MeOH (5 mL) was stirred at 25 °C for 72 hours. The mixture was diluted with water (30 mL) and then extracted with CH_2Cl_2 (3 × 20 mL), the organic extracts were dried, concentrated under reduced pressure and the residue purified by flash chromatography (2:1 hexanes/EtOAc) to give **7** (43 mg, 4%) as a pale-yellow gum that decomposed upon standing, and **6n** (42 mg, 8%) as a yellow oil. **7**: ^{1}H NMR (500 MHz, $CDCl_3$): δ 7.53 (br d, *J* = 1.4 Hz, 1H, =C*H*Ar), 6.77 (dd, *J* = 4.6 Hz, 1H, H2′), 6.61 (br d, *J* = 3.4 Hz, 1H, ArH), 6.09-6.07 (m, 1H, ArH), 5.47 (s, 1H, H5′), 5.30 (s, 1H, H5), 4.96 (dd, *J* = 4.6, 4.6 Hz, 1H, H1′), 4.55 (br d, *J* = 5.8 Hz, 1H, H1), 4.40 (br s, 1H, H2), 4.01 (dd, *J* = 7.6, 5.8 Hz, 1H, H7α), 3.95 (dd, *J* = 7.6, 1.4 Hz, 1H, H7β), 3.74 (dd, *J* = 6.6, 4.6 Hz, 1H, H7′α), 3.10 (d, *J* = 6.6 Hz, 1H, H7′β), 2.24 (s, 3H, ArCH_3); ^{13}C NMR (125 MHz, $CDCl_3$): δ 188.8, 188.3, 158.6, 149.4, 143.0, 138.0, 127.8, 122.3, 121.7, 109.3, 101.3, 101.0, 75.1, 72.4, 68.4, 66.1, 42.1, 13.7. **7** decomposed upon standing to give an insoluble yellow/brown gum.

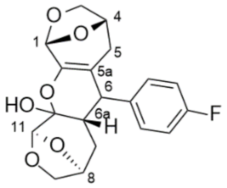

(1*R*,4*S*,6a*S*,8*S*,11*R*)-6-(4-Fluorophenyl)-3,4,5,6,6a,7,8,9,11,11a-decahydro-1*H*-1,4:8,11-diepoxypyrano[2,3-*c*:6,5-*c*']bis(oxepine)-11a-ol (14a). To a stirred solution of Cyrene™ (**12**, 820 µL, 1.02 g, 8.0 mmol) in acetonitrile (2 mL) was added 4-fluorobenzaldehyde (430 µL, 4.0 mmol) and DBU (987 µL, 6.0 mmol). After 24 h, water (50 mL) and EtOAc (50 mL) were added, and the aqueous phase was extracted with further portions of EtOAc (3 × 25 mL). The combined organic extracts were dried (sodium sulfate), then the volatiles were removed under reduced pressure, and the residue was purified using a Biotage Isolera 4 (snap Ultra 25g cartridge, EtOAc/hexane, 1:19 to 1:1) to afford **14a** (740 mg, 51%) as a colorless oil which solidified upon standing. mp: 120-122 °C; ^{1}H NMR (400 MHz, $CDCl_3$): δ 7.10-7.00 (m, 4H, ArH), 5.37 (s, 1H, H1), 5.24 (s, 1H, H11), 4.60-4.57 (m, 1H, H5/H7), 4.52 (br s, 1H, H5/H7), 3.92-3.89 (m, 1H, H3/H9), 3.80-3.75 (m, 2H, H3/H9), 3.64 (dd, *J* = 1.7, 7.2 Hz, 1H, H3/H9), 3.28 (br s, 1H, OH), 3.15 (d, *J* = 11.6 Hz, 1H, H6), 2.36 (dd, *J* = 4.2, 16.6 Hz, 1H, H5/H7), 2.17 (ddd, *J* = 12.0, 12.0, 5.1 Hz, 1H, H6a), 1.67-1.60 (m, 1H, H5/H7), 1.41 (d, *J* = 16.6 Hz, 1H, H5/H7), 1.25-1.20 (m, 1H, H5/H7); ^{19}F NMR (376 MHz, $CDCl_3$): δ -115.56 (s, 1F); ^{13}C NMR (100 MHz, $CDCl_3$): δ 161.8 (d, *J* = 244 Hz), 144.5, 134.9 (d, *J* = 3 Hz), 132.1, 128.2, 116.3, 114.8, 104.4, 101.4, 96.6, 94.8, 73.7, 72.1, 68.3, 68.0, 42.2, 39.4, 32.0, 30.5; IR 3380, 2963, 2898, 1681, 1507, 1223 cm^{-1}; DualESI-TOF-HRMS *m/z* Calcd. for $[M + Na]^+$; $C_{19}H_{19}FO_6Na$: 385.1058; found 385.1062.

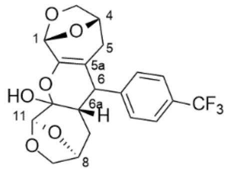

(1*R*,4S,6aS,8S,11R)-6-(4-(Trifluoromethyl)phenyl)-3,4,5,6,6a,7,8,9,11,11a-decahydro-1*H*-1,4:8,11-diepoxypyrano[2,3-*c*:6,5-*c*']bis(oxepine)-11a-ol (14b). To a stirred solution of Cyrene™ (**12**) (410 µL, 513 mg, 4.0 mmol) in acetonitrile (1 mL) was added 4-trifluoromethylbenzaldehyde (273 µL, 348 mg, 2.0 mmol) and DBU (449 µL, 3.0 mmol). The mixture was stirred at ambient

temperature for 24 h and then water (25 mL) and EtOAc (25 mL) were added. The aqueous phase was extracted with EtOAc (3 × 25 mL), and the combined organic extracts were dried (sodium sulfate) and then concentrated under reduced pressure. The residue was purified using a Biotage Isolera 4 (snap Ultra 10g cartridge, 1:19 to 1:1 EtOAc/hexanes) to afford **14b** (75 mg, 9%) as a colorless oil which solidified upon standing. mp: 227-228 °C; ^{1}H NMR (400 MHz, $CDCl_3$): δ 7.64-7.51 (m, 2H, ArH), 7.29-7.26 (m, 2H, ArH), 5.38 (s, 1H, H1), 5.31 (s, 1H, H11), 4.59-4.56 (m, 2H, H4/H8), 3.88 (t, *J* = 6.1 Hz, 1H, H3/H9), 3.80-3.75 (m, 2H, H3/H9), 3.62 (dd, *J* = 7.2, 1.6 Hz, 1H, H3/H9), 3.28 (d, *J* = 11.6 Hz, 1H, H6), 2.39-2.32 (m, H5b), 2.17 (ddd, *J* = 12.1, 12.1, 5.0 Hz, 1H, H6a), 1.72-1.65 (m, 1H, H7a), 1.41 (d, *J* = 16.7 Hz, 1H, H5a), 1.90 (br s, 1H, OH), 1.25-1.16 (m, 1H, H7b); ^{19}F NMR (376 MHz, $CDCl_3$): δ -62.45 (s, 3F); ^{13}C NMR (100 MHz, $CDCl_3$): δ 145.1, 143.6, 131.0 (br), 129.6 (d, *J* = 32 Hz), 127.0, 125.4, 122.7, 103.8, 101.4, 96.6, 94.7, 73.7, 72.1, 68.3, 68.0, 42.9, 39.3, 32.0, 30.4; IR 3402, 2901, 2360, 2341, 1682, 1618, 1323 cm^{-1}; DualESI-TOF-HRMS *m/z* Calcd. for $[M + Na]^+$; $C_{20}H_{19}F_3O_6Na$: 435.1026; found 435.1028.

^{1}H NMR (500 MHz, $CDCl_3$) for **5a**

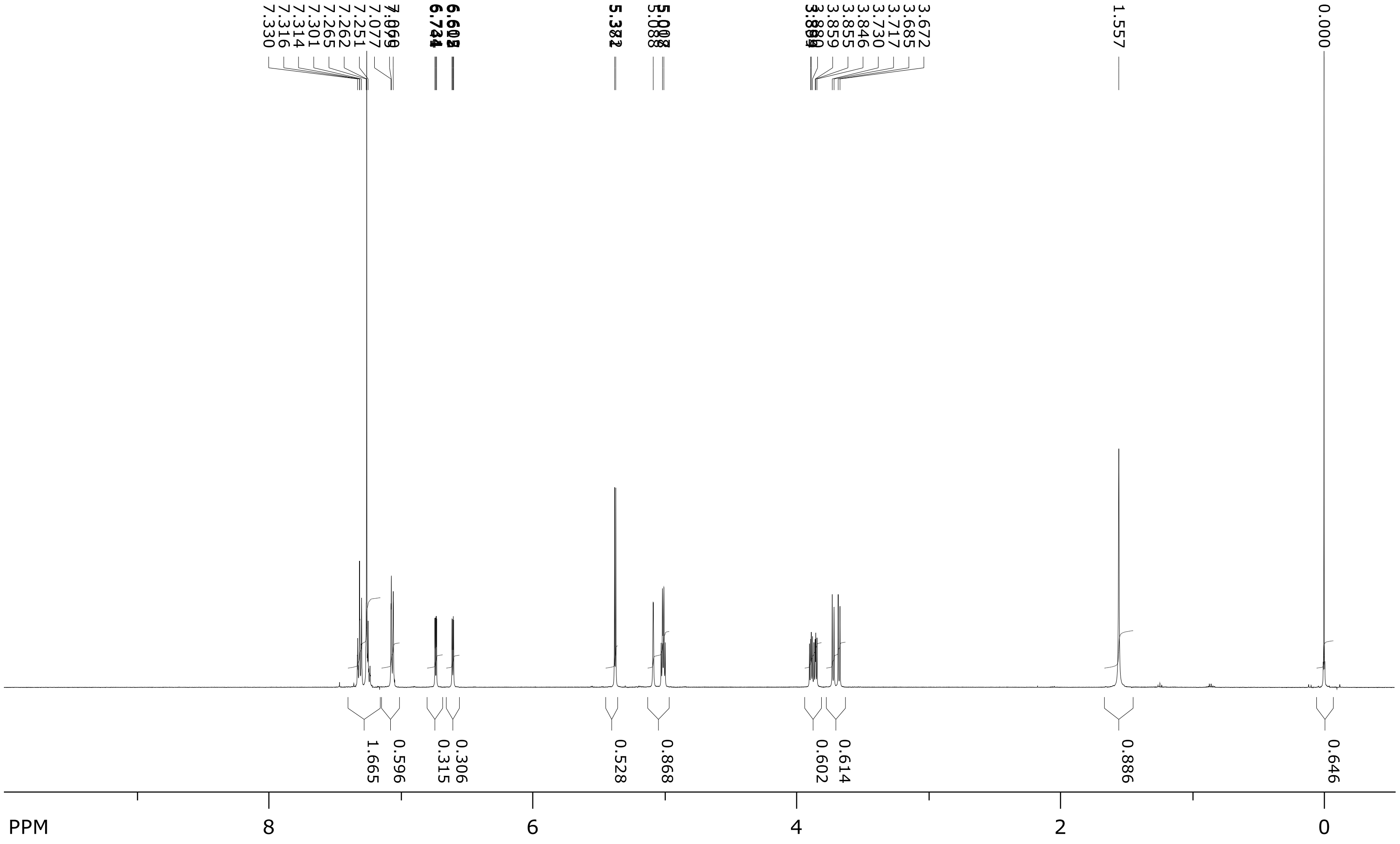

^{13}C NMR (125 MHz, $CDCl_3$) for **5a**

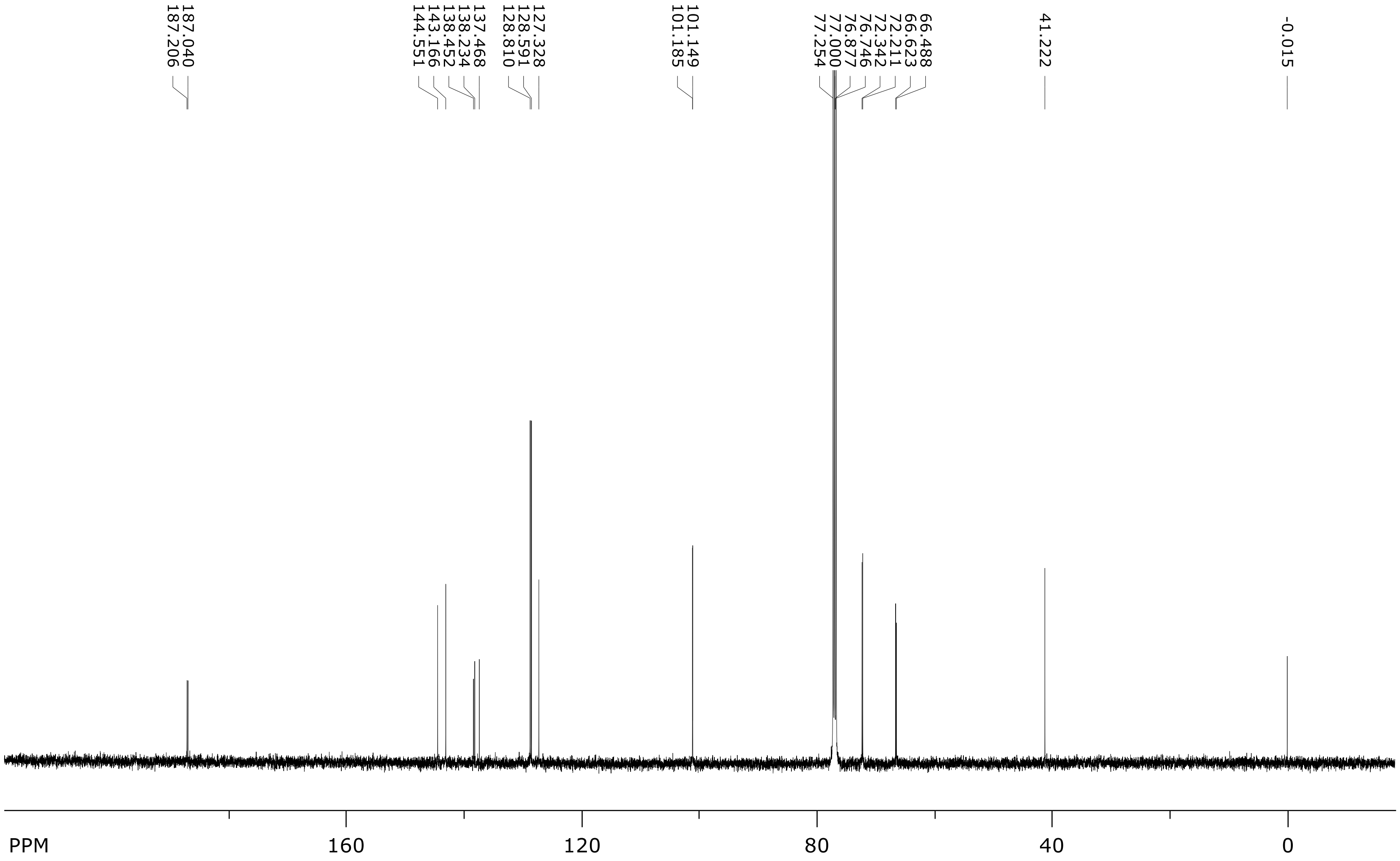

COSY NMR for **5a** with assignments

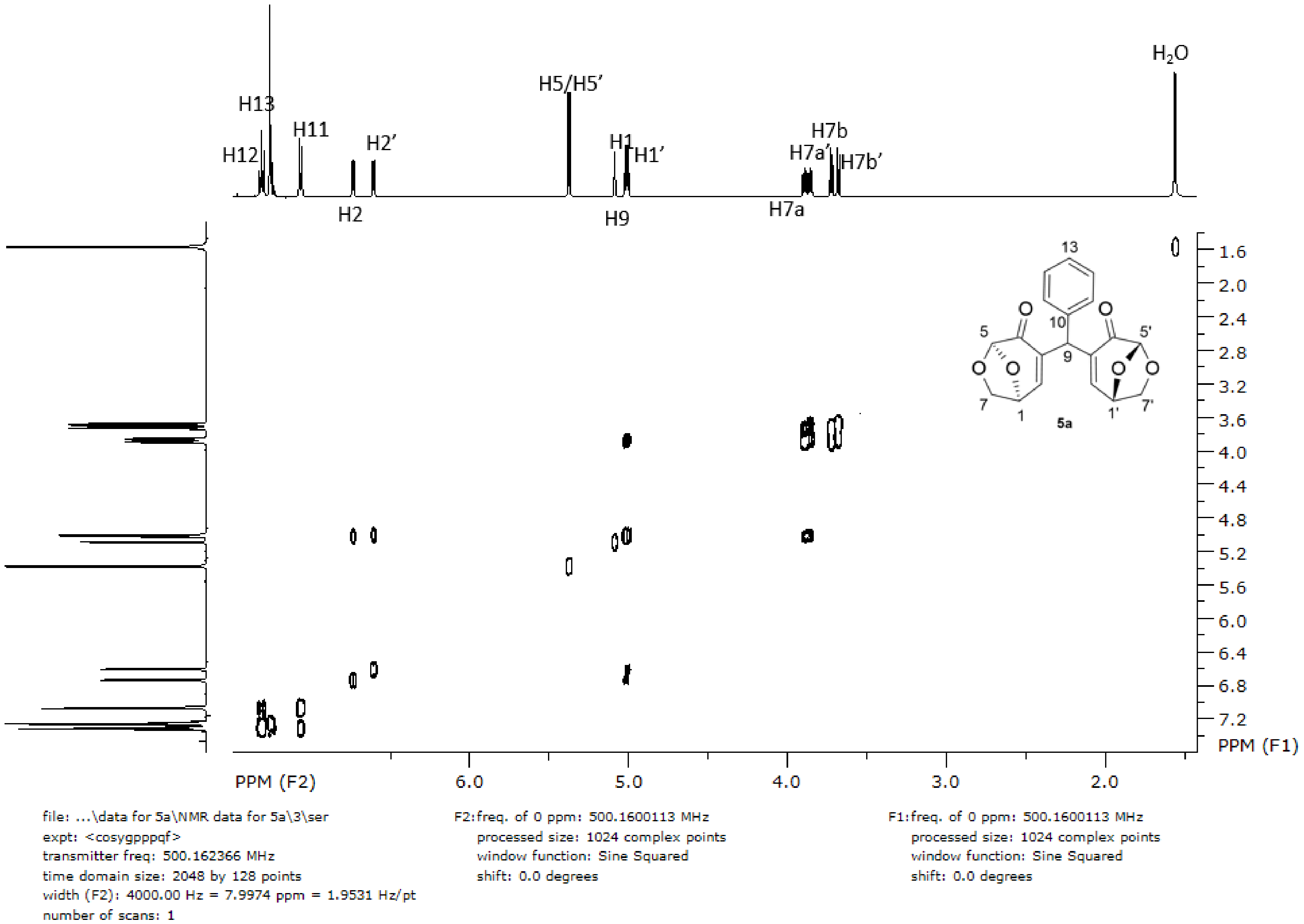

HSQC NMR for **5a** with assignments

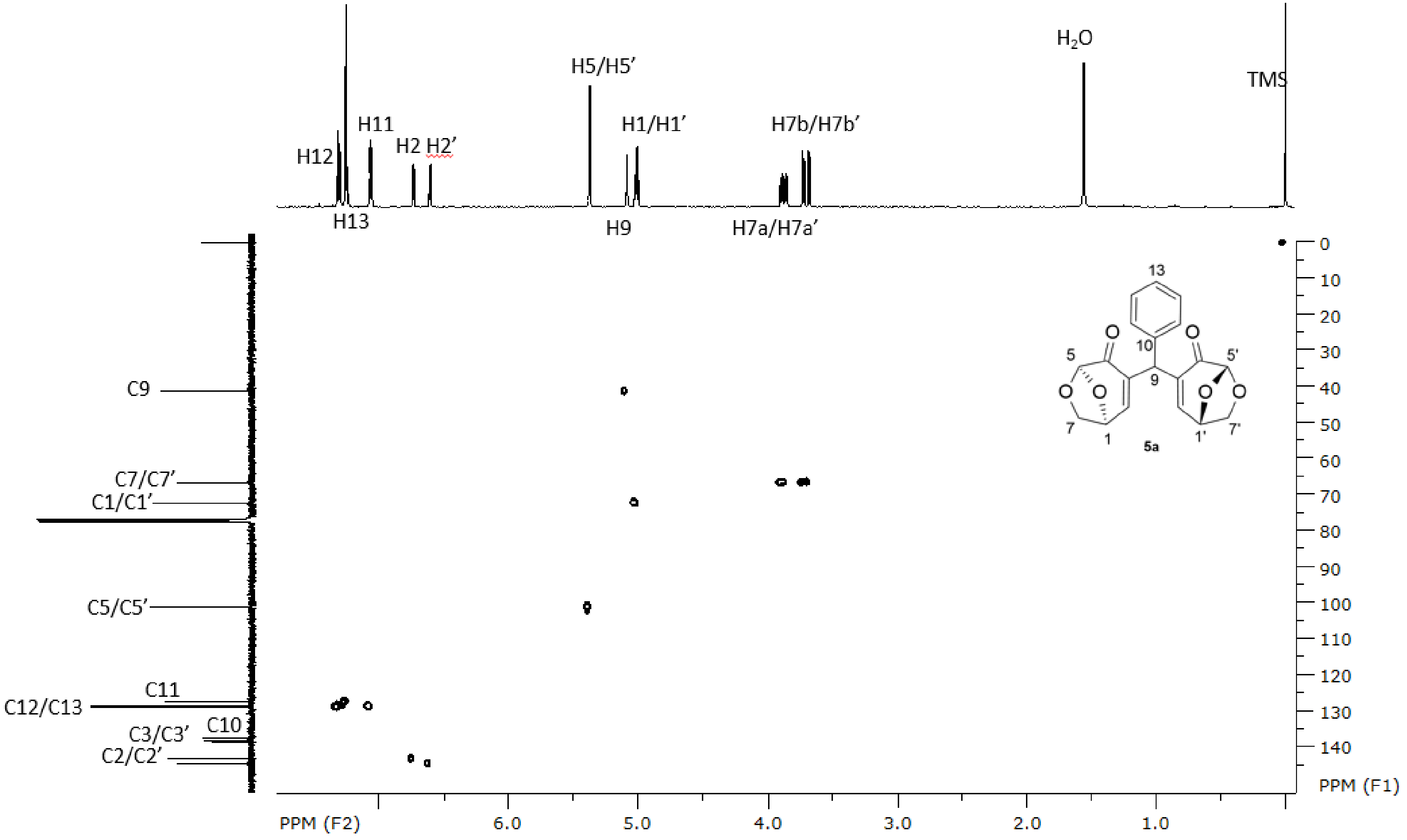

file: ...\data for 5a\NMR data for 5a\5\ser
expt: <hsqcedetgpsisp2.3>
transmitter freq: 500.162351 MHz
time domain size: 2048 by 256 points
width (F2): 8012.82 Hz = 16.0204 ppm = 3.9125 Hz/pt
number of scans: 4

F2: freq. of 0 ppm: 500.1600000 MHz
processed size: 1024 complex points
window function: Sine Squared
shift: 90.0 degrees

F1: freq. of 0 ppm: 125.7653320 MHz
processed size: 1024 complex points
window function: Sine Squared
shift: 90.0 degrees

HMBC NMR spectrum for **5a**

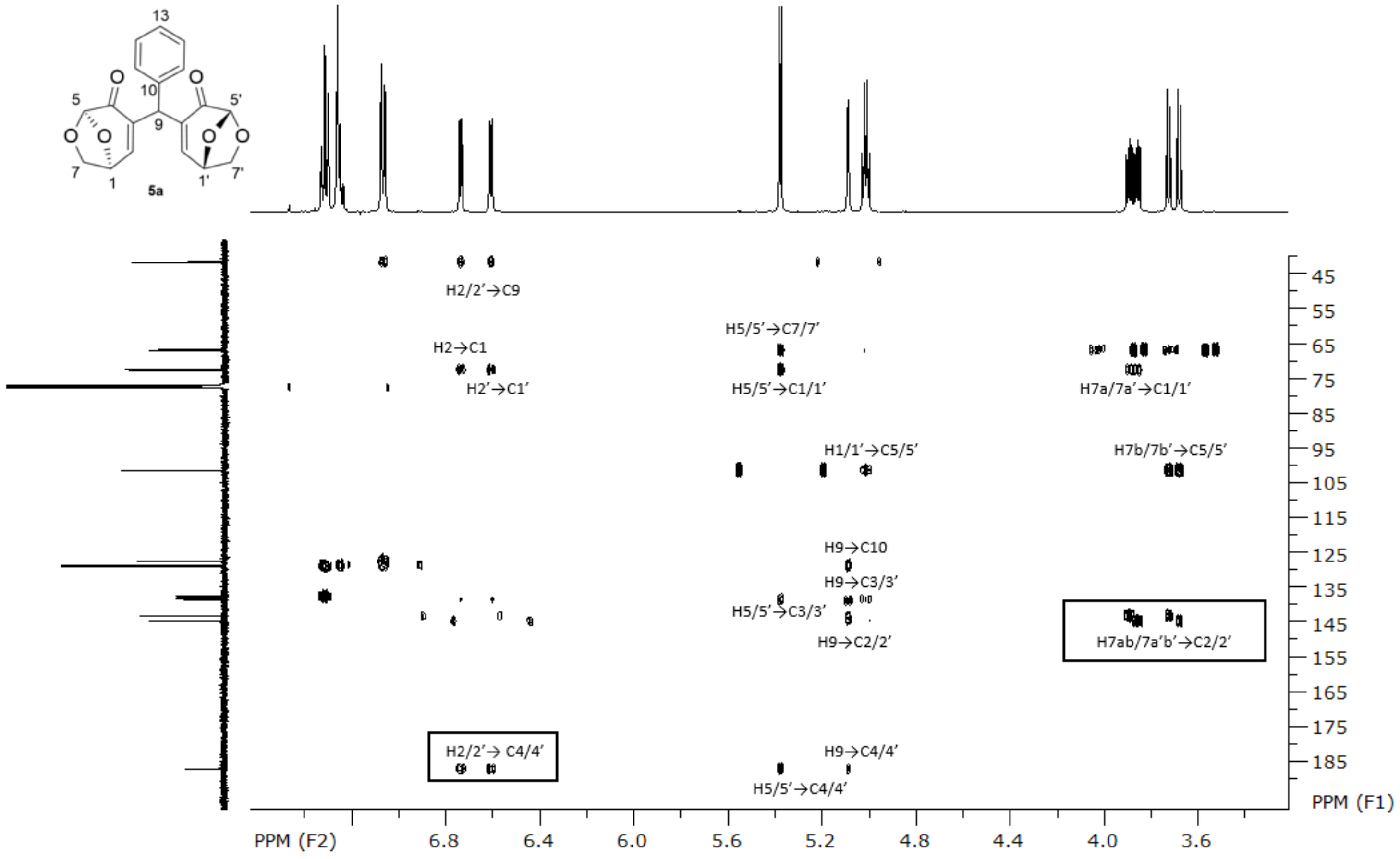

file: ...\data for 5a\NMR data for 5a\7\ser
expt: <hmbcgpndqf>
transmitter freq: 500.162366 MHz
time domain size: 4096 by 128 points
width (F2): 4000.00 Hz = 7.9974 ppm = 0.9766 Hz/pt
number of scans: 8

F2: freq. of 0 ppm: 500.1600113 MHz
processed size: 4096 complex points
window function: Sine
shift: 0.0 degrees

F1: freq. of 0 ppm: 125.7653320 MHz
processed size: 1024 complex points
window function: Sine
shift: 0.0 degrees

^{1}H NMR (500 MHz, $CDCl_3$) for **5b**

SpinWorks 4:

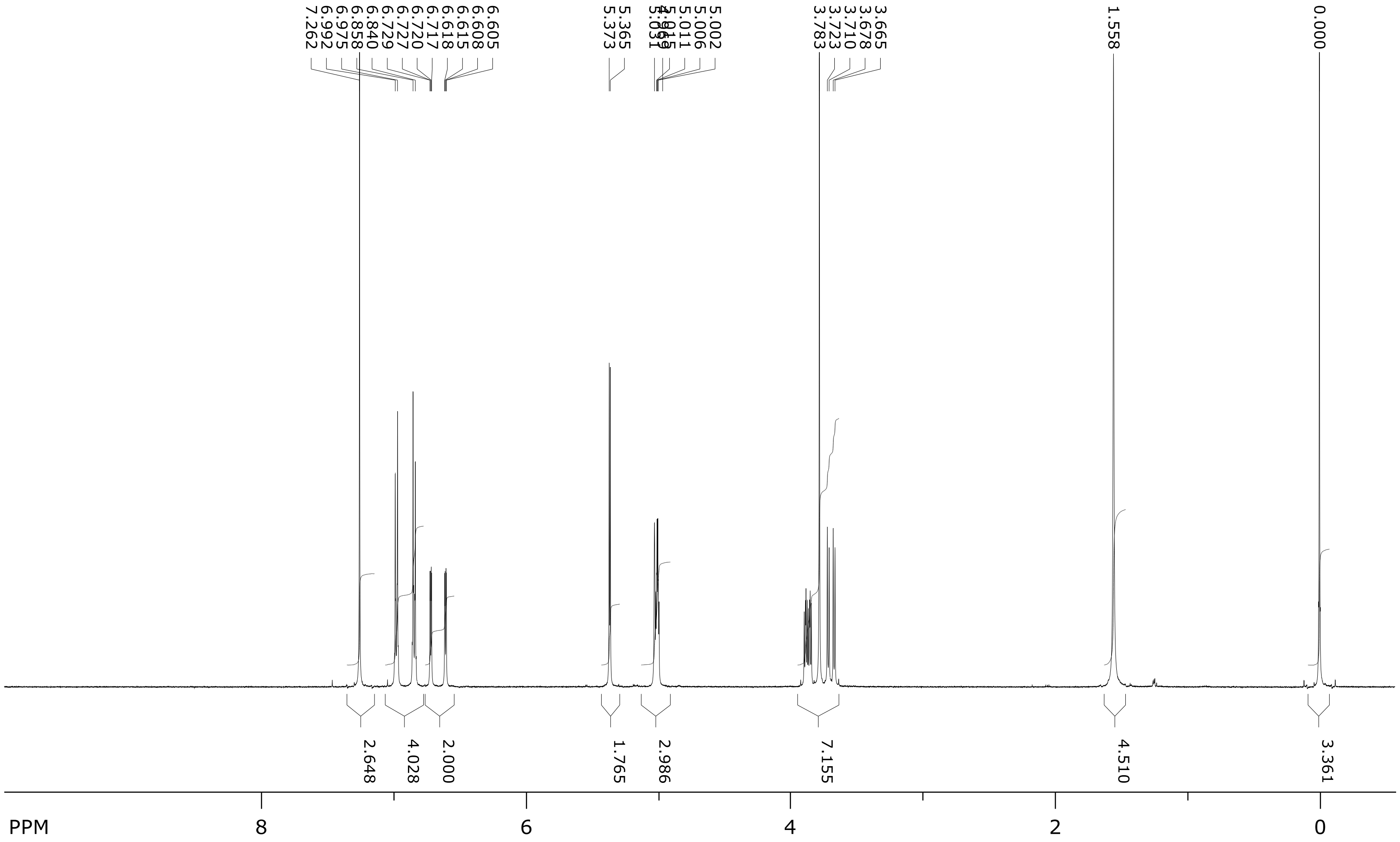

^{13}C NMR (125 MHz, $CDCl_3$) for **5b**

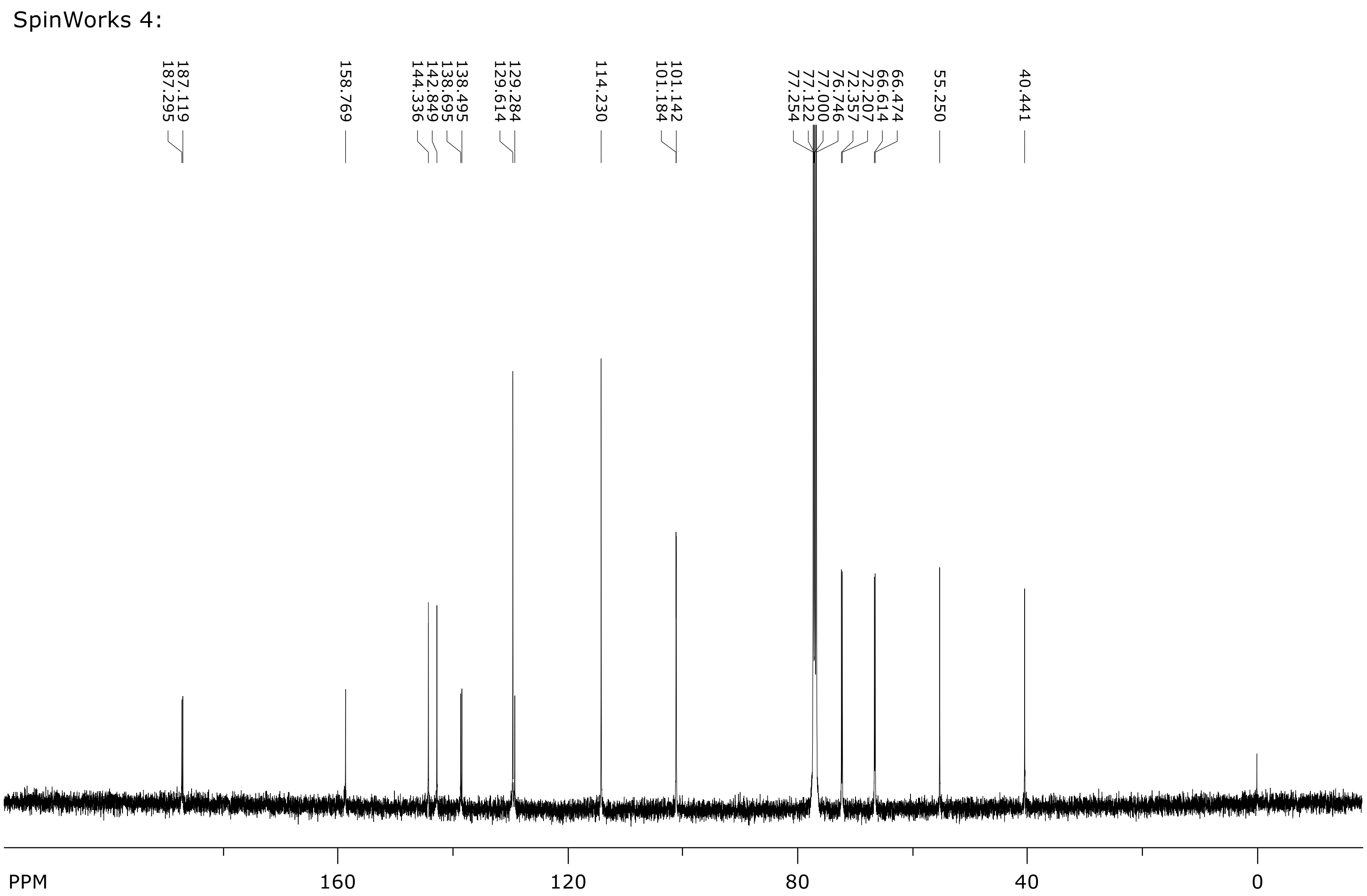

^{1}H NMR (500 MHz, $CDCl_3$) for **5c**

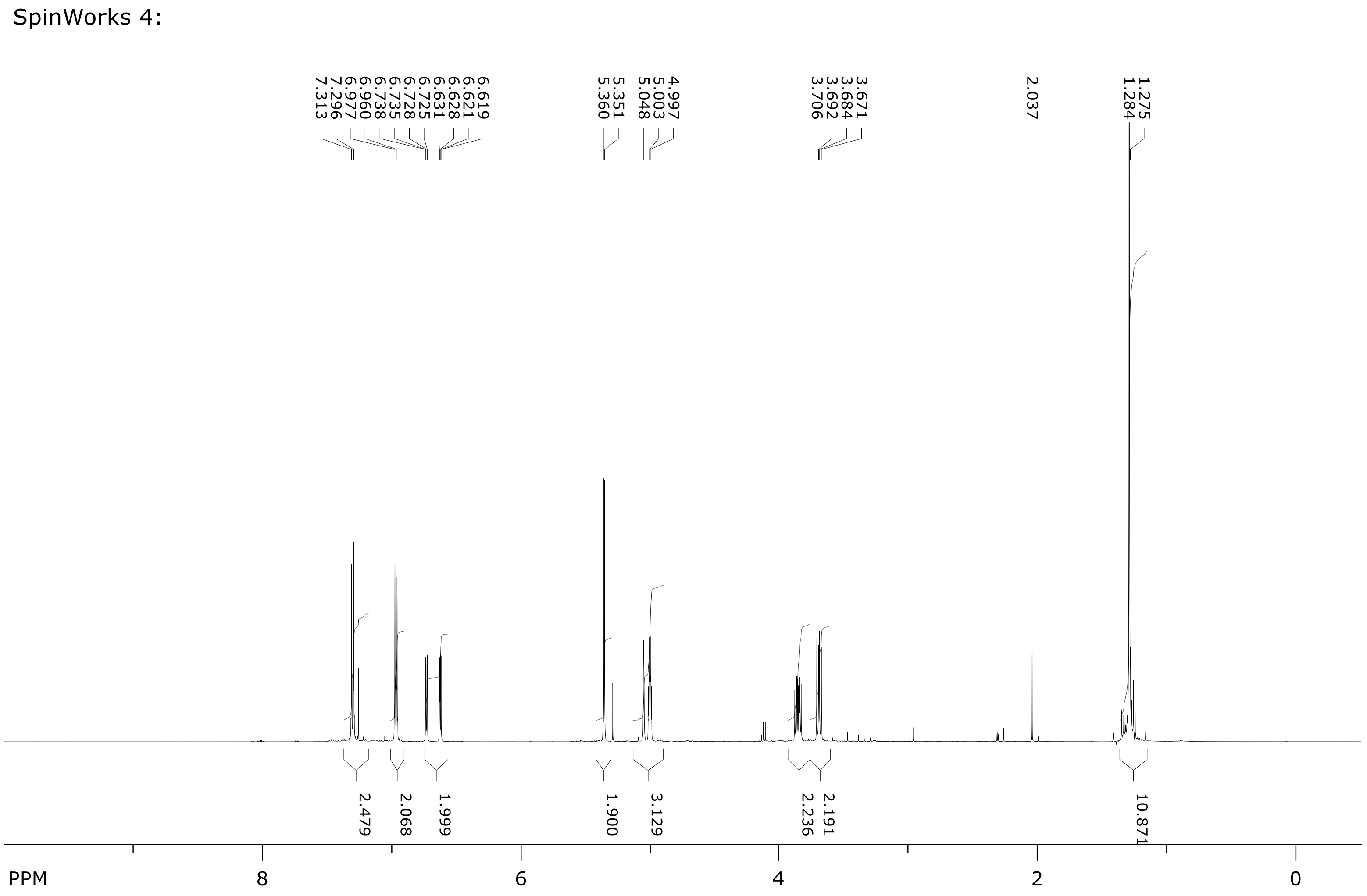

^{13}C NMR (125 MHz, $CDCl_3$) for **5c**

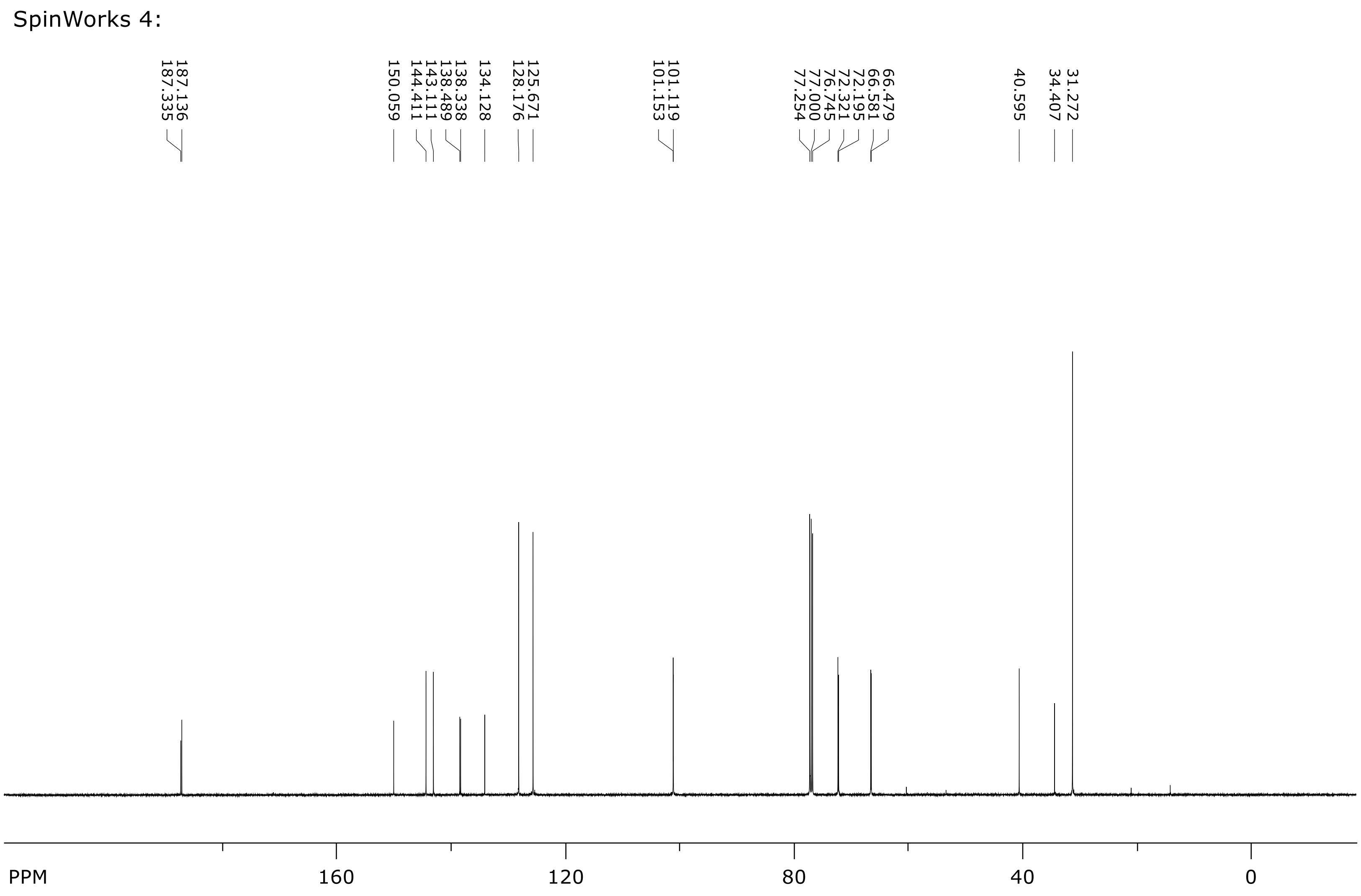

^{1}H NMR (500 MHz, $CDCl_3$) for **5d**

SpinWorks 4:

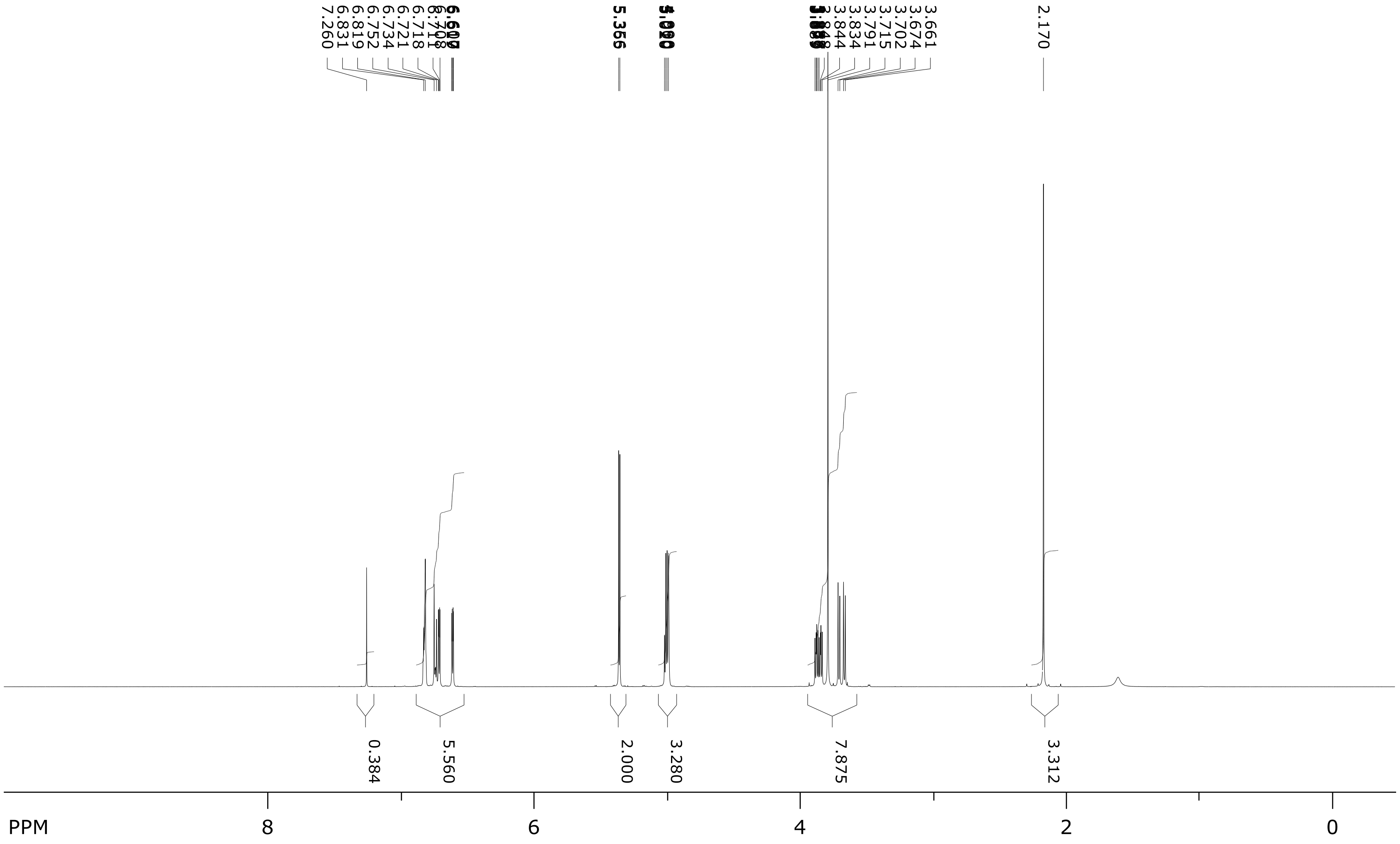

^{13}C NMR (125 MHz, $CDCl_3$) for **5d**

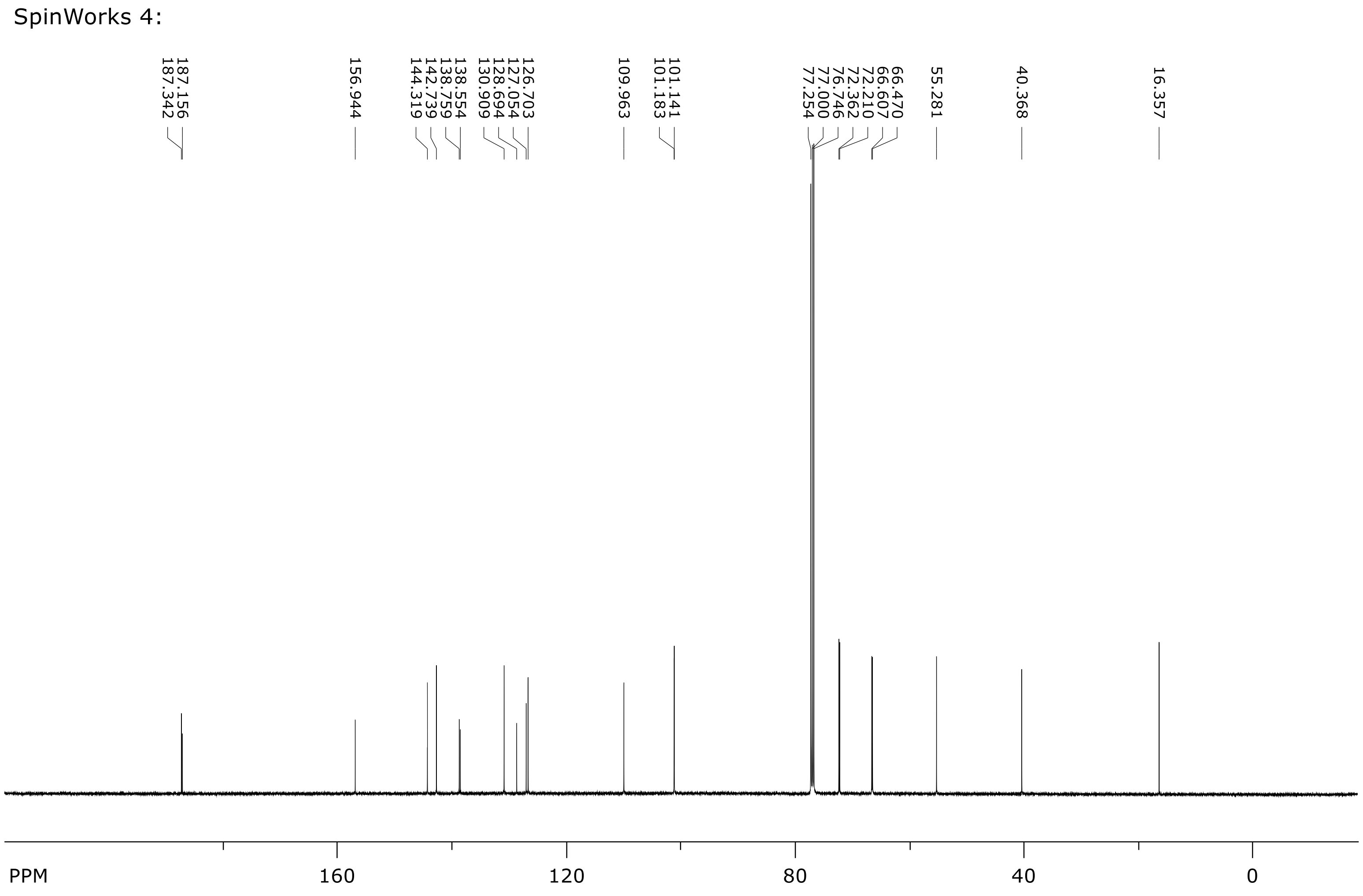

^{1}H NMR (500 MHz, $CDCl_3$) for **5e**

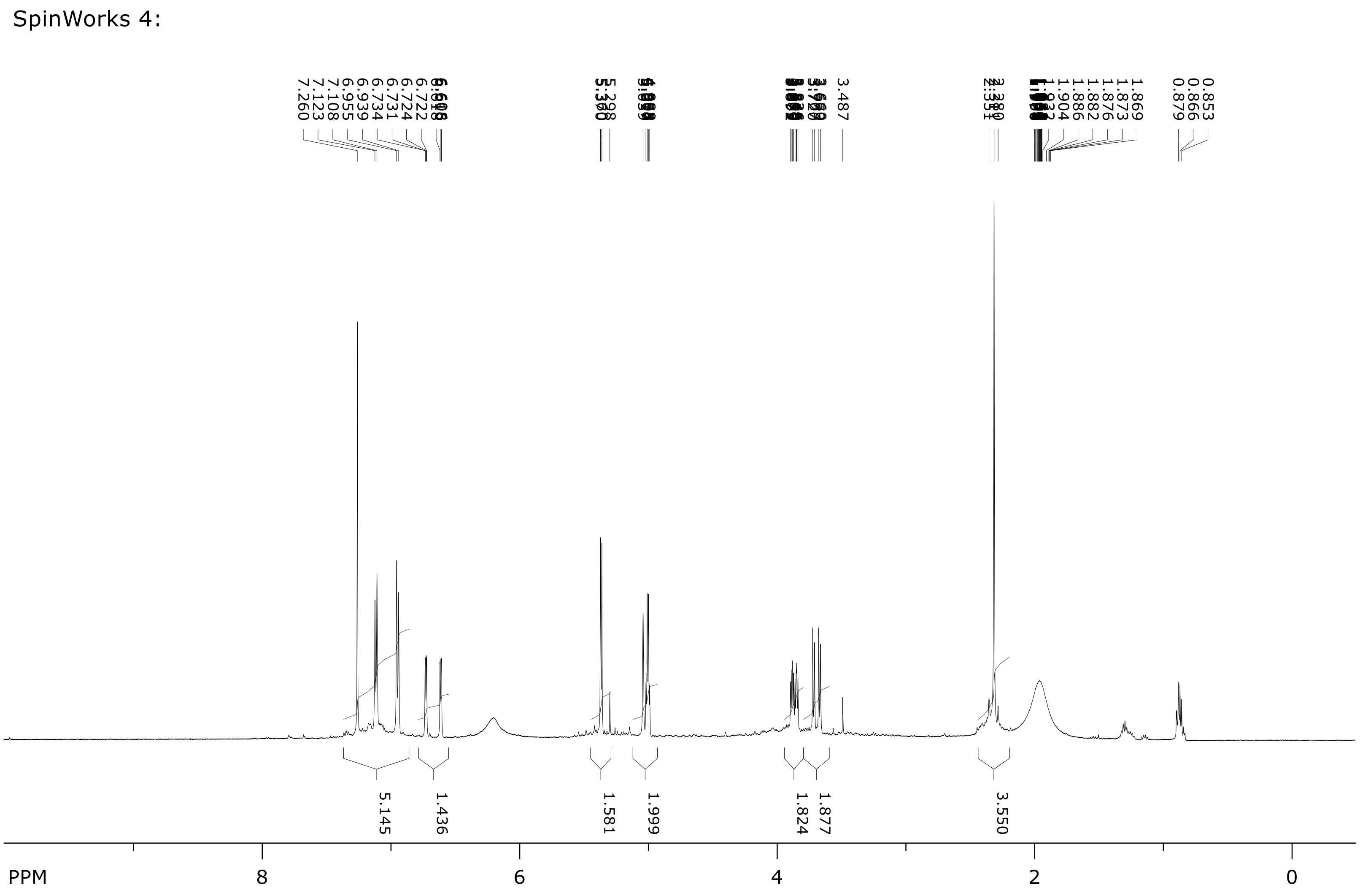

^{13}C NMR (125 MHz, $CDCl_3$) for **5e**

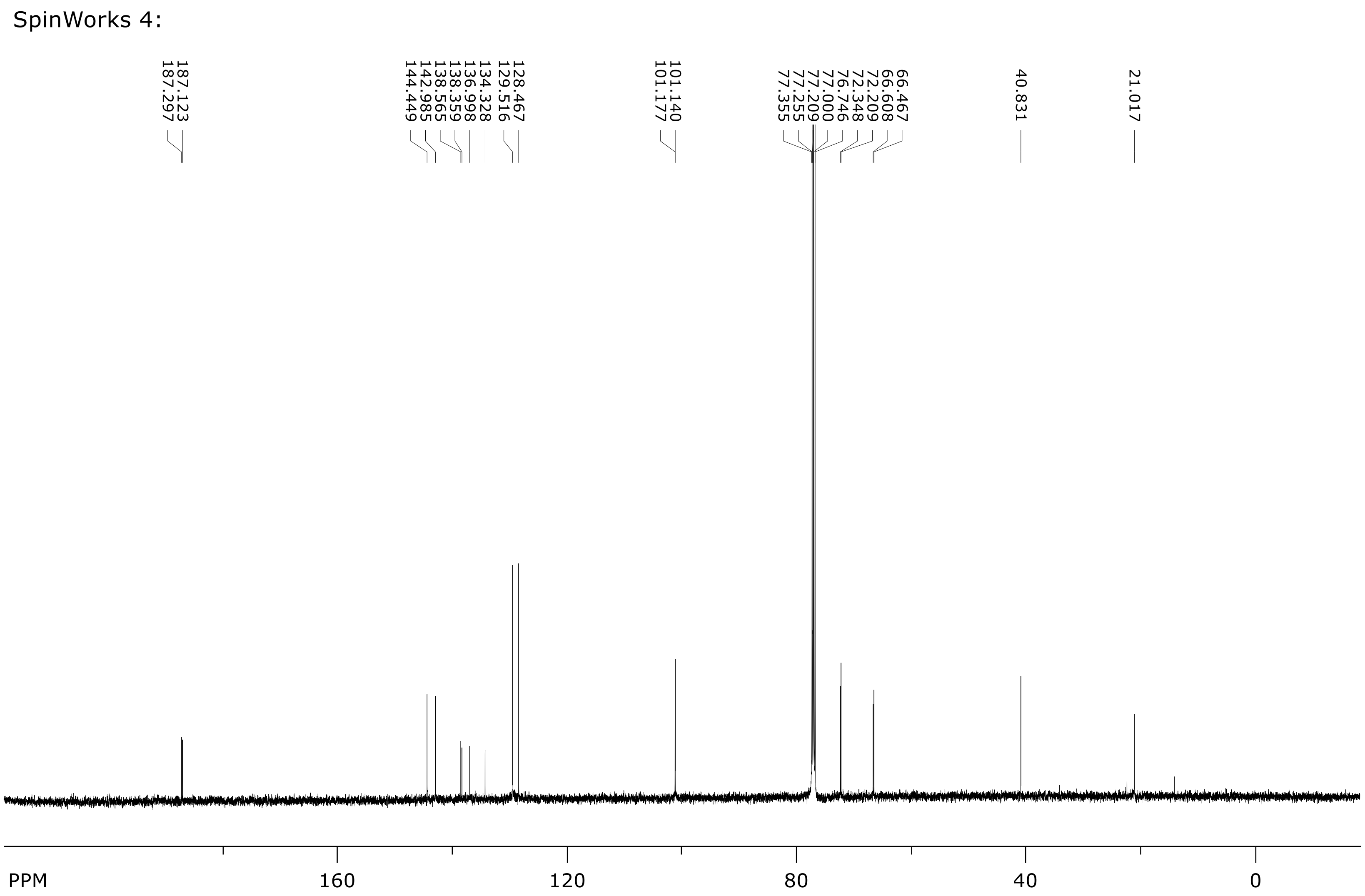

^{1}H NMR (500 MHz, $CDCl_3$) for **5f**

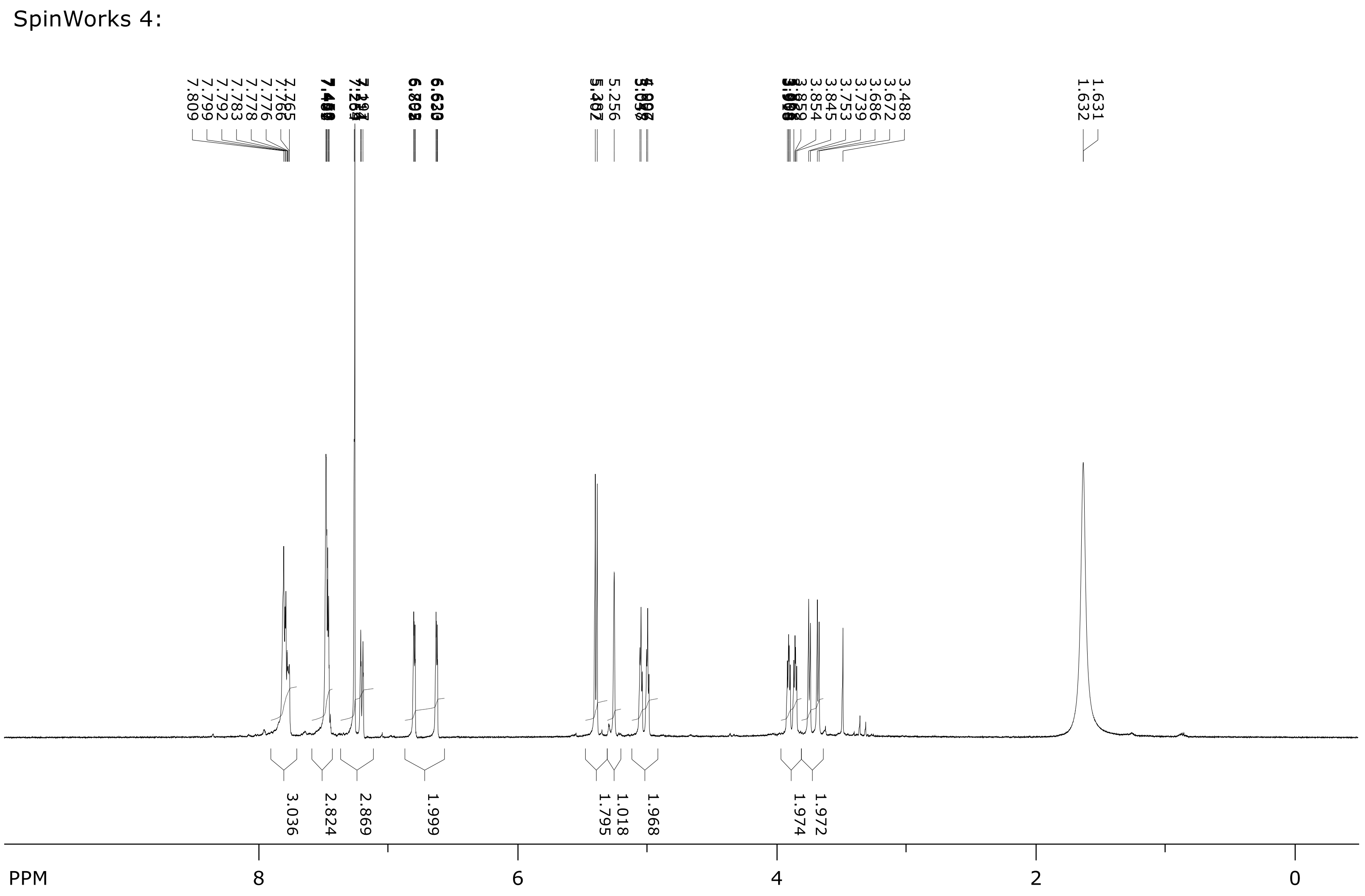

^{13}C NMR (125 MHz, $CDCl_3$) for **5f**

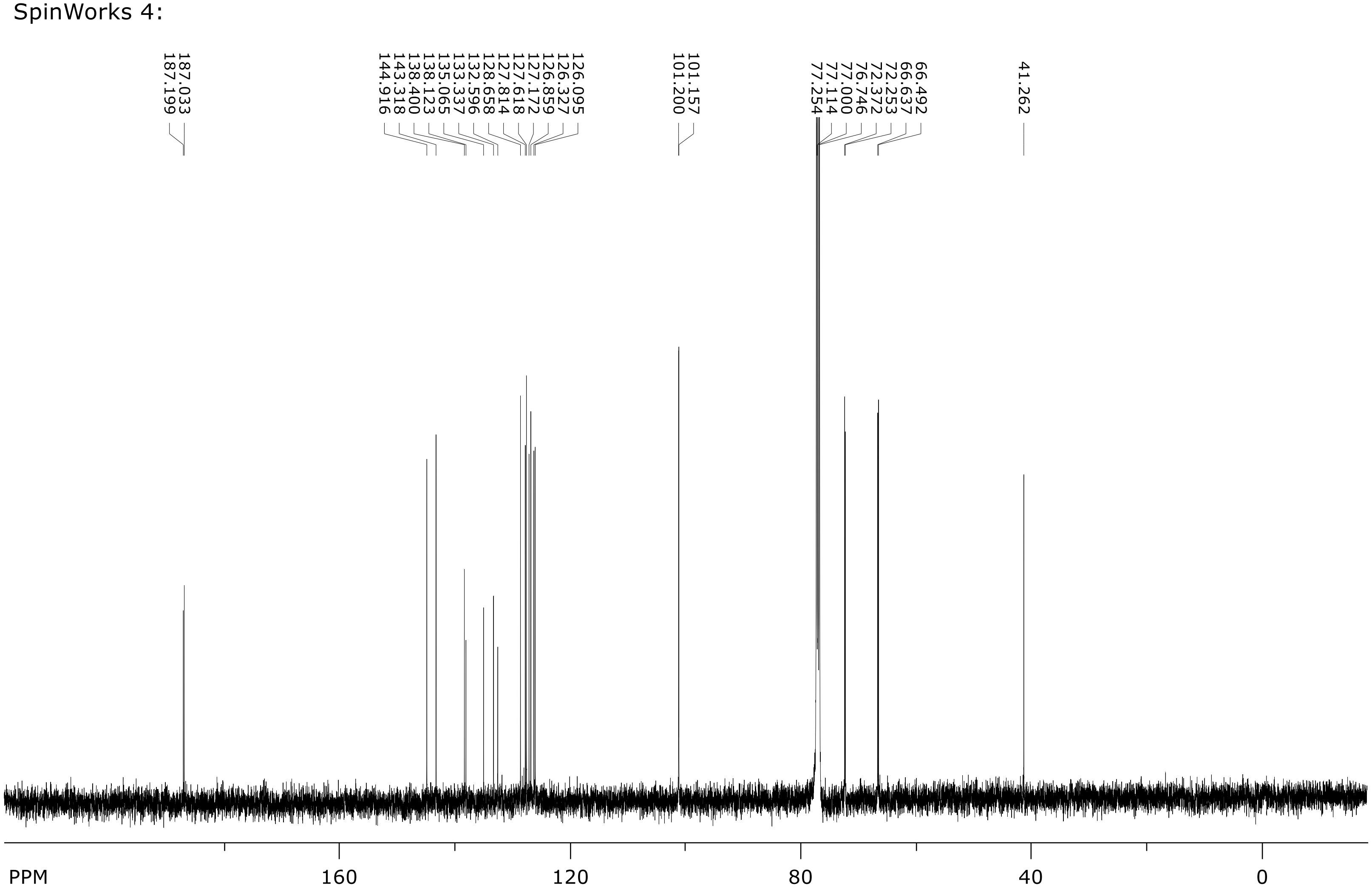

^{1}H NMR (500 MHz, $CDCl_3$) for **5g**

SpinWorks 4:

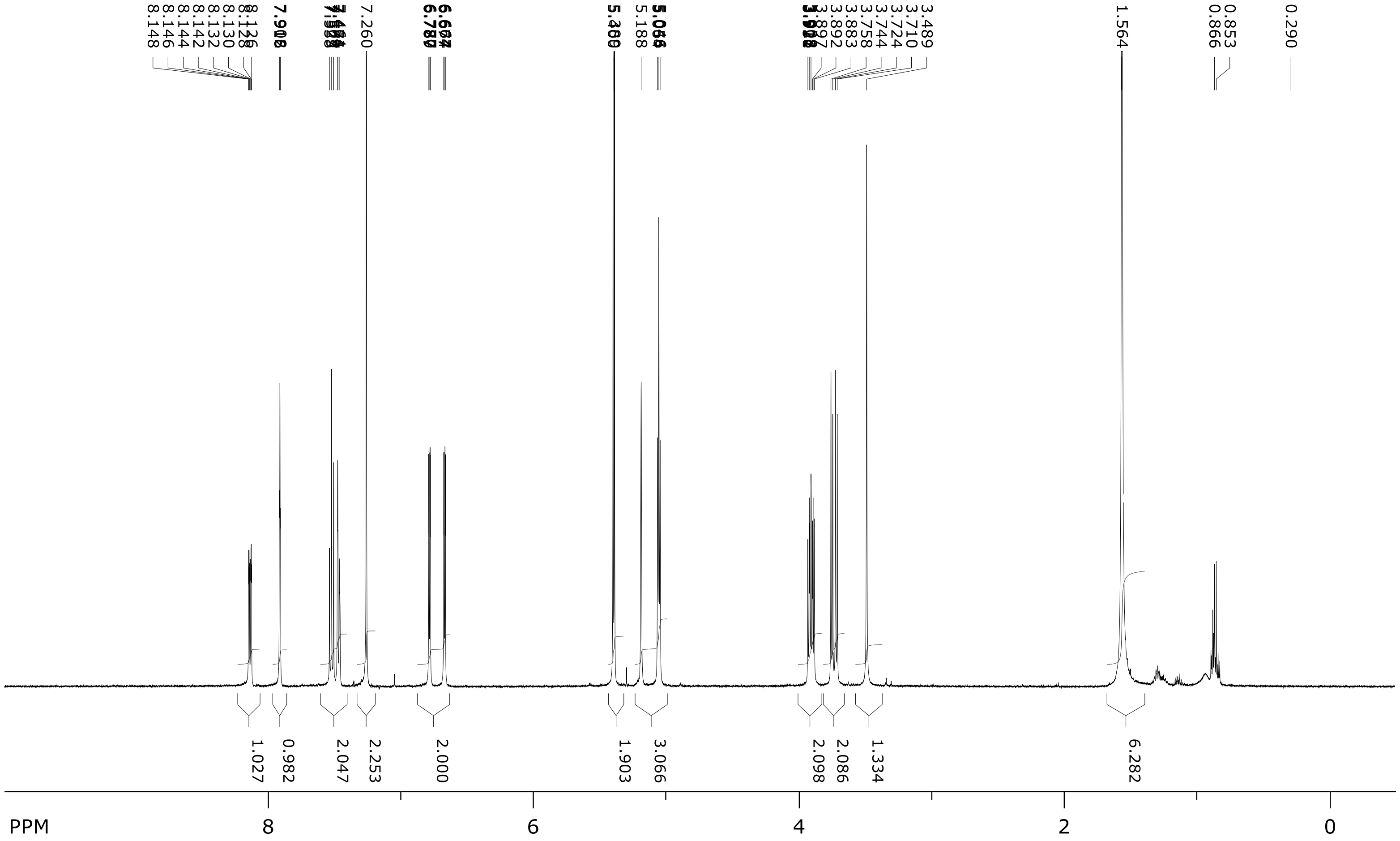

^{13}C NMR (125 MHz, $CDCl_3$) for **5g**

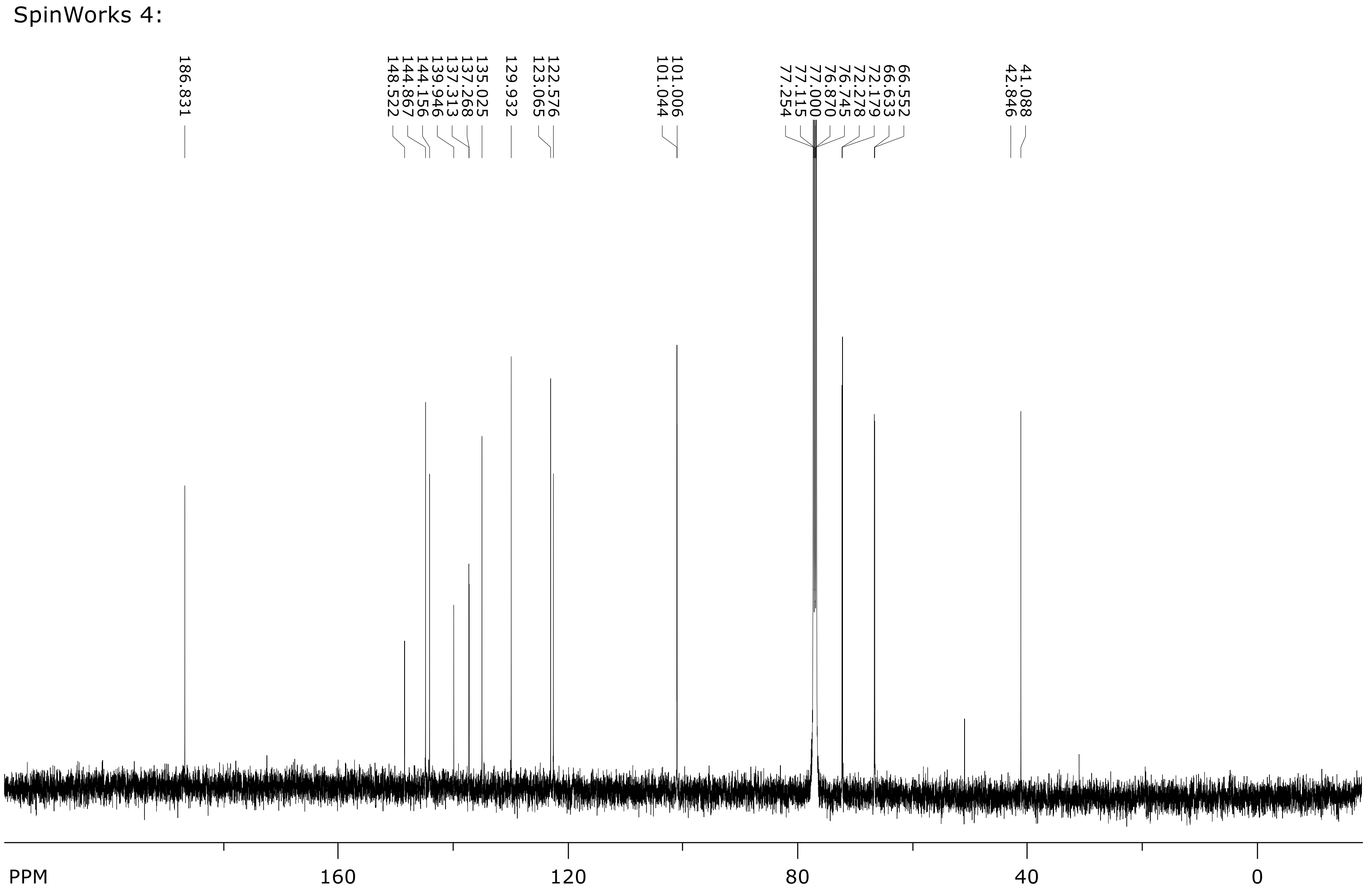

^{1}H NMR (500 MHz, $CDCl_3$) for **5h**

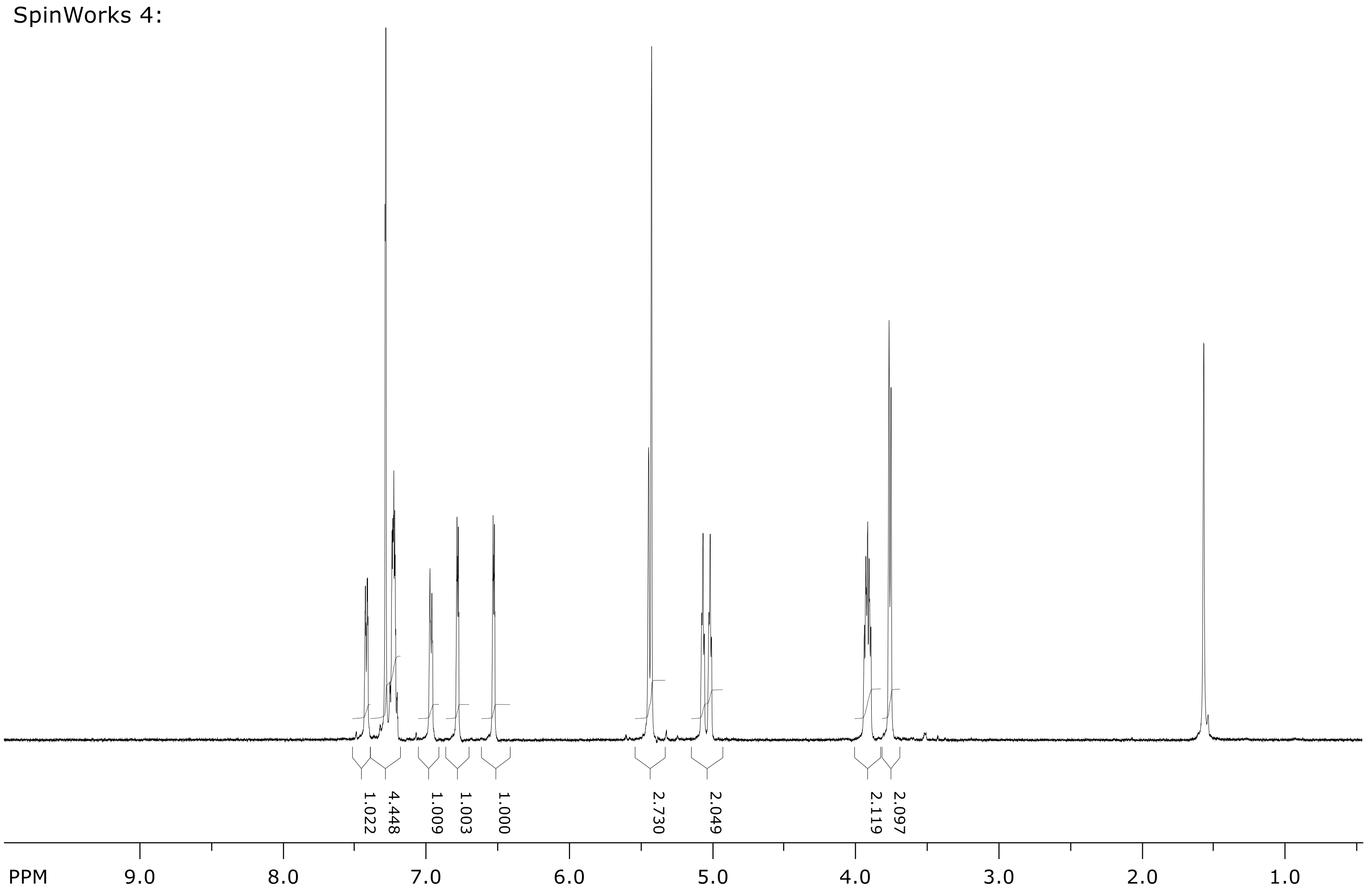

^{13}C NMR (125 MHz, $CDCl_3$) for **5h**

SpinWorks 4:

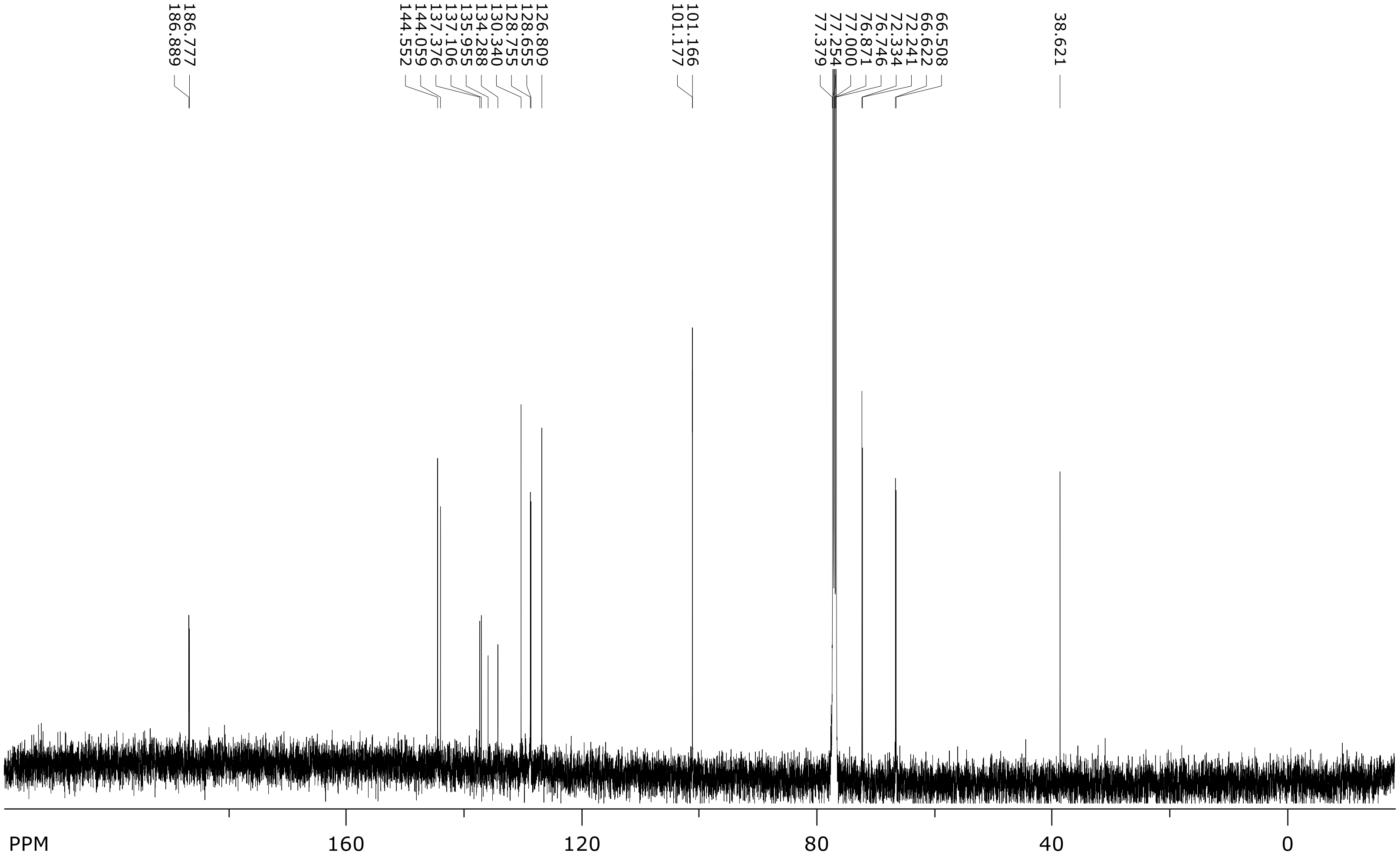

^{1}H NMR (500 MHz, $CDCl_3$) for **5i**

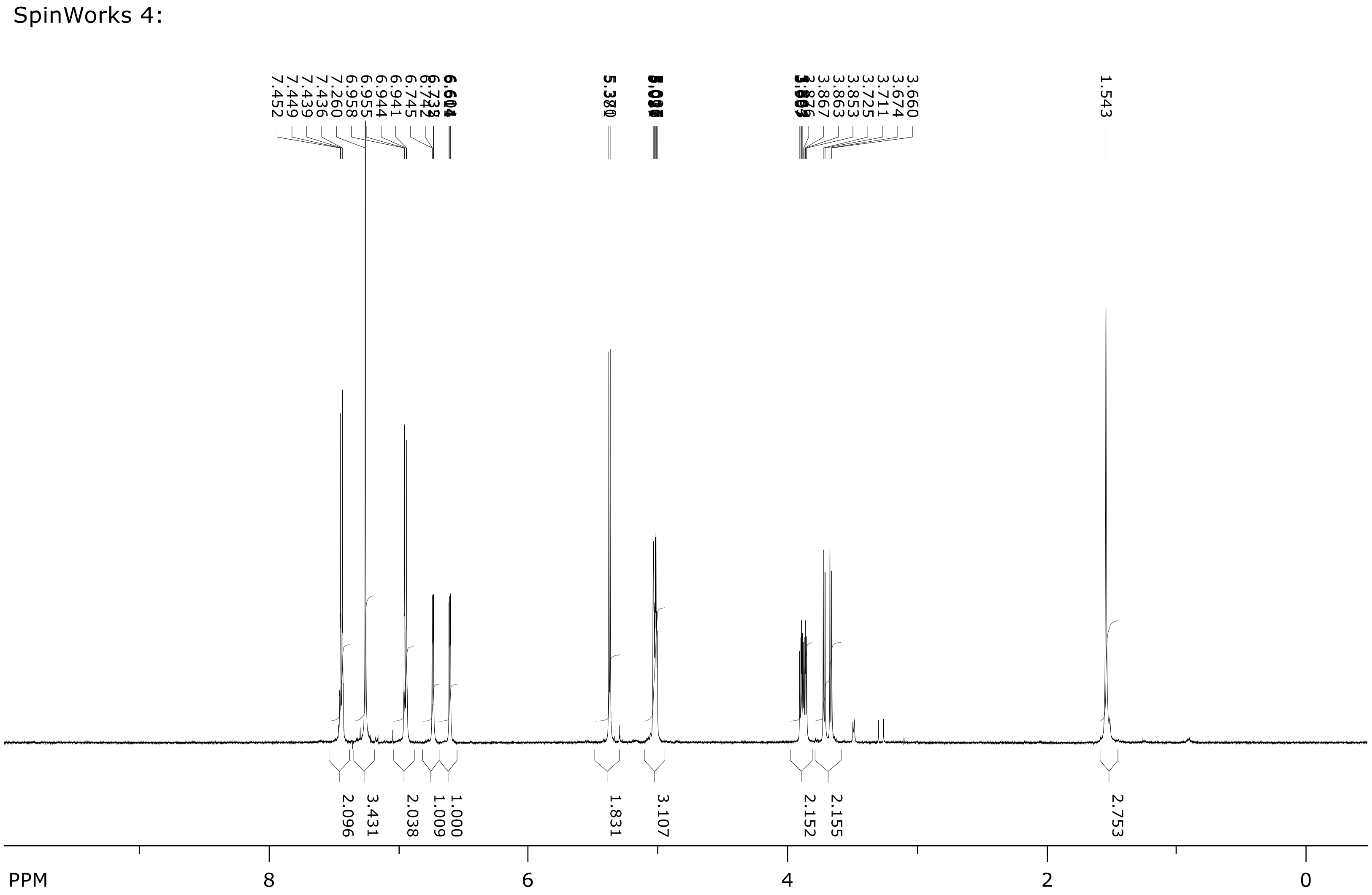

^{13}C NMR (125 MHz, $CDCl_3$) for **5i**

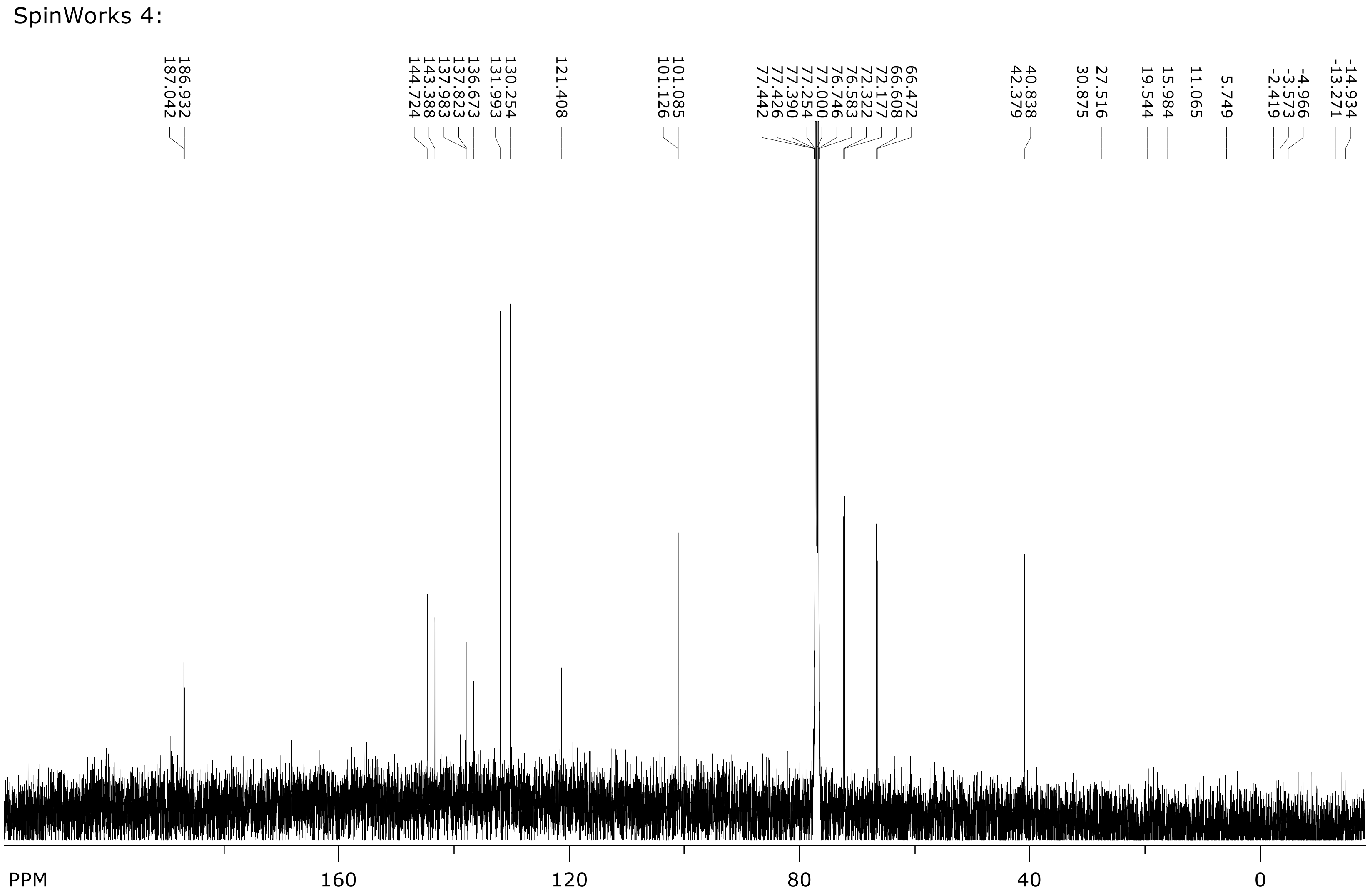

^{1}H NMR (500 MHz, $CDCl_3$) for **5j**

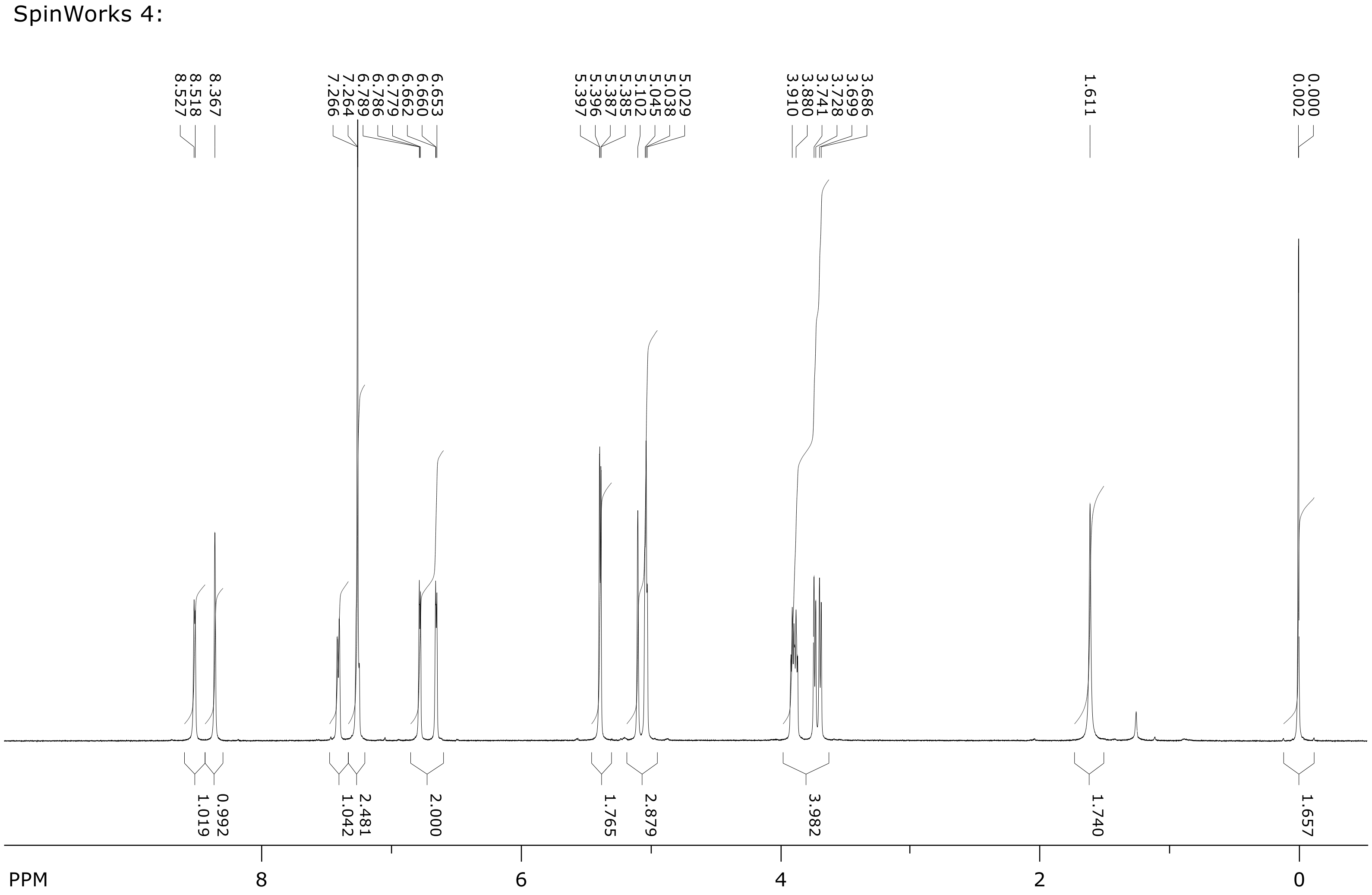

^{13}C NMR (125 MHz, $CDCl_3$) for **5j**

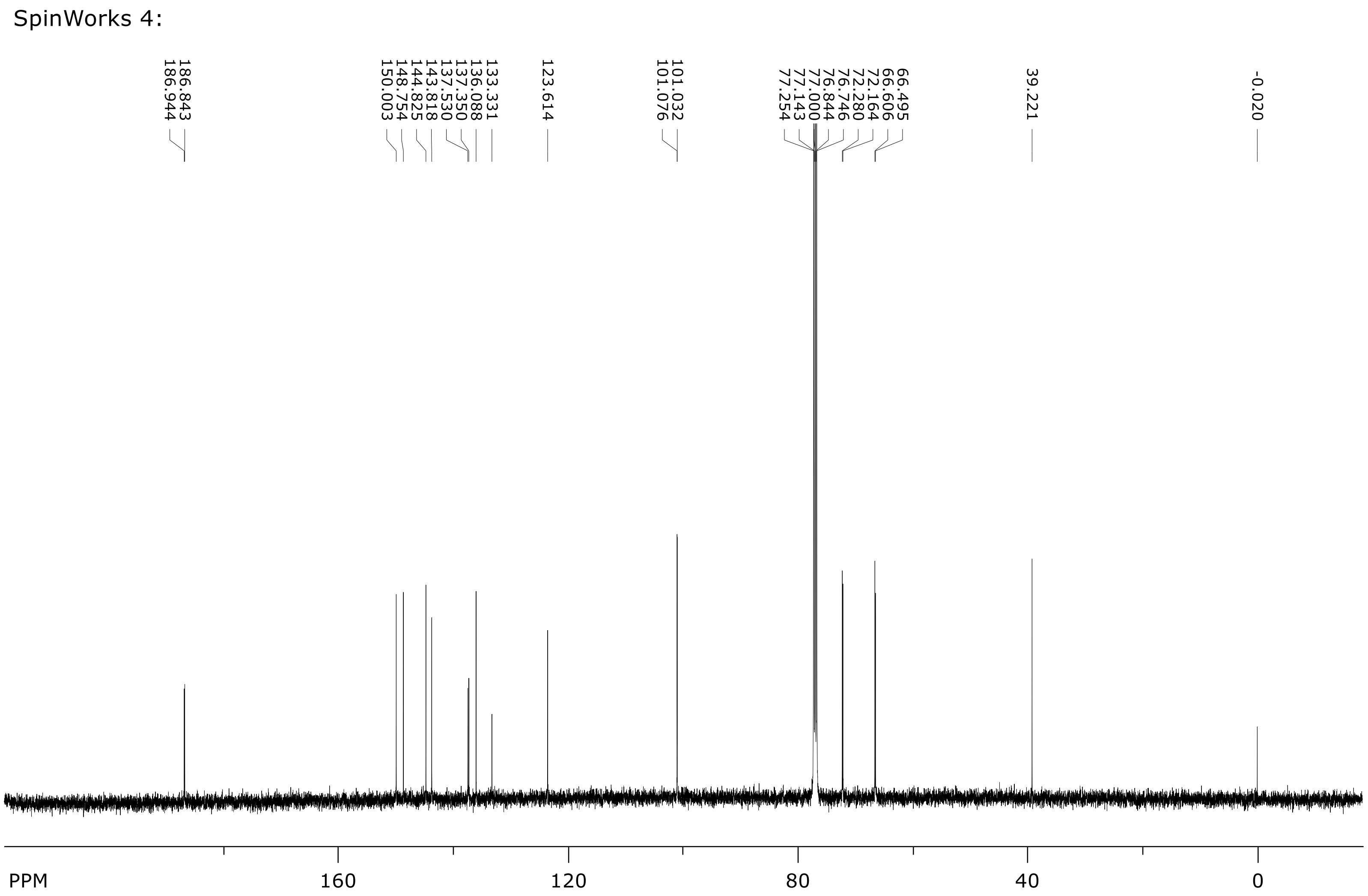

^{1}H NMR (500 MHz, $CDCl_3$) for **5k**

SpinWorks 4:

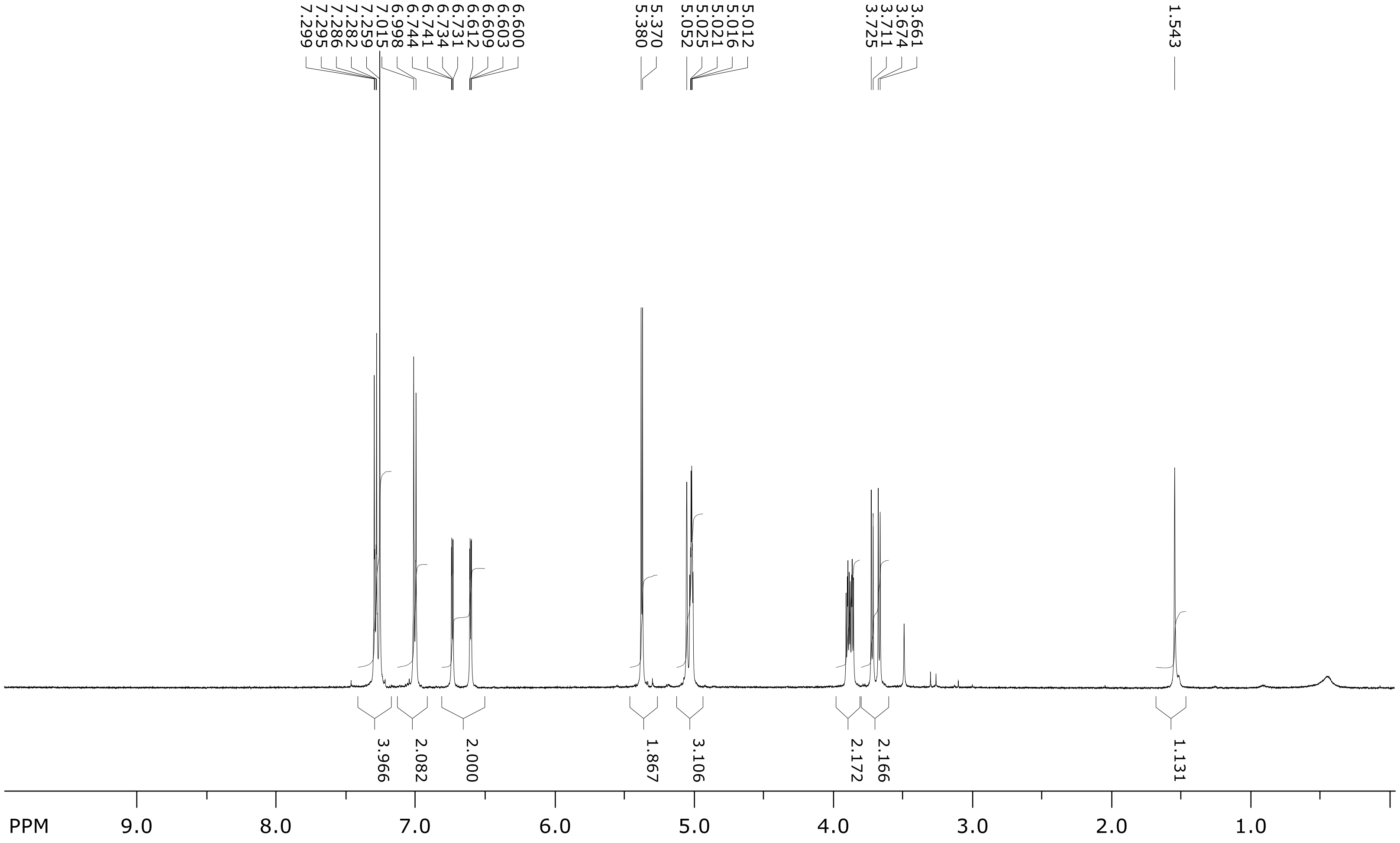

^{13}C NMR (125 MHz, $CDCl_3$) for **5k**

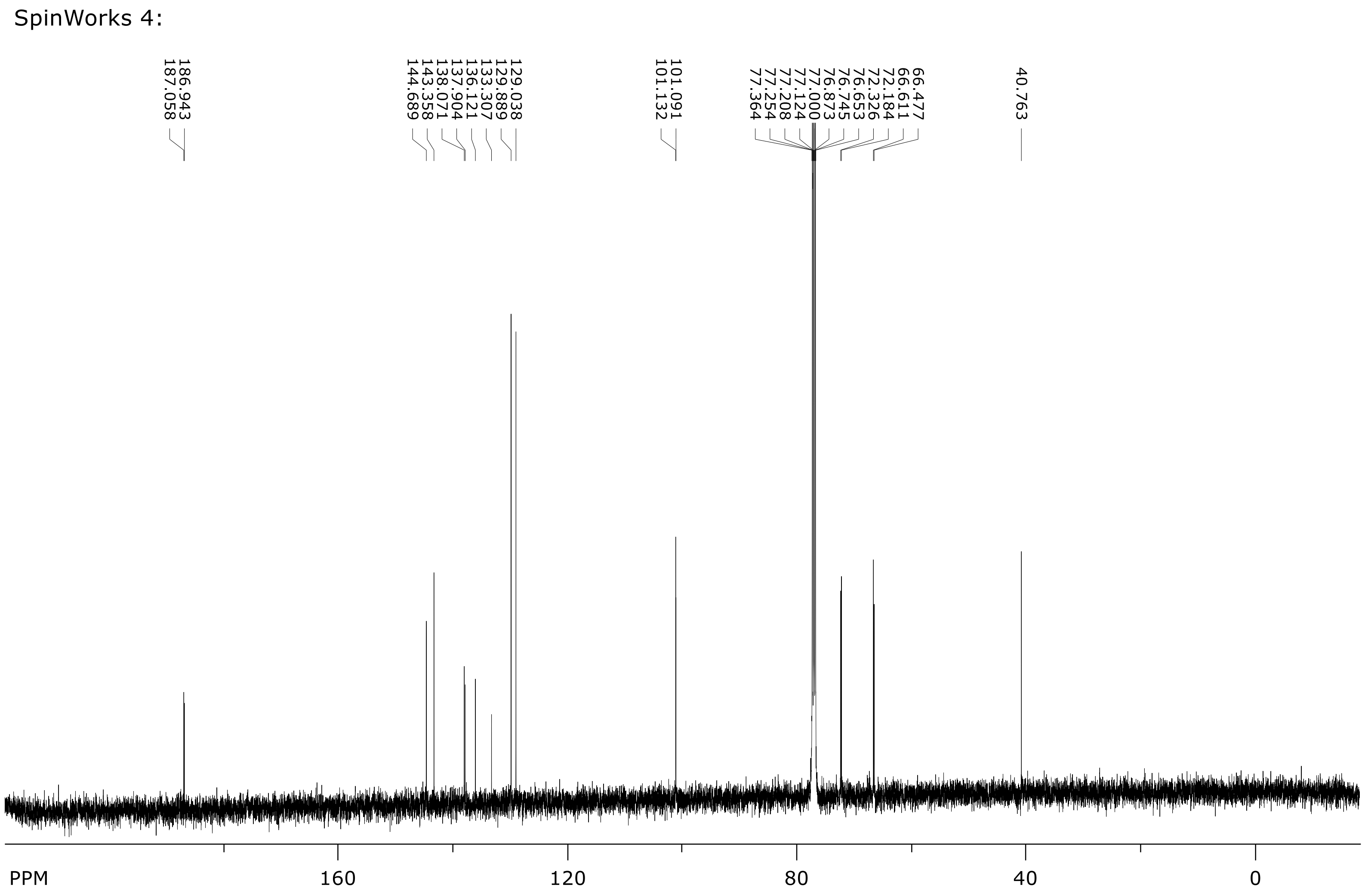

^{1}H NMR (500 MHz, $CDCl_3$) for **5l**

SpinWorks 4:

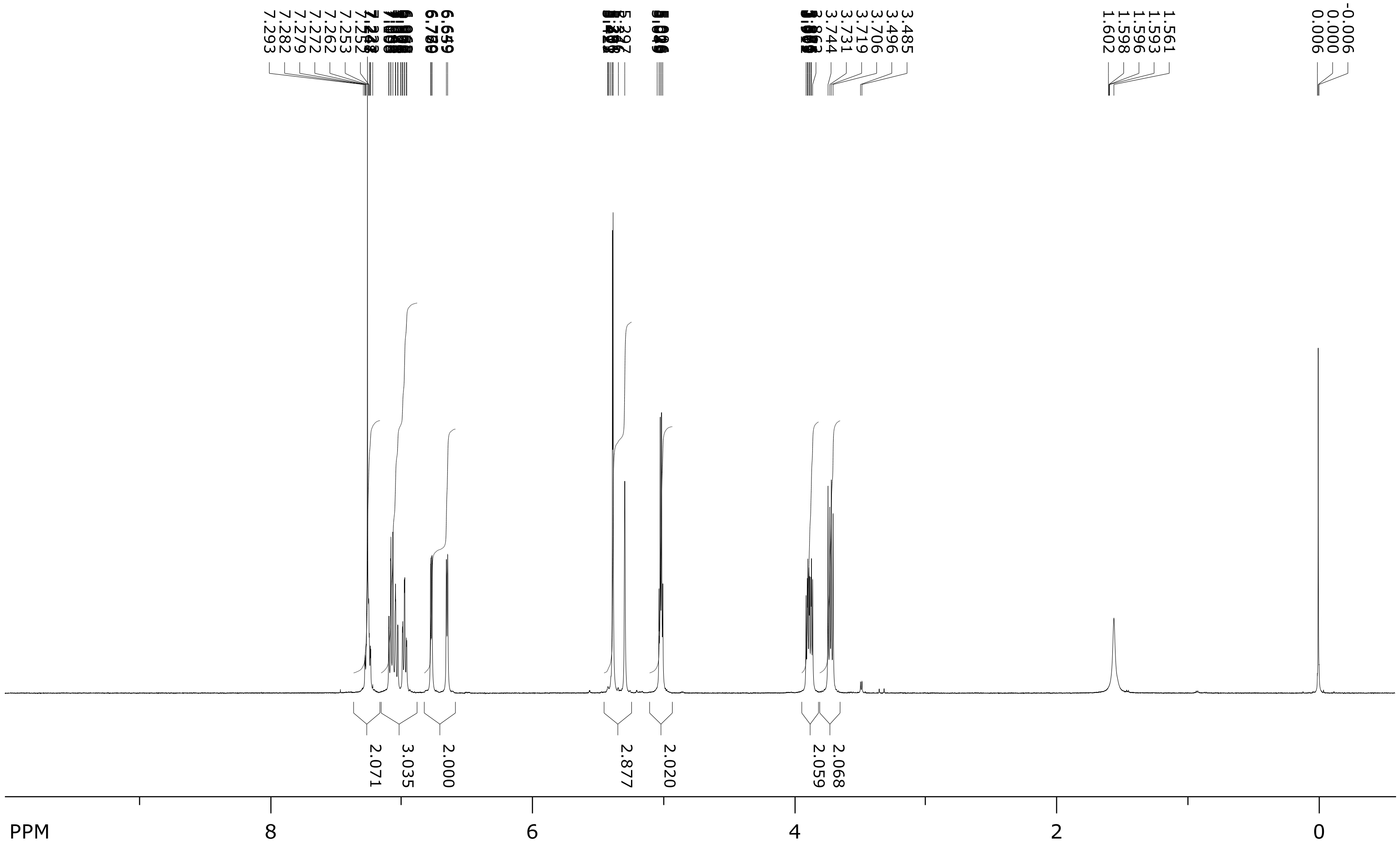

^{13}C NMR (125 MHz, $CDCl_3$) for **5l**

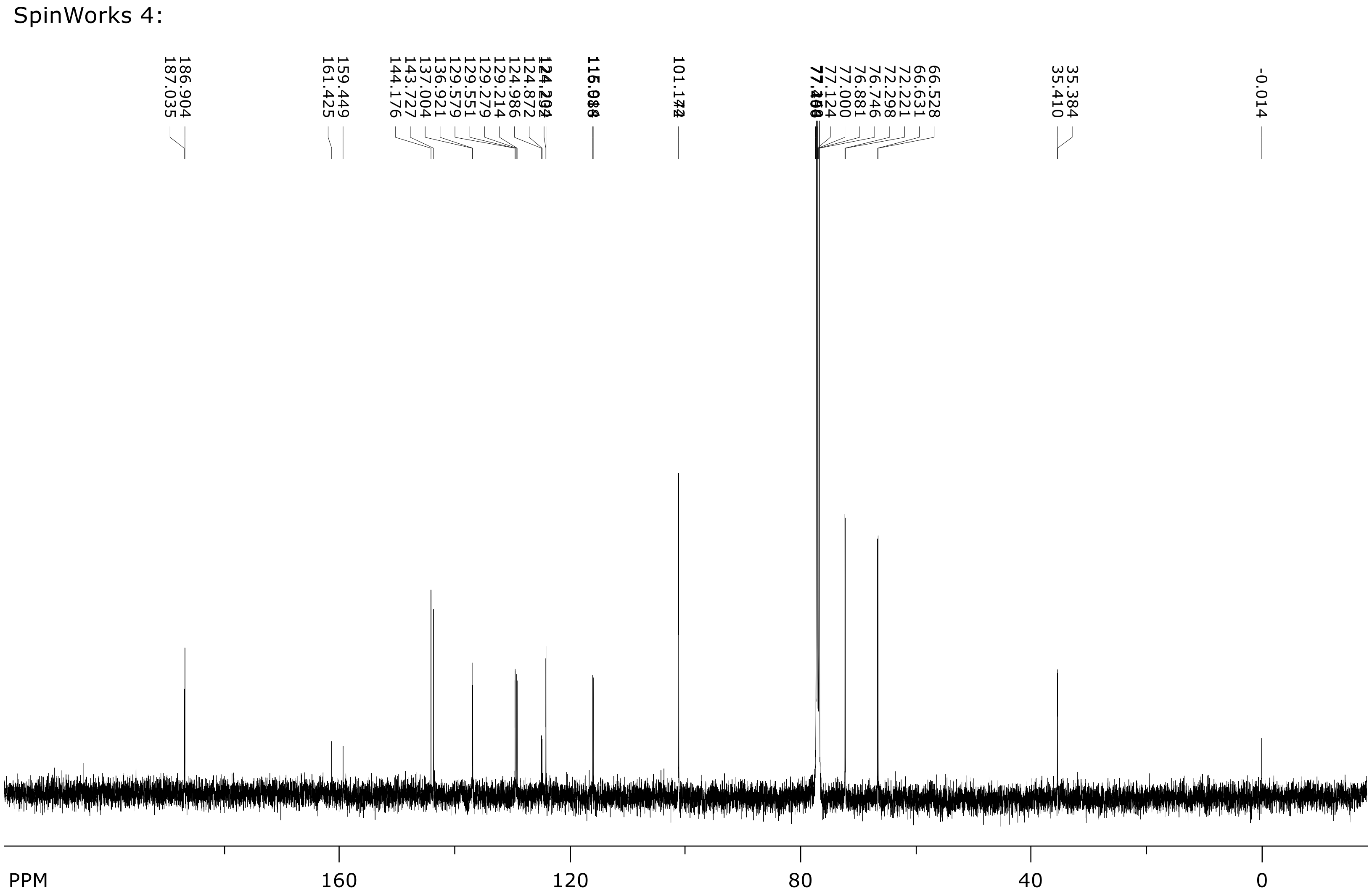

^{1}H NMR (500 MHz, $CDCl_3$) for **5m**

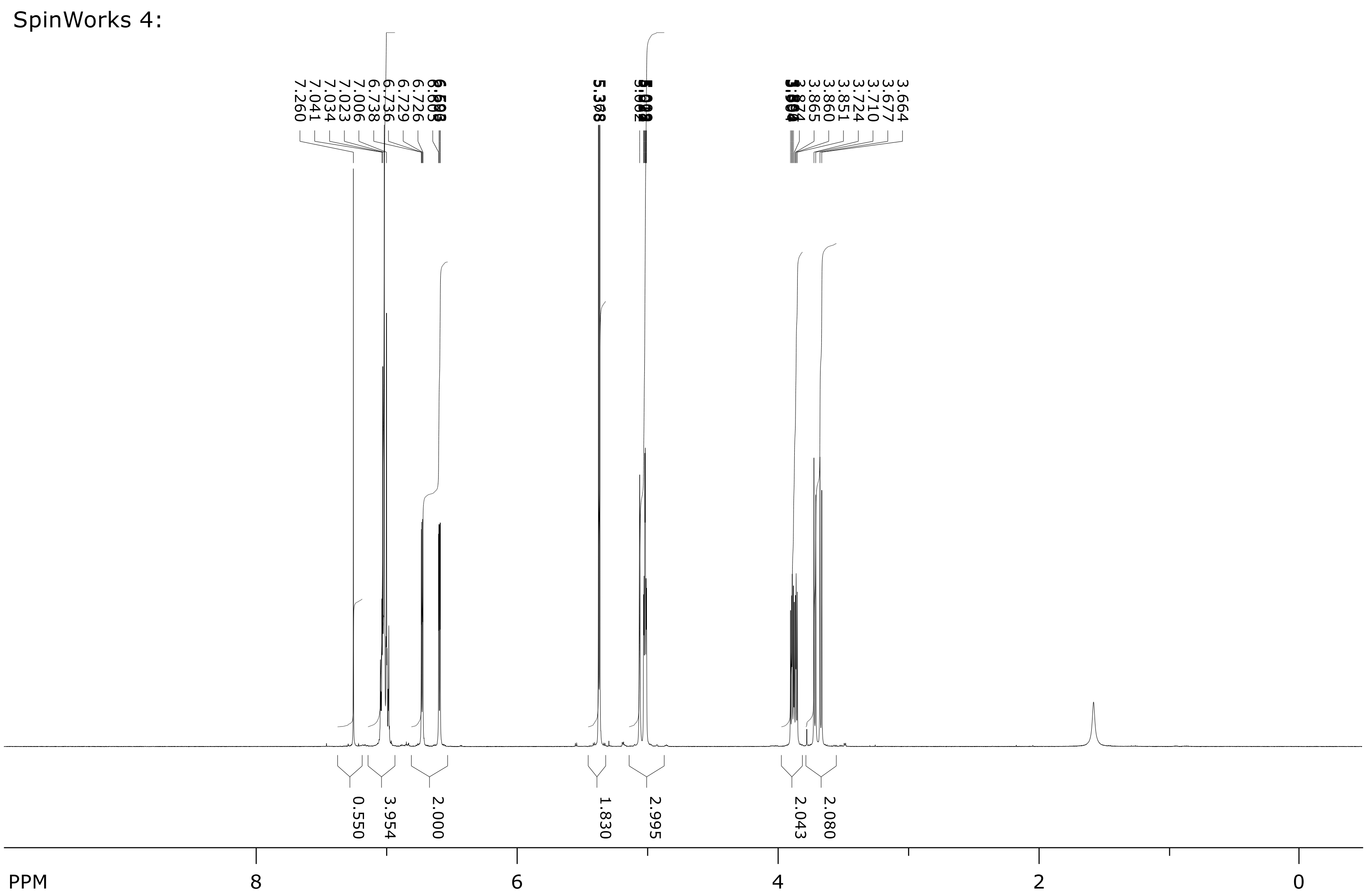

^{13}C NMR (125 MHz, $CDCl_3$) for **5m**

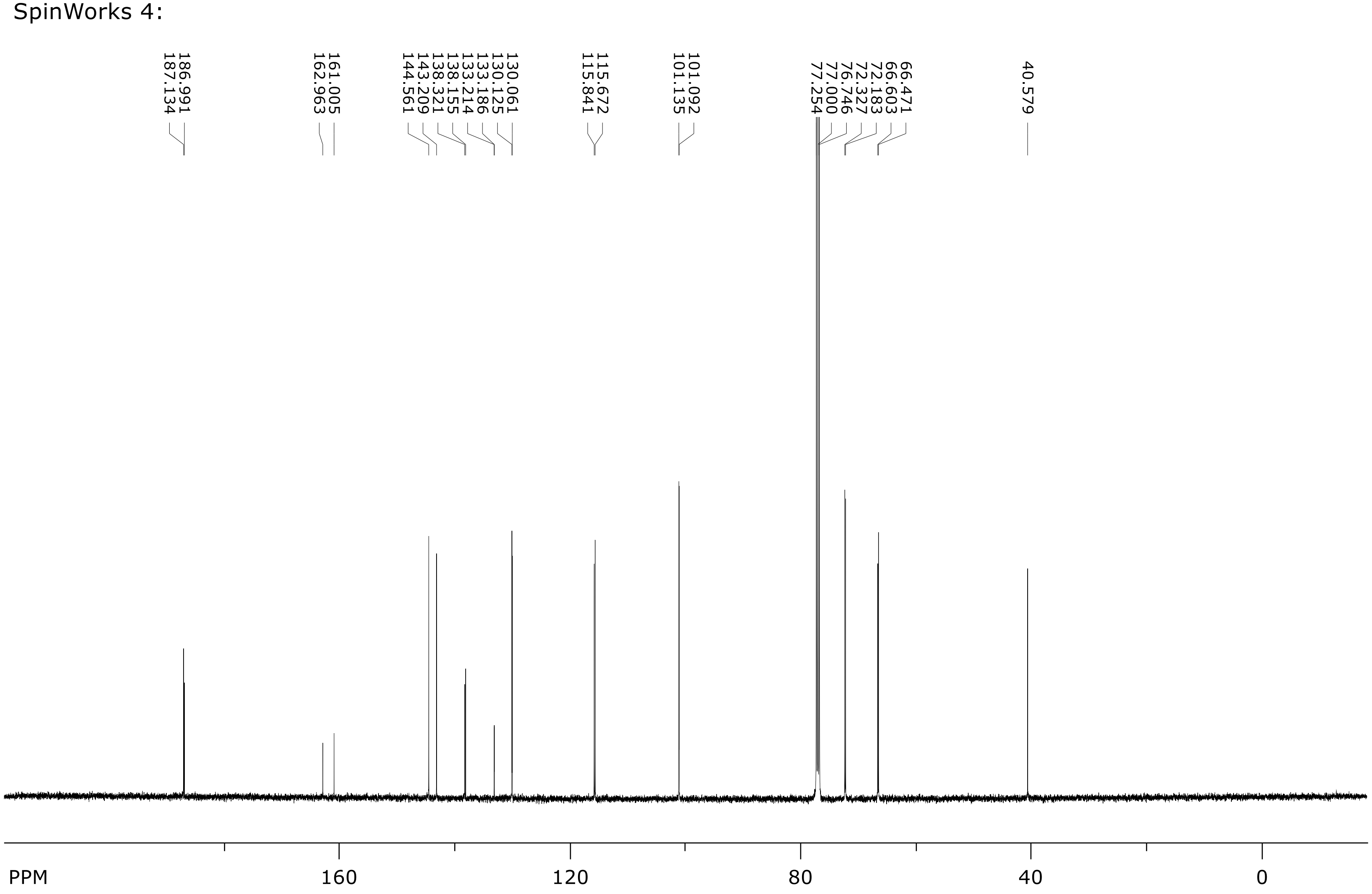

^{1}H NMR (500 MHz, $CDCl_3$) for **6**

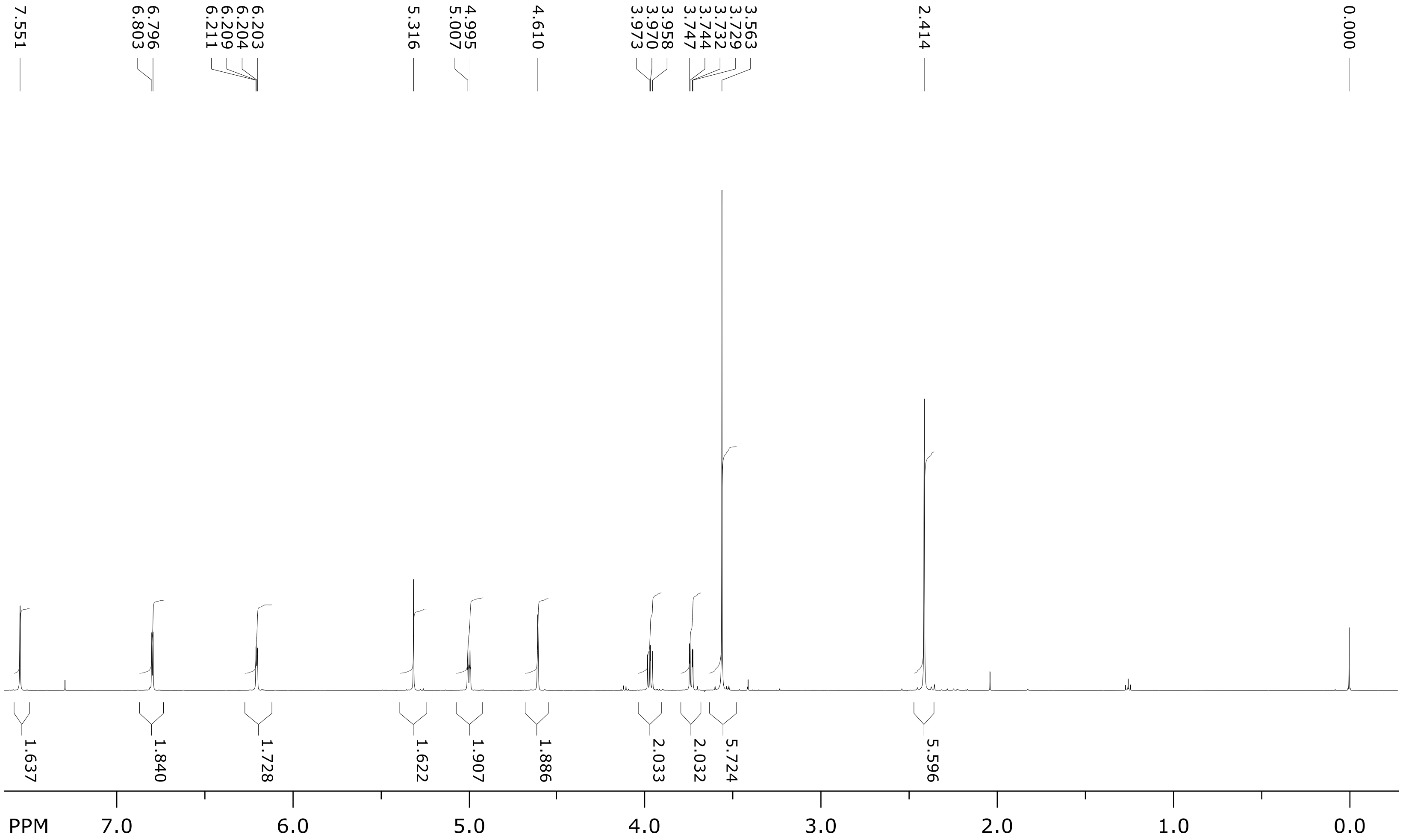

^{13}C NMR (125 MHz, $CDCl_3$) for **6**

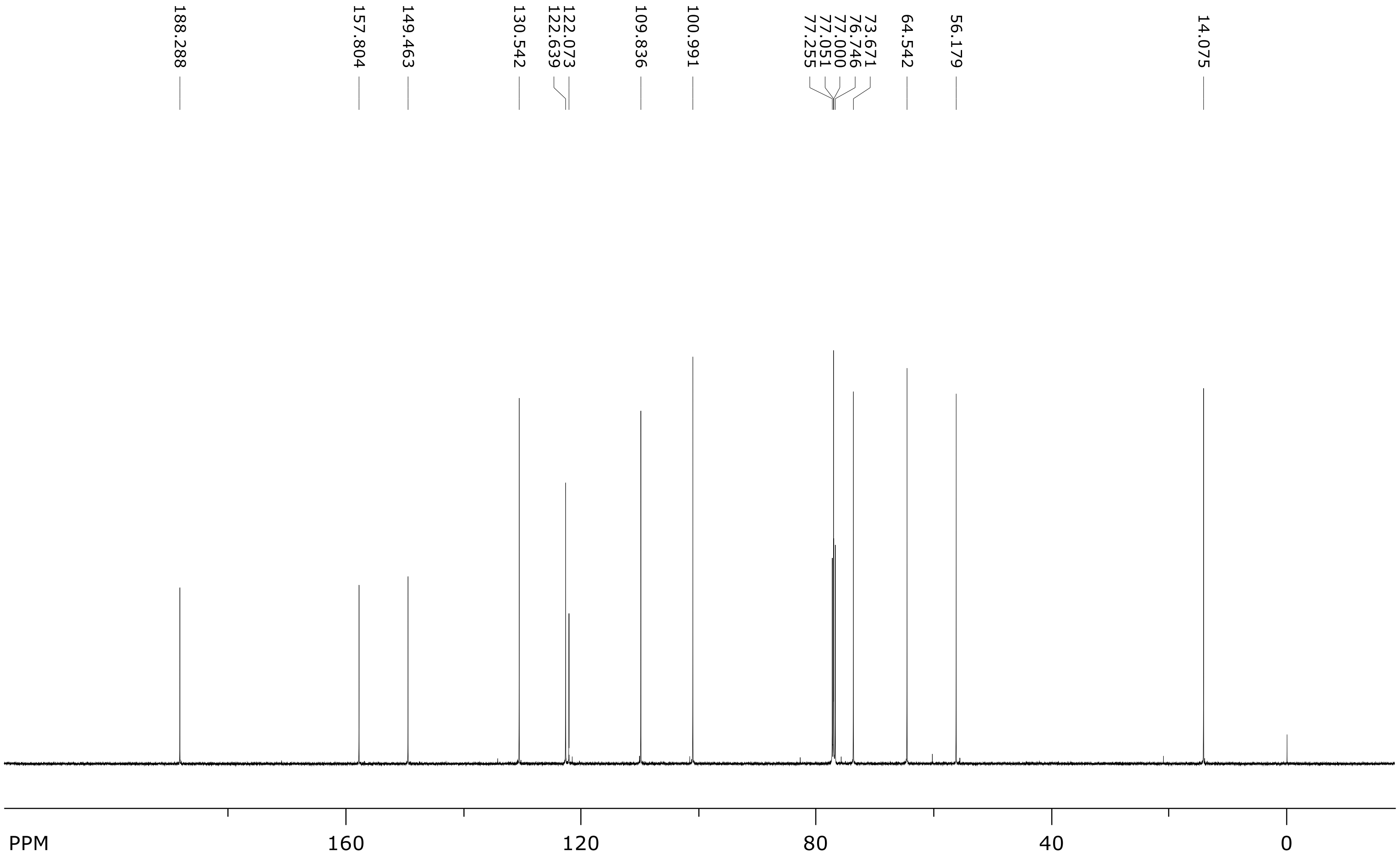

^{1}H NMR (500 MHz, $CDCl_3$) for **7**

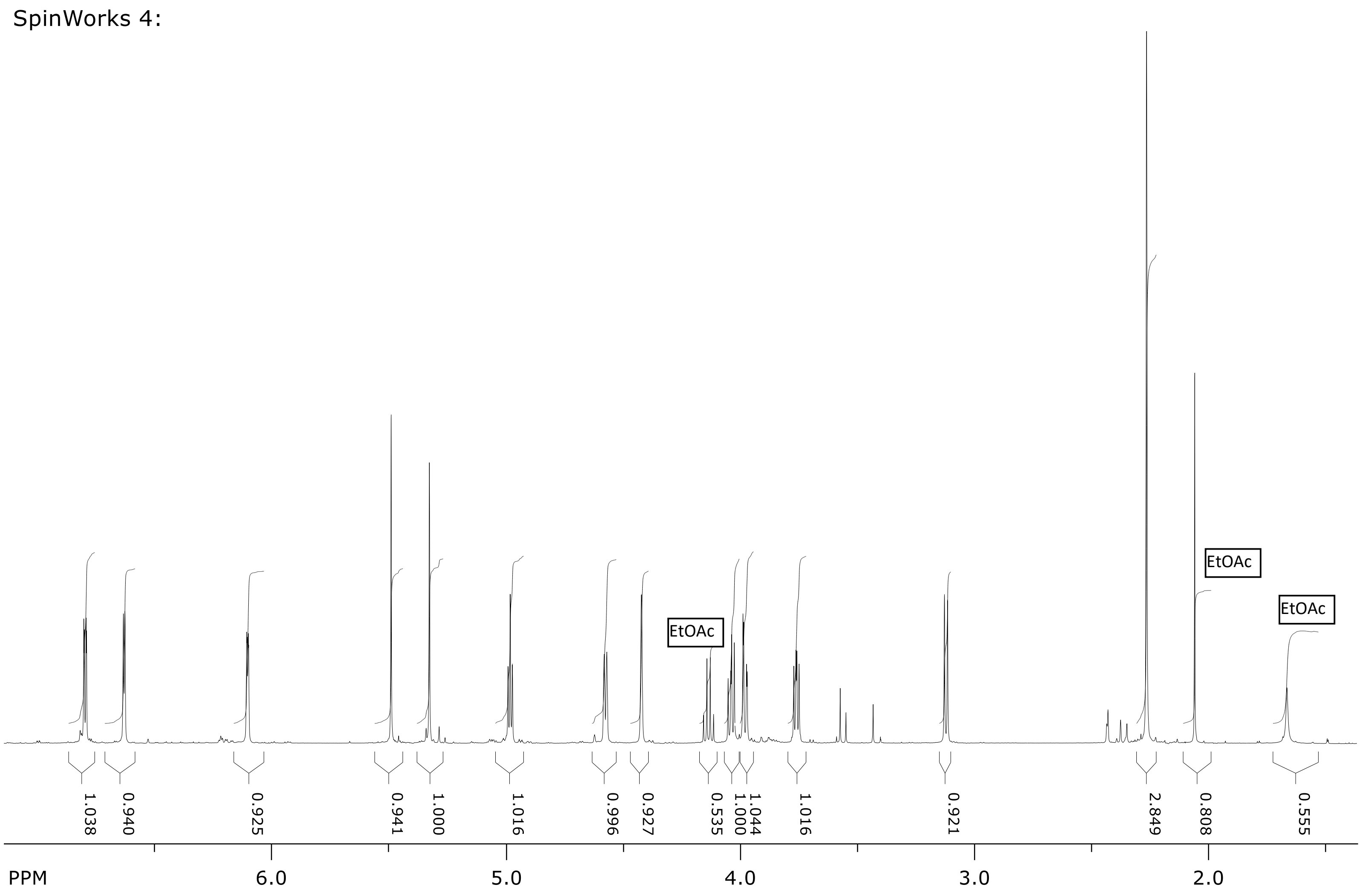

^{13}C NMR (125 MHz, $CDCl_3$) for **7**

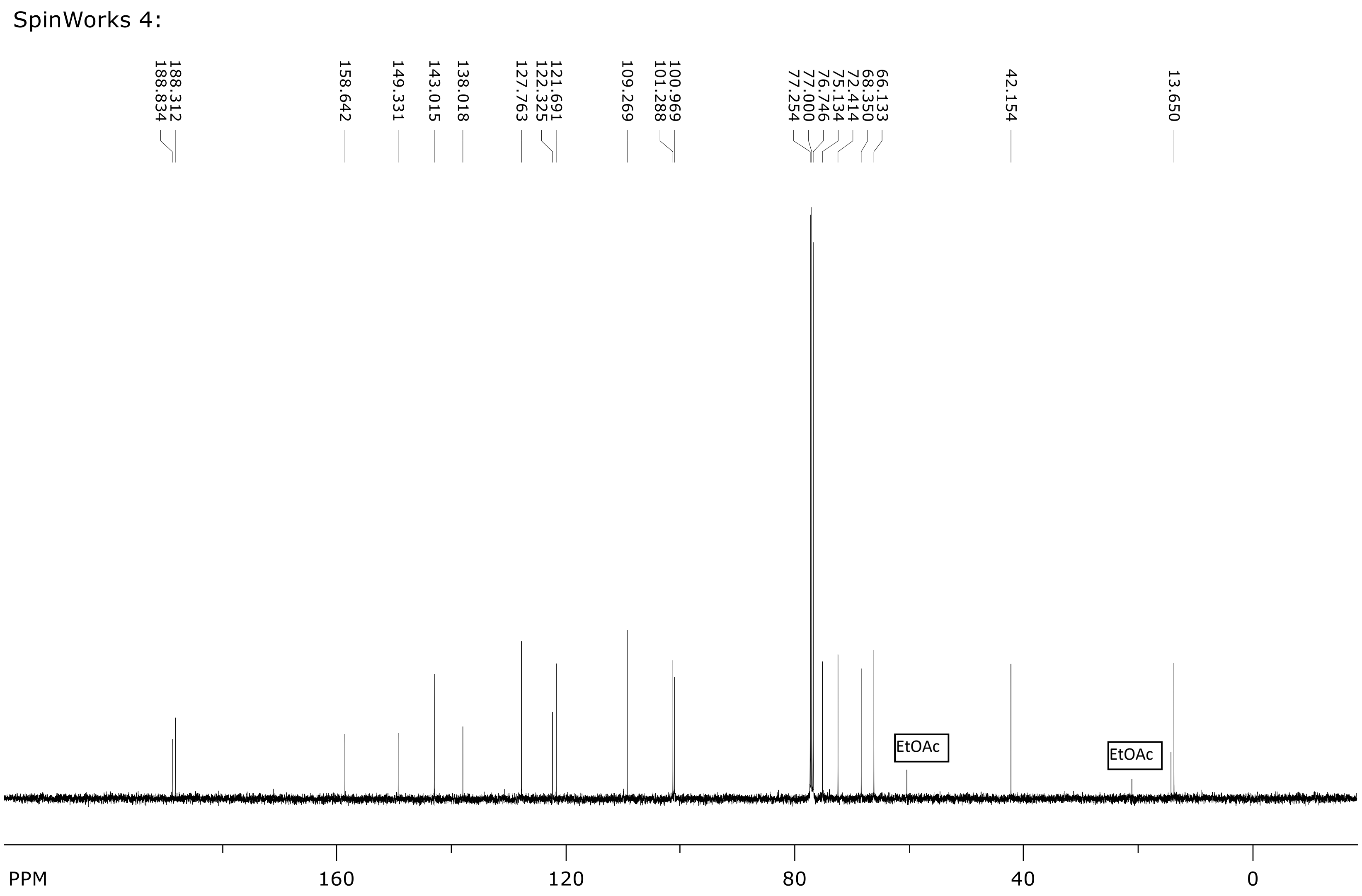

COSY spectrum for **7** (500 MHz, $CDCl_3$) with assignments

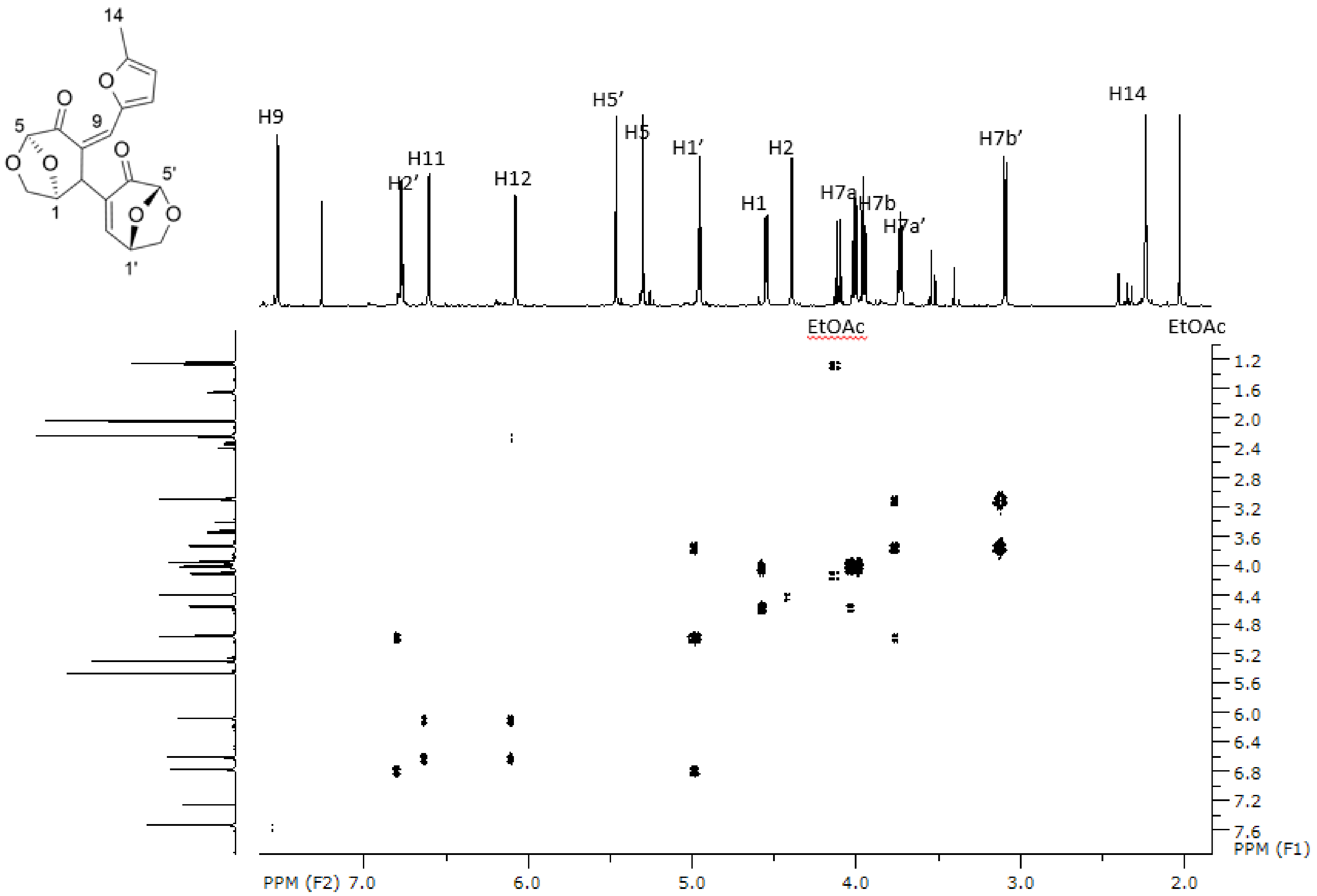

file: ...Greatrex 050321\BWG\BWG0614B\5\ser
expt: <cosygpmfphpp>
transmitter freq: 500.162173 MHz
time domain size: 2048 by 256 points
width (F2): 4504.50 Hz = 9.0061 ppm = 2.1995 Hz/pt
number of scans: 4

F2: freq. of 0 ppm: 500.1600000 MHz
processed size: 1024 complex points
window function: Sine Squared
shift: 90.0 degrees

F1: freq. of 0 ppm: 500.1600000 MHz
processed size: 1024 complex points
window function: Sine Squared
shift: 90.0 degrees

HMBC spectrum of **7** showing diagnostic crosspeak between the C7 methylene and C2

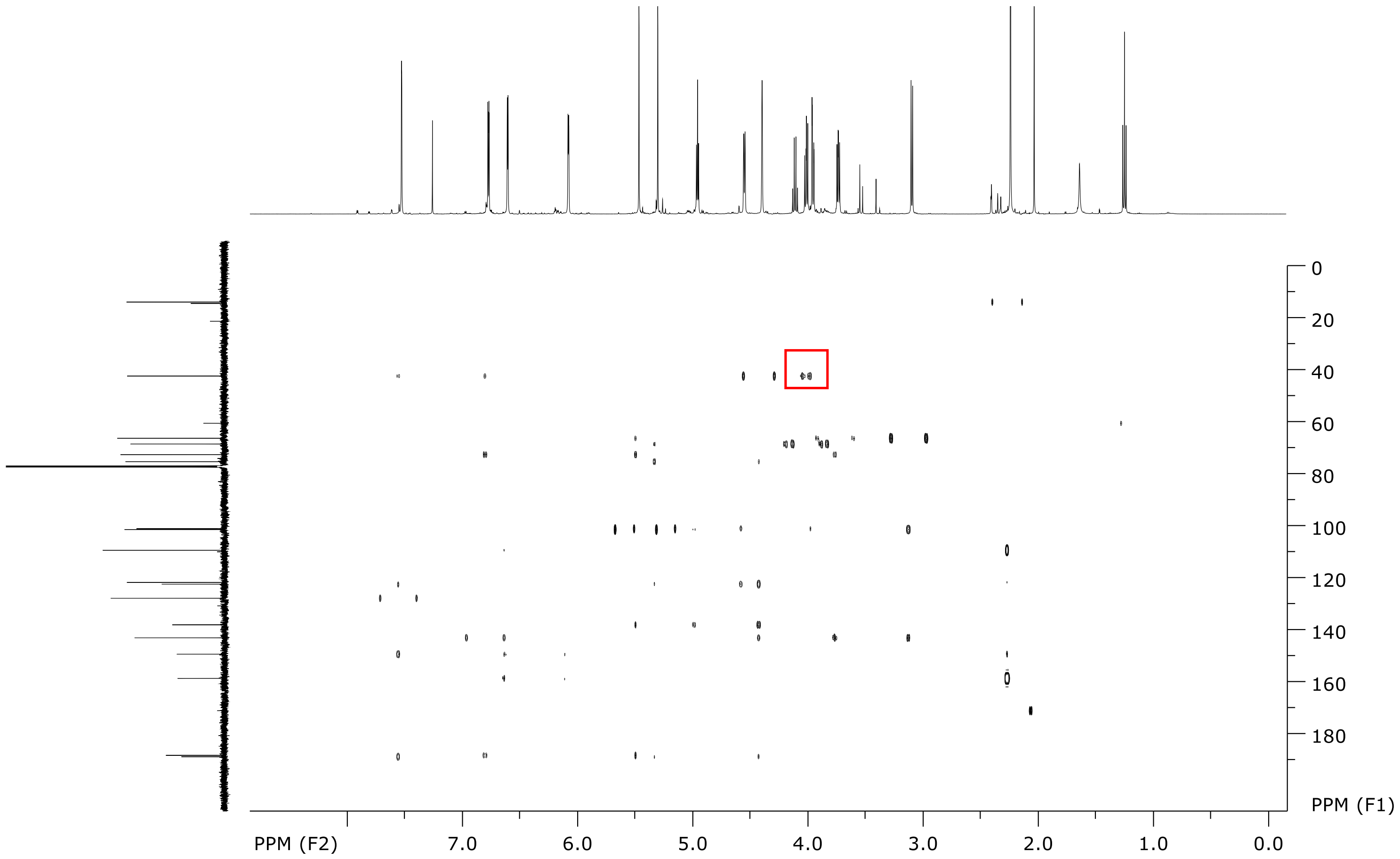

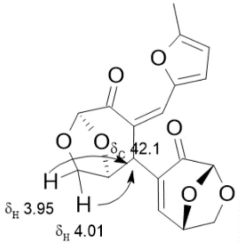

file: ...Greatrex 050321\BWG\BWG0614B\6\ser
expt: <hmbcgpndqf>
transmitter freq: 500.162173 MHz
time domain size: 4096 by 128 points
width (F2): 4504.50 Hz = 9.0061 ppm = 1.0997 Hz/pt
number of scans: 8

F2: freq. of 0 ppm: 500.1600000 MHz
processed size: 4096 complex points
window function: Sine
shift: 0.0 degrees

F1: freq. of 0 ppm: 125.7653320 MHz
processed size: 1024 complex points
window function: Sine
shift: 0.0 degrees

^{1}H NMR (500 MHz, $CDCl_3$) for **14a**

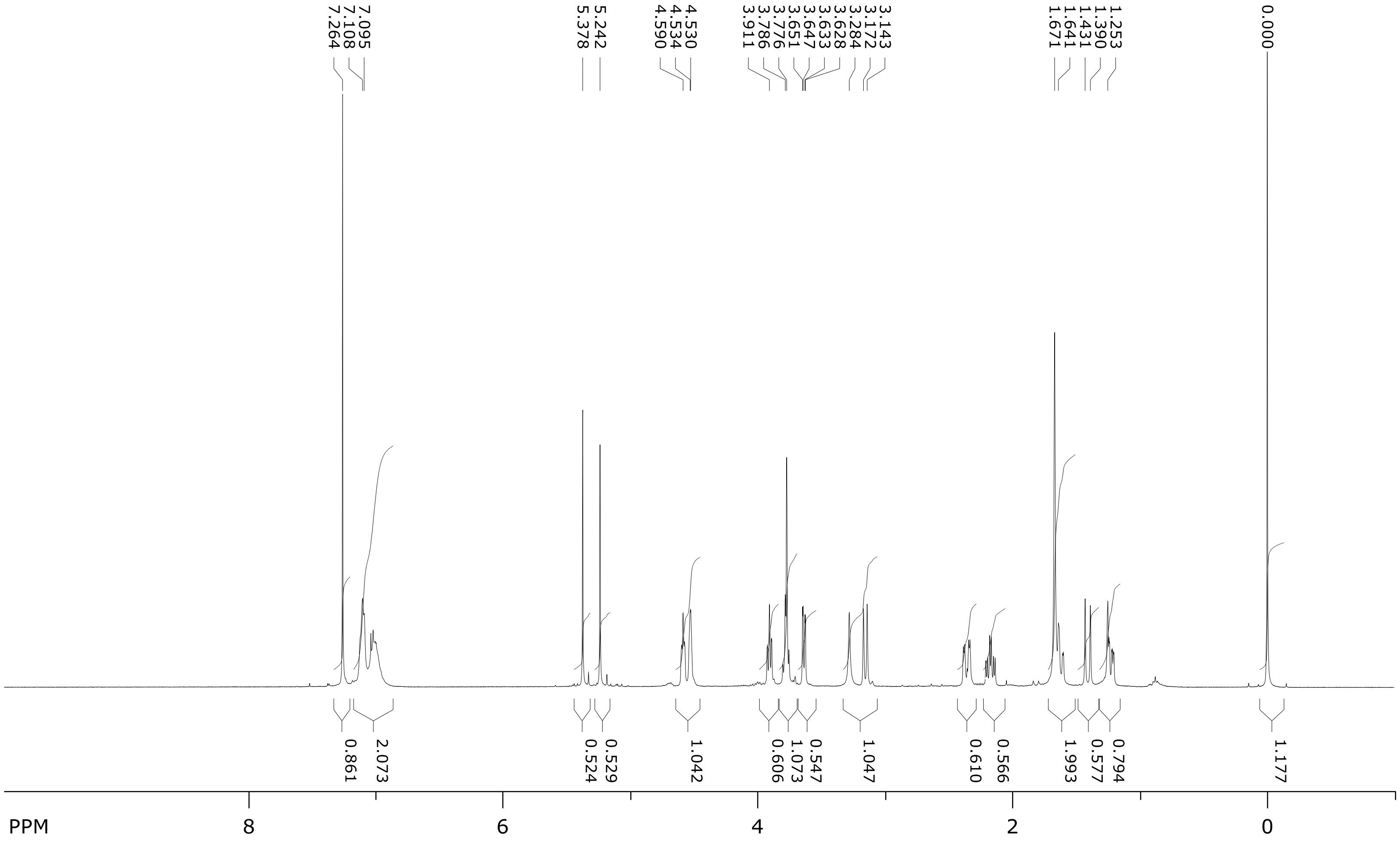

^{13}C NMR (125 MHz, $CDCl_3$) for **14a**

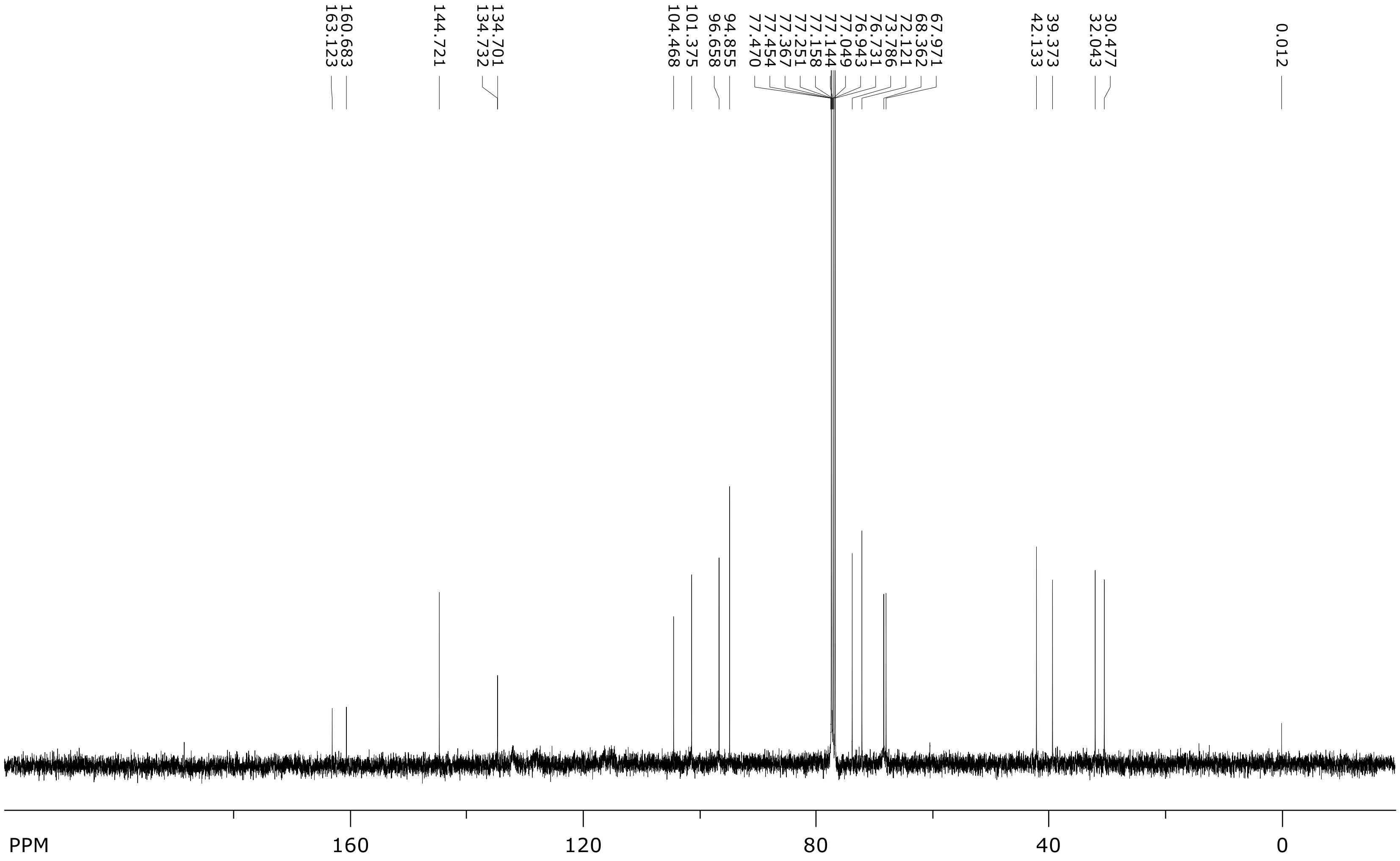

^{1}H NMR (500 MHz, $CDCl_3$) for **14b**

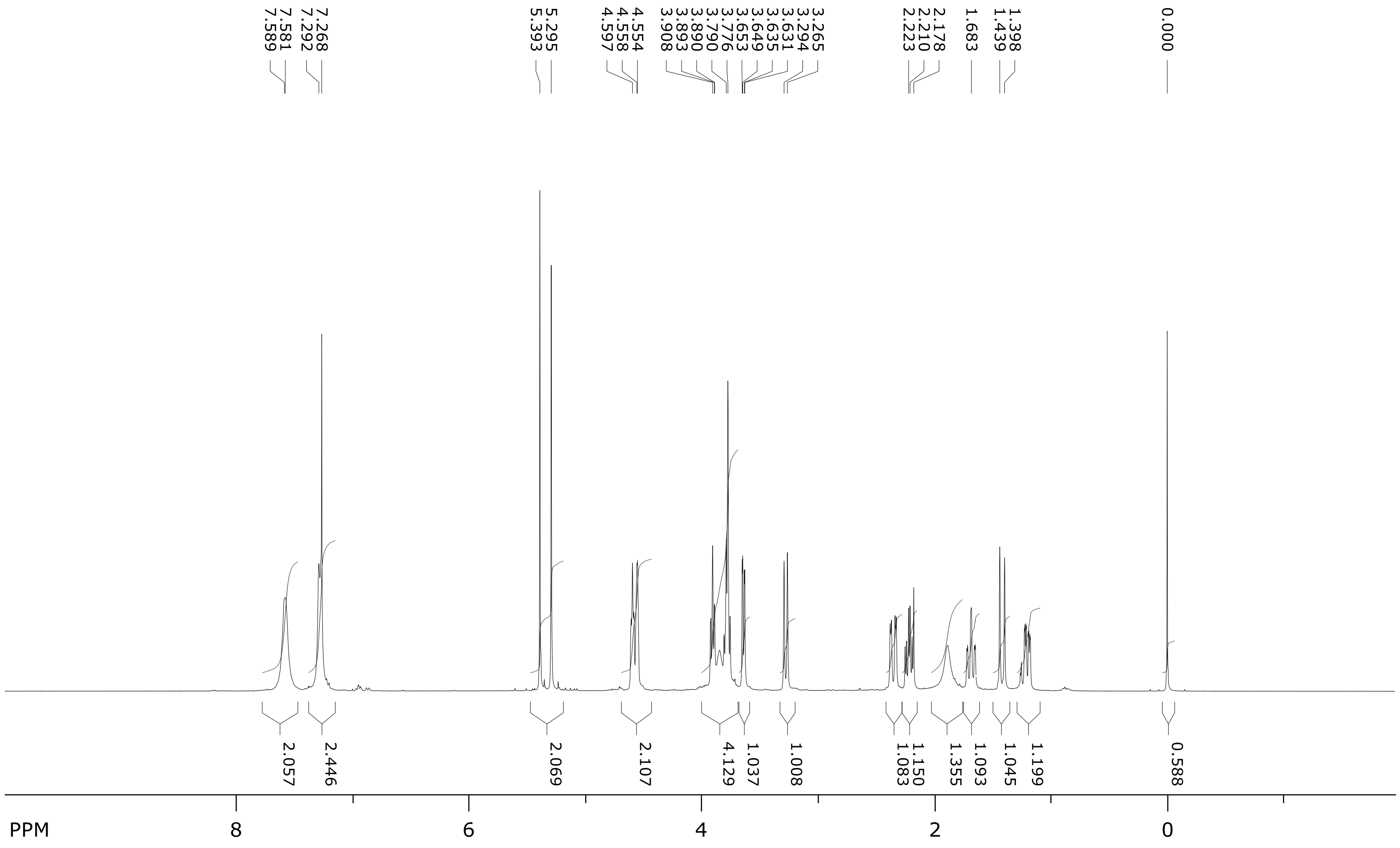

^{13}C NMR (125 MHz, $CDCl_3$) for **14b**

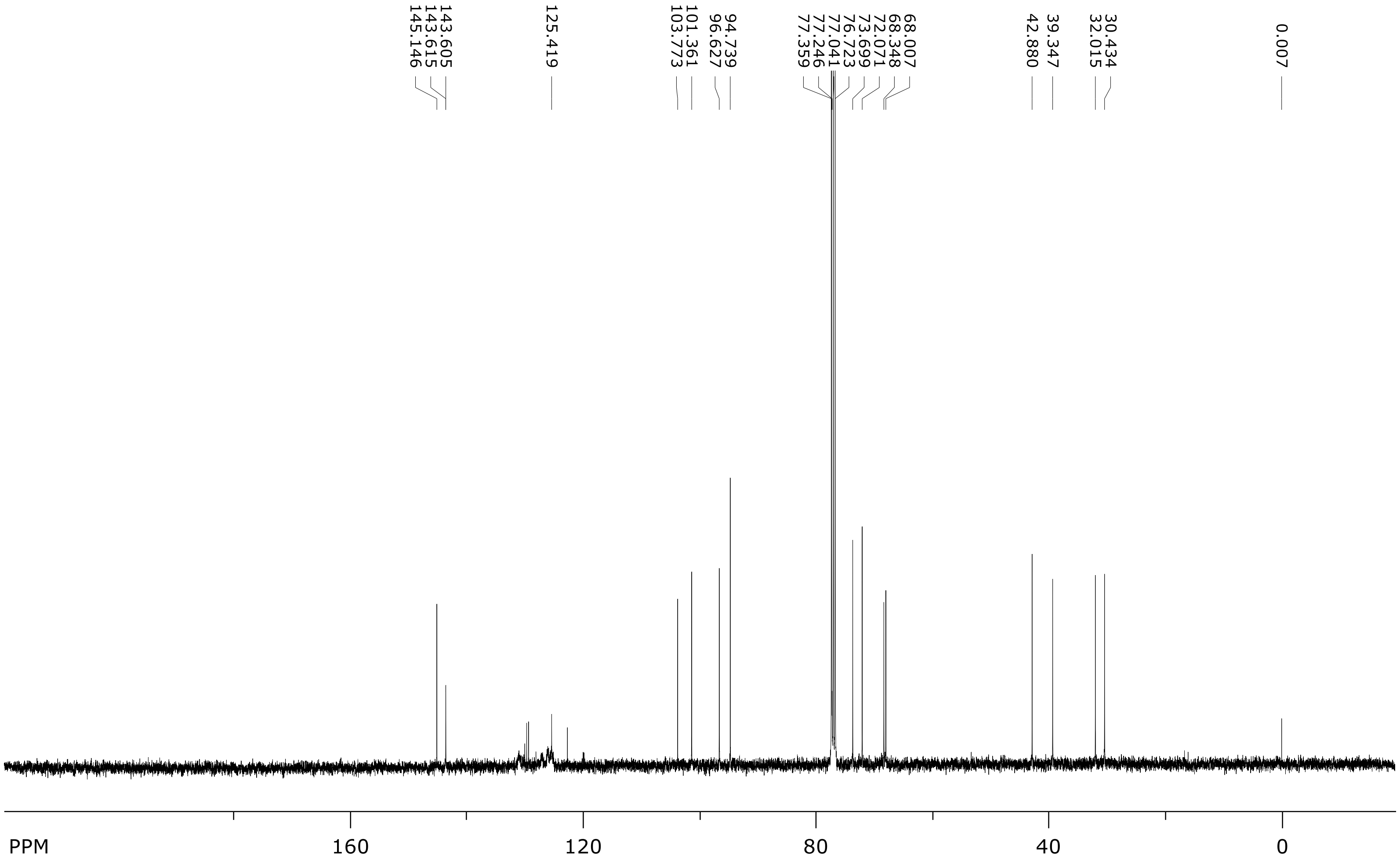

Supplement: File 1 — Experimental details for all compounds including 1H and 13C NMR spectra. [file Beilstein_J_Org_Chem-18-1457-s001.pdf]
