# Supplementary figures and images for: Oxa-Michael-initiated cascade reactions of levoglucosenone
Source: Beilstein J Org Chem. 2022 Oct 13;18:1457–62. doi: 10.3762/bjoc.18.151 (PMC9577383; doi:10.3762/bjoc.18.151)

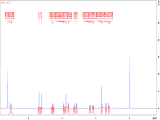

Supplement: File 2 — 1H and 13C FID data and other spectra for new compounds. [file Beilstein_J_Org_Chem-18-1457-s002.zip › supporting information2/spectra files for new compounds - minimal/data for 14a/50/pdata/1/thumb.png]

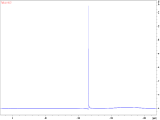

Supplement: File 2 — 1H and 13C FID data and other spectra for new compounds. [file Beilstein_J_Org_Chem-18-1457-s002.zip › supporting information2/spectra files for new compounds - minimal/data for 14a/61/pdata/1/thumb.png]

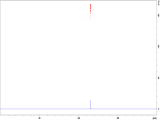

Supplement: File 2 — 1H and 13C FID data and other spectra for new compounds. [file Beilstein_J_Org_Chem-18-1457-s002.zip › supporting information2/spectra files for new compounds - minimal/data for 14a/61/pdata/2/thumb.png]

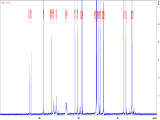

Supplement: File 2 — 1H and 13C FID data and other spectra for new compounds. [file Beilstein_J_Org_Chem-18-1457-s002.zip › supporting information2/spectra files for new compounds - minimal/data for 14a/62/pdata/1/thumb.png]

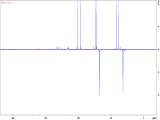

Supplement: File 2 — 1H and 13C FID data and other spectra for new compounds. [file Beilstein_J_Org_Chem-18-1457-s002.zip › supporting information2/spectra files for new compounds - minimal/data for 14a/63/pdata/1/thumb.png]

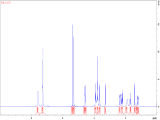

Supplement: File 2 — 1H and 13C FID data and other spectra for new compounds. [file Beilstein_J_Org_Chem-18-1457-s002.zip › supporting information2/spectra files for new compounds - minimal/data for 14b/30/pdata/1/thumb.png]

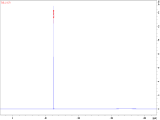

Supplement: File 2 — 1H and 13C FID data and other spectra for new compounds. [file Beilstein_J_Org_Chem-18-1457-s002.zip › supporting information2/spectra files for new compounds - minimal/data for 14b/31/pdata/1/thumb.png]

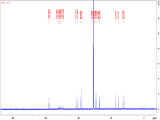

Supplement: File 2 — 1H and 13C FID data and other spectra for new compounds. [file Beilstein_J_Org_Chem-18-1457-s002.zip › supporting information2/spectra files for new compounds - minimal/data for 14b/32/pdata/1/thumb.png]
