# Supplementary material for: Oxa-Michael-initiated cascade reactions of levoglucosenone
Source: Beilstein J Org Chem. 2022 Oct 13;18:1457–62. doi: 10.3762/bjoc.18.151 (PMC9577383; doi:10.3762/bjoc.18.151)
Supplement: File 2 — 1H and 13C FID data and other spectra for new compounds. [file Beilstein_J_Org_Chem-18-1457-s002.zip › supporting information2/spectra files for new compounds - minimal/data for 5a/ESIMS 5a.pdf]

MS Spectrum

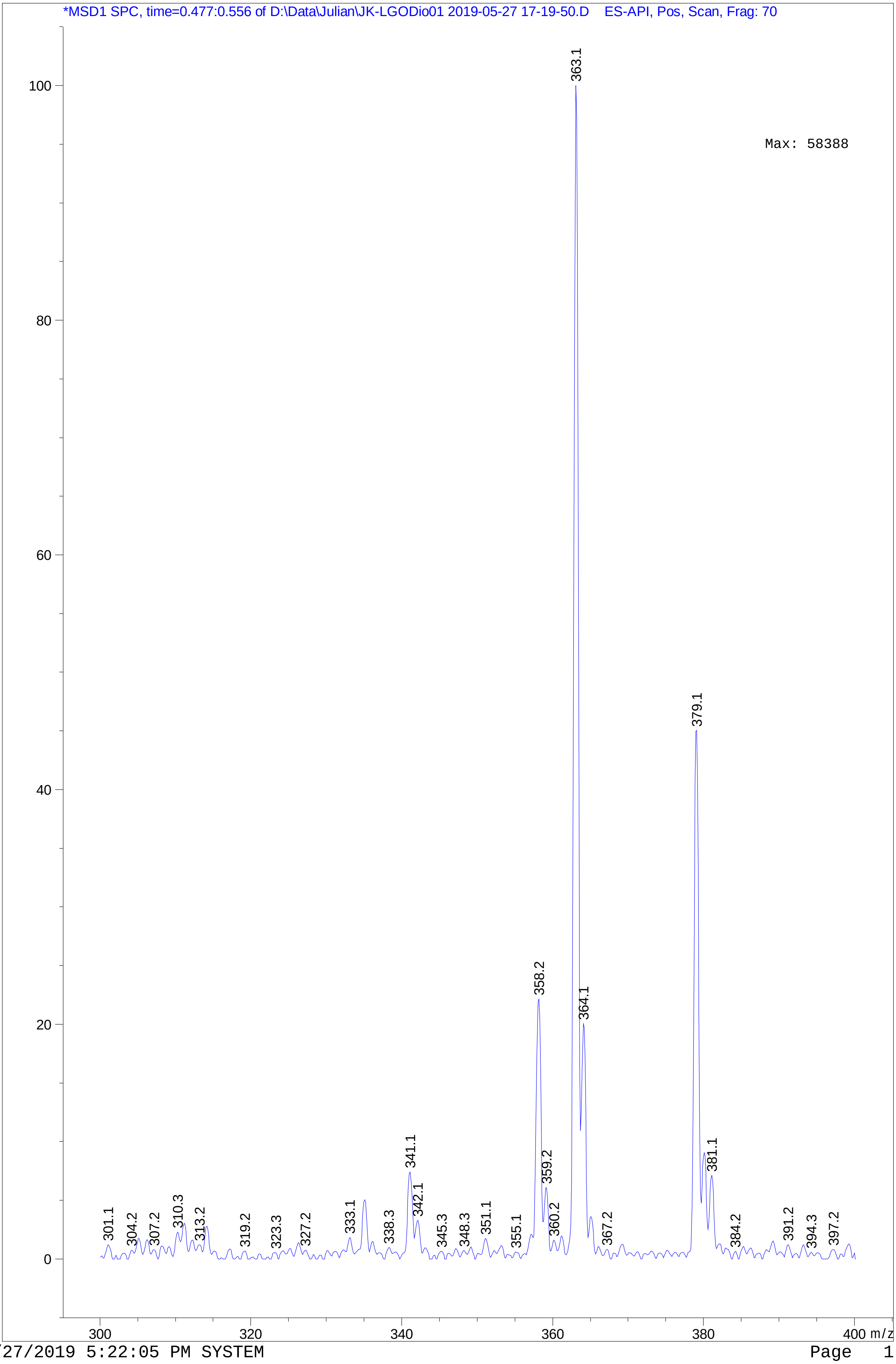
*MSD1 SPC, time=0.477:0.556 of D:\Data\Julian\JK-LGODio01 2019-05-27 17-19-50.D ES-API, Pos, Scan, Frag: 70
Max: 58388
363.1
379.1
358.2
364.1
341.1
381.1
359.2
342.1
301.1
304.2
307.2
310.3
313.2
319.2
323.3
327.2
333.1
338.3
345.3
348.3
351.1
355.1
360.2
367.2
384.2
391.2
394.3
397.2
100
80
60
40
20
0
300
320
340
360
380
400
m/z
