# Supplementary material for: Oxa-Michael-initiated cascade reactions of levoglucosenone
Source: Beilstein J Org Chem. 2022 Oct 13;18:1457–62. doi: 10.3762/bjoc.18.151 (PMC9577383; doi:10.3762/bjoc.18.151)
Supplement: File 2 — 1H and 13C FID data and other spectra for new compounds. [file Beilstein_J_Org_Chem-18-1457-s002.zip › supporting information2/spectra files for new compounds - minimal/data for 5a/NMR data for 5a/13C/pdata/1/JK-LGODi01-Hughcheck_4_1.pdf]

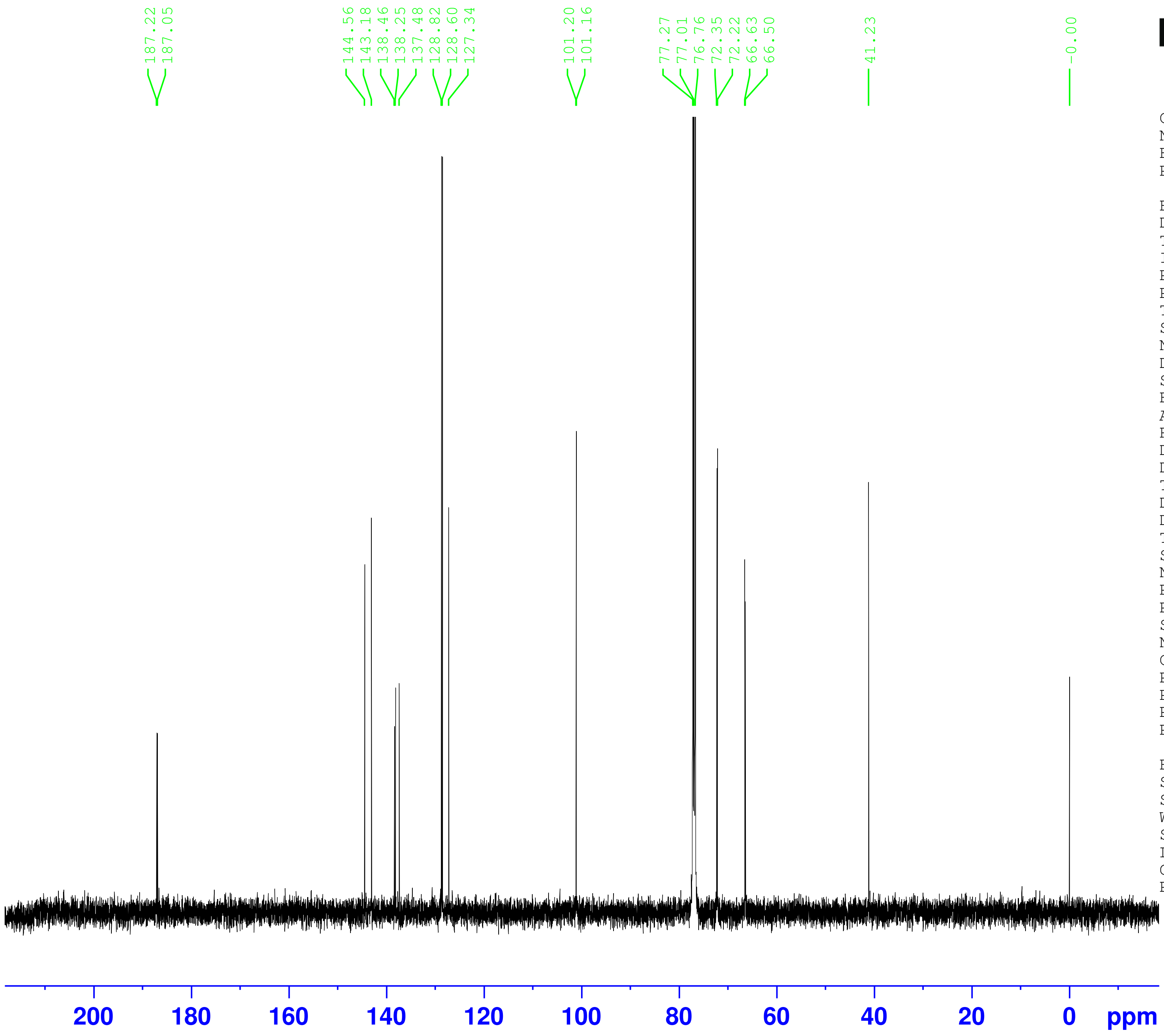

BRUKER

```
Current Data Parameters
NAME      JK-LGODi01-Hughcheck
EXPNO                        4
PROCNO                       1

F2 - Acquisition Parameters
Date_                 20180626
Time                     21.28 h
INSTRUM                  spect
PROBHD    Z119470_0196 (
PULPROG                zgpg30
TD                       65536
SOLVENT                  CDCl3
NS                        3000
DS                           4
SWH                  29761.904 Hz
FIDRES                0.908261 Hz
AQ                   1.1010048 sec
RG                      191.36
DW                      16.800 usec
DE                        6.50 usec
TE                       295.0 K
D1                  2.00000000 sec
D11                 0.03000000 sec
TD0                          1
SFO1               125.7779086 MHz
NUC1                       13C
P1                       10.00 usec
PLW1               78.00000000 W
SFO2               500.1620006 MHz
NUC2                        1H
CPDPRG[2               waltz16
PCPD2                    80.00 usec
PLW2               22.00000000 W
PLW12               0.35764000 W
PLW13               0.17989001 W

F2 - Processing parameters
SI                       32768
SF                 125.7653336 MHz
WDW                         EM
SSB       0
LB                        1.00 Hz
GB        0
PC                        1.40
```
