# Supplementary material for: Oxa-Michael-initiated cascade reactions of levoglucosenone
Source: Beilstein J Org Chem. 2022 Oct 13;18:1457–62. doi: 10.3762/bjoc.18.151 (PMC9577383; doi:10.3762/bjoc.18.151)
Supplement: File 2 — 1H and 13C FID data and other spectra for new compounds. [file Beilstein_J_Org_Chem-18-1457-s002.zip › supporting information2/spectra files for new compounds - minimal/data for 5a/NMR data for 5a/COSY/pdata/1/JK-LGODi01-Hughcheck_3_1.pdf]

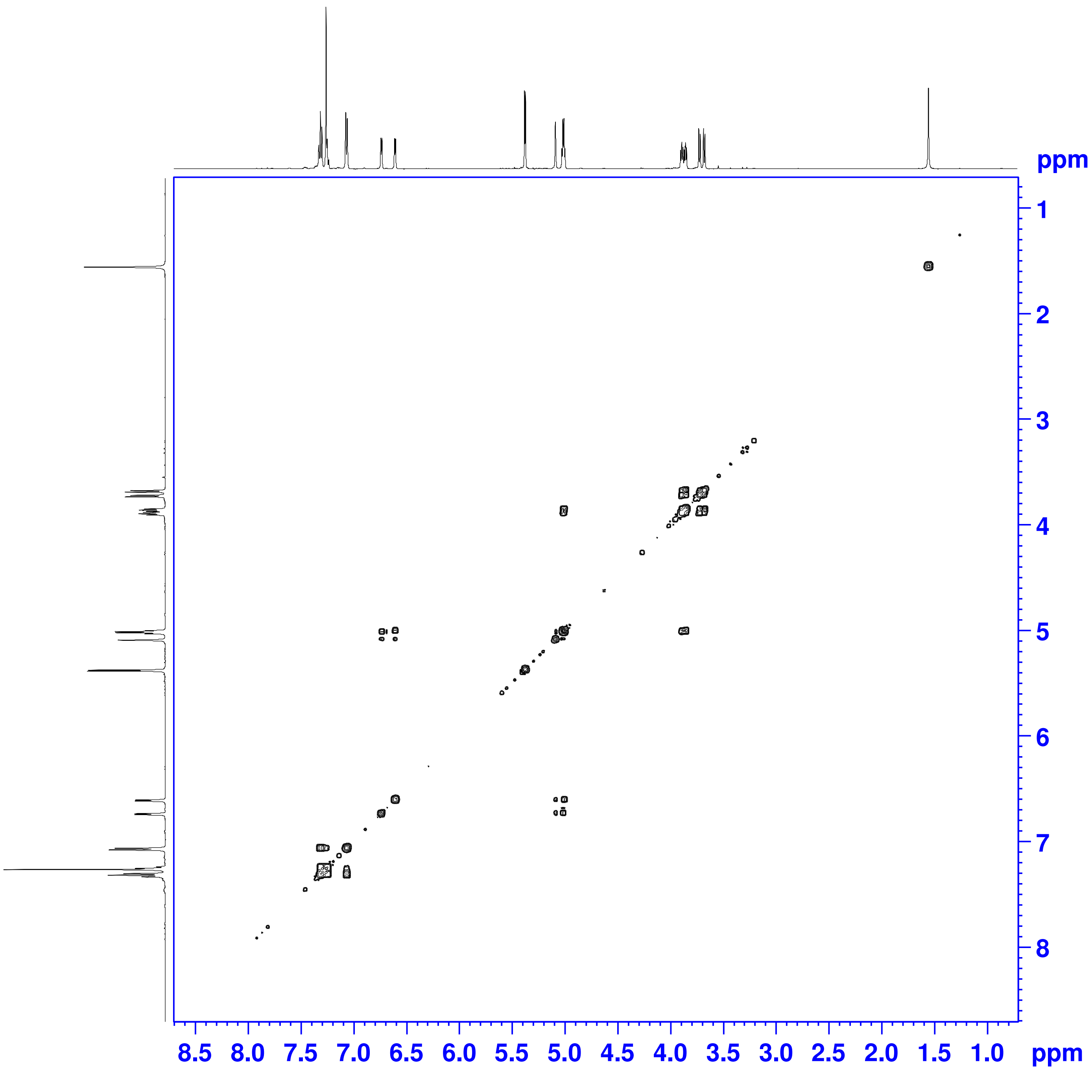

BRUKER

```
Current Data Parameters
NAME      JK-LGODi01-Hughcheck
EXPNO                        3
PROCNO                       1

F2 - Acquisition Parameters
Date_              20180626
Time                  18.10 h
INSTRUM               spect
PROBHD   Z119470_0196 (
PULPROG         cosygpppqf
TD                    2048
SOLVENT              CDCl3
NS                       1
DS                      16
SWH               4000.000 Hz
FIDRES            3.906250 Hz
AQ               0.2560000 sec
RG                   62.39
DW                 125.000 usec
DE                    6.50 usec
TE                   295.0 K
D0              0.00000300 sec
D1              1.90128601 sec
D11             0.03000000 sec
D12             0.00002000 sec
D13             0.00000400 sec
D16             0.00020000 sec
IN0             0.00025000 sec
TDav                     1
SFO1           500.1623659 MHz
NUC1                    1H
P0                   10.20 usec
P1                   10.20 usec
P17                2500.00 usec
PLW1           22.00000000 W
PLW10           2.54320002 W
GPNAM[1]        SMSQ10.100
GPZ1                 10.00 %
P16                1000.00 usec

F1 - Acquisition parameters
TD                     128
SFO1              500.1624 MHz
FIDRES           62.500000 Hz
SW                   7.997 ppm
FnMODE                  QF

F2 - Processing parameters
SI                    1024
SF             500.1600113 MHz
WDW                  QSINE
SSB      0
LB       0  Hz
GB       0
PC                    1.40

F1 - Processing parameters
SI                    1024
MC2                     QF
SF             500.1600113 MHz
WDW                  QSINE
SSB      0
LB       0  Hz
GB       0
```
