# Supplementary material for: Oxa-Michael-initiated cascade reactions of levoglucosenone
Source: Beilstein J Org Chem. 2022 Oct 13;18:1457–62. doi: 10.3762/bjoc.18.151 (PMC9577383; doi:10.3762/bjoc.18.151)
Supplement: File 2 — 1H and 13C FID data and other spectra for new compounds. [file Beilstein_J_Org_Chem-18-1457-s002.zip › supporting information2/spectra files for new compounds - minimal/data for 5a/NMR data for 5a/HMBC/pdata/1/JK-LGODi01-Hughcheck_7_1.pdf]

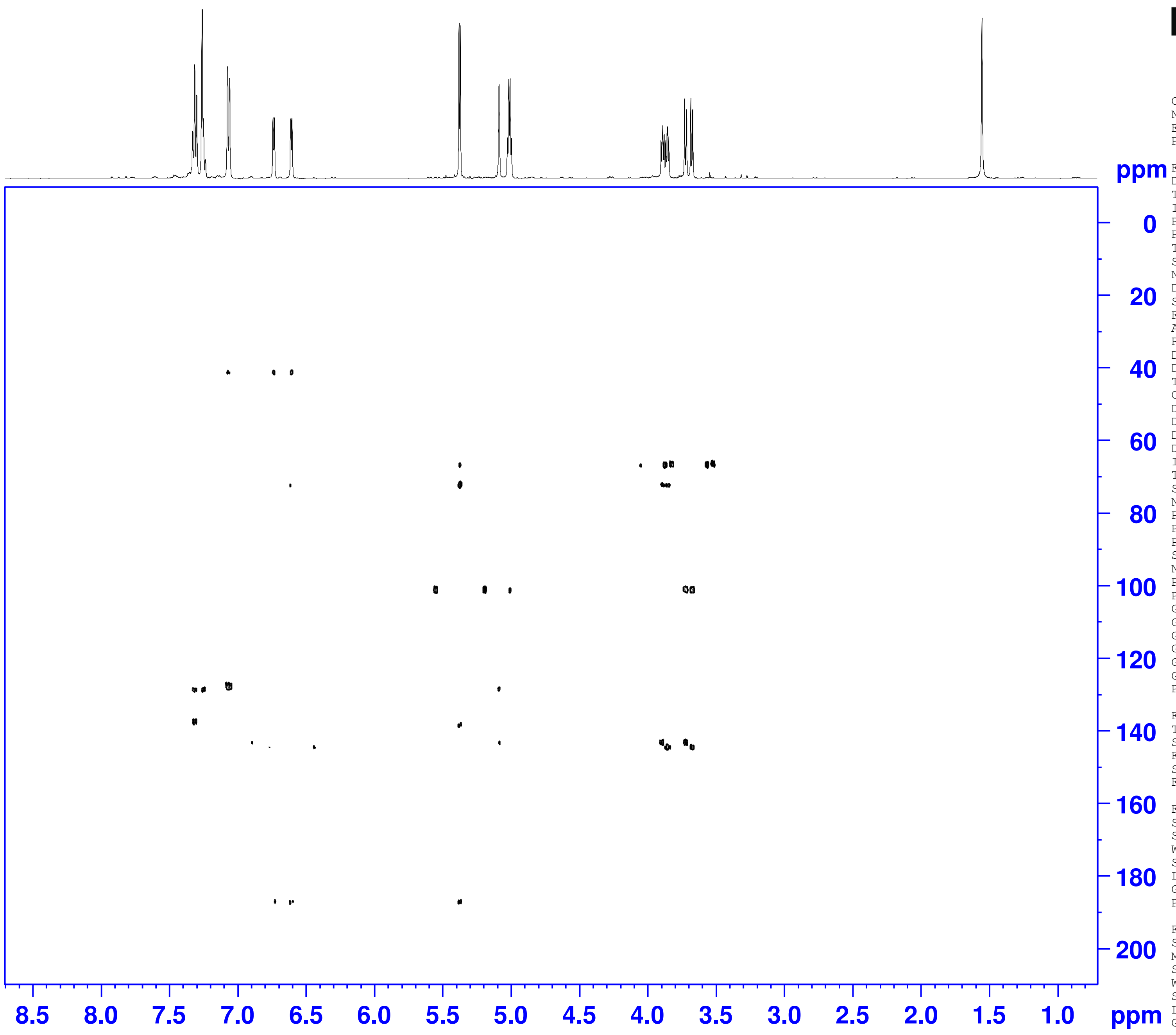

BRUKER

Current Data Parameters

NAME	JK-LGODi01-Hughcheck
EXPNO	7
PROCNO	1

F2 - Acquisition Parameters

Date_	20180626
Time	22.55 h
INSTRUM	spect
PROBHD	Z119470_0196 (
PULPROG	hmbcgpndqf
TD	4096
SOLVENT	CDCl3
NS	8
DS	16
SWH	4000.000 Hz
FIDRES	1.953125 Hz
AQ	0.5120000 sec
RG	191.36
DW	125.000 usec
DE	6.50 usec
TE	295.0 K
CNST13	8.0000000
D0	0.00000300 sec
D1	1.30257297 sec
D6	0.06250000 sec
D16	0.00020000 sec
IN0	0.00001810 sec
TDav	1
SFO1	500.1623659 MHz
NUC1	1H
P1	10.20 usec
P2	20.40 usec
PLW1	22.00000000 W
SFO2	125.7779086 MHz
NUC2	13C
P3	10.00 usec
PLW2	78.00000000 W
GPNAM[1]	SMSQ10.100
GPZ1	50.00 %
GPNAM[2]	SMSQ10.100
GPZ2	30.00 %
GPNAM[3]	SMSQ10.100
GPZ3	40.10 %
P16	1000.00 usec

F1 - Acquisition parameters

TD	128
SFO1	125.7779 MHz
FIDRES	431.629822 Hz
SW	219.628 ppm
FnMODE	QF

F2 - Processing parameters

SI	4096
SF	500.1600113 MHz
WDW	SINE
SSB	0
LB	0 Hz
GB	0
PC	1.40

F1 - Processing parameters

SI	1024
MC2	QF
SF	125.7653320 MHz
WDW	SINE
SSB	0
LB	0 Hz
GB	0
