# Supplementary material for: Oxa-Michael-initiated cascade reactions of levoglucosenone
Source: Beilstein J Org Chem. 2022 Oct 13;18:1457–62. doi: 10.3762/bjoc.18.151 (PMC9577383; doi:10.3762/bjoc.18.151)
Supplement: File 2 — 1H and 13C FID data and other spectra for new compounds. [file Beilstein_J_Org_Chem-18-1457-s002.zip › supporting information2/spectra files for new compounds - minimal/data for 5a/NMR data for 5a/HSQC/pdata/1/JK-LGODi01-Hughcheck_5_1.pdf]

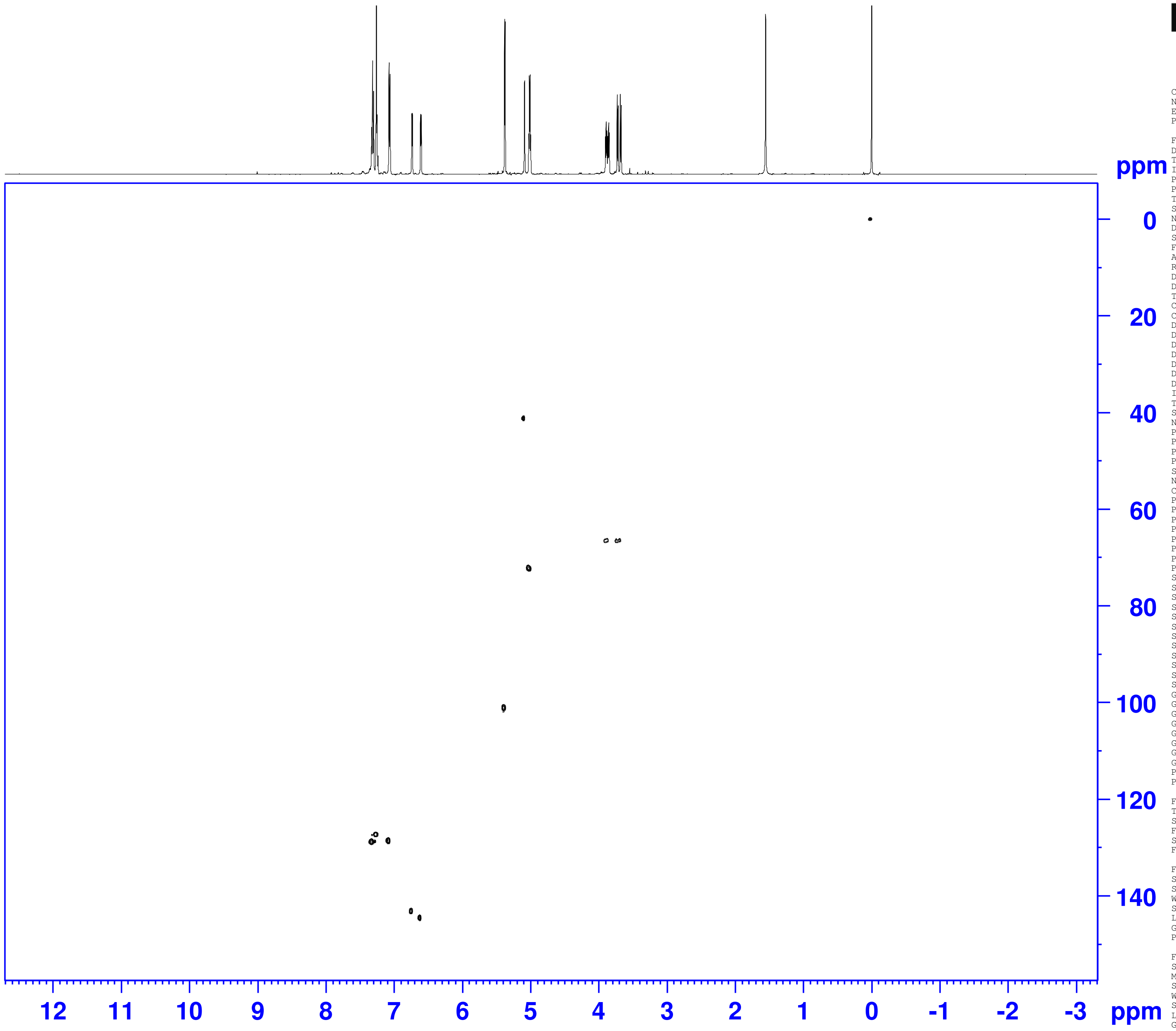


```
Current Data Parameters
NAME     JK-LGODi01-Hughcheck
EXPNO                  5
PROCNO                 1

F2 - Acquisition Parameters
Date_          20180626
Time              21.31 h
INSTRUM           spect
PROBHD   Z119470_0196 (
PULPROG  hsqcedetgpsisp2.3
TD                 2048
SOLVENT           CDCl3
NS                    4
DS                   32
SWH            8012.820 Hz
FIDRES         7.825020 Hz
AQ            0.1277952 sec
RG               191.36
DW               62.400 usec
DE                 6.50 usec
TE                295.0 K
CNST2       145.0000000
CNST17       -0.5000000
D0           0.00000300 sec
D1           2.00000000 sec
D4           0.00172414 sec
D11          0.03000000 sec
D16          0.00020000 sec
D21          0.00360000 sec
D24          0.00089000 sec
IN0          0.00002410 sec
TDav                  1
SFO1        500.1623508 MHz
NUC1                 1H
P1                10.20 usec
P2                20.40 usec
P28             1000.00 usec
PLW1        22.00000000 W
SFO2        125.7747644 MHz
NUC2                13C
CPDPRG[2           garp
P3                10.00 usec
P14              500.00 usec
P24             2000.00 usec
P31             1895.00 usec
PCPD2             70.00 usec
PLW0     0 W
PLW2        78.00000000 W
PLW12        1.59179997 W
SPNAM[3] Crp60,0.5,20.1
SPOAL3            0.500
SPOFFS3  0 Hz
SPW3        11.91800022 W
SPNAM[7]    Crp60comp.4
SPOAL7            0.500
SPOFFS7  0 Hz
SPW7        11.91800022 W
SPNAM[18  Crp60_xfilt.2
SPOAL18           0.500
SPOFFS18 0 Hz
SPW18        2.87069988 W
GPNAM[1]     SMSQ10.100
GPZ1              80.00 %
GPNAM[2]     SMSQ10.100
GPZ2              20.10 %
GPNAM[3]     SMSQ10.100
GPZ3              11.00 %
GPNAM[4]     SMSQ10.100
GPZ4              -5.00 %
P16             1000.00 usec
P19              600.00 usec

F1 - Acquisition parameters
TD                  256
SFO1           125.7748 MHz
FIDRES       162.085068 Hz
SW              164.953 ppm
FnMODE     Echo-Antiecho

F2 - Processing parameters
SI                 1024
SF          500.1600000 MHz
WDW               QSINE
SSB                   2
LB       0 Hz
GB       0
PC                 1.40

F1 - Processing parameters
SI                 1024
MC2       echo-antiecho
SF          125.7653320 MHz
WDW               QSINE
SSB                   2
LB       0 Hz
GB       0
```
