# Supplementary material for: Oxa-Michael-initiated cascade reactions of levoglucosenone
Source: Beilstein J Org Chem. 2022 Oct 13;18:1457–62. doi: 10.3762/bjoc.18.151 (PMC9577383; doi:10.3762/bjoc.18.151)
Supplement: File 2 — 1H and 13C FID data and other spectra for new compounds. [file Beilstein_J_Org_Chem-18-1457-s002.zip › supporting information2/spectra files for new compounds - minimal/data for 5a/NMR data for 5a/NOESY/pdata/1/JK-LGODi01-Hughcheck_6_1.pdf]

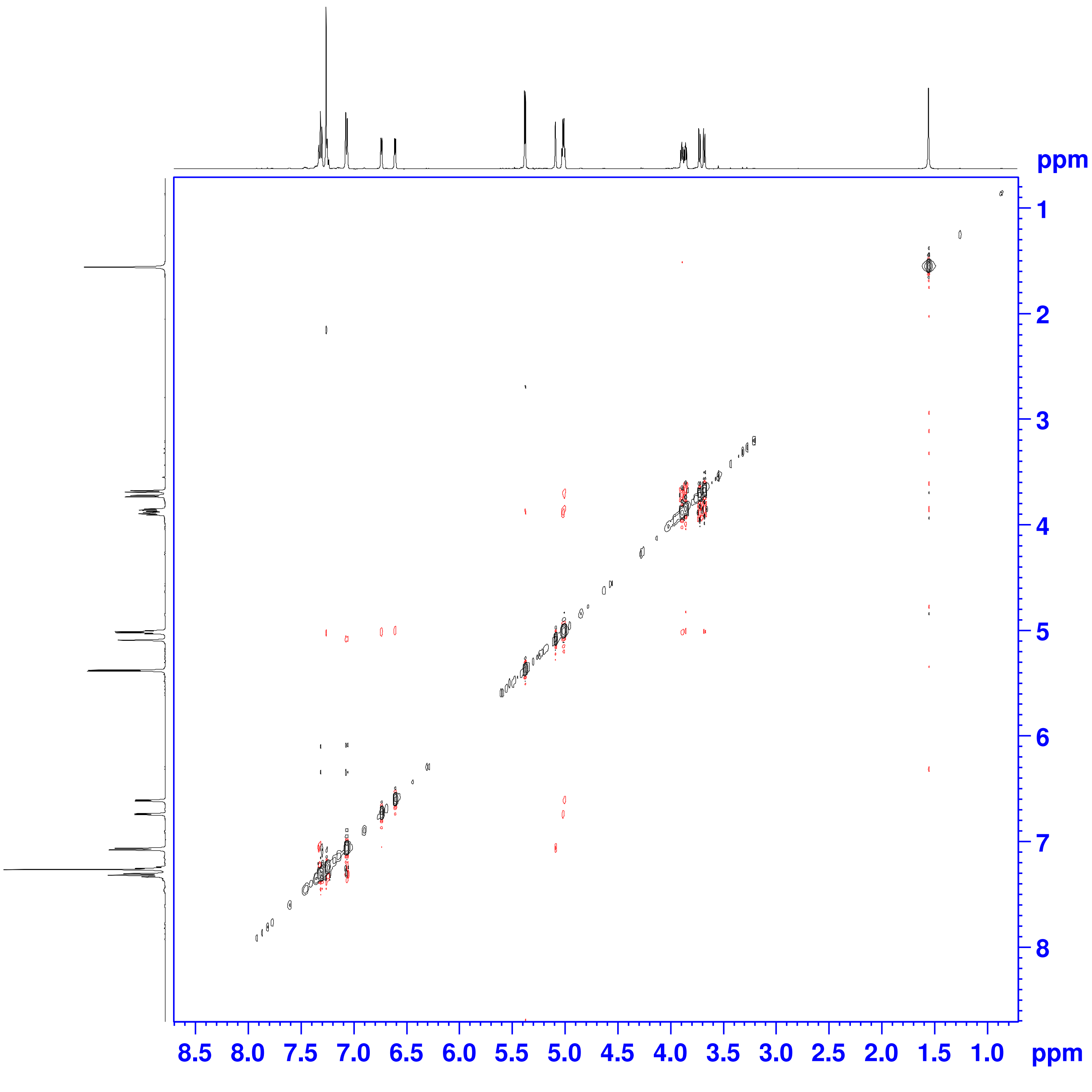


```
Current Data Parameters
NAME       JK-LGODi01-Hughcheck
EXPNO                     6
PROCNO                    1

F2 - Acquisition Parameters
Date_              20180626
Time                  22.10 h
INSTRUM               spect
PROBHD     Z119470_0196 (
PULPROG         noesygpphpp
TD                     2048
SOLVENT               CDCl3
NS                        4
DS                       32
SWH                4000.000 Hz
FIDRES             3.906250 Hz
AQ                0.2560000 sec
RG                    62.39
DW                  125.000 usec
DE                     6.50 usec
TE                    295.0 K
D0               0.00011201 sec
D1               1.94879997 sec
D8               0.30000001 sec
D11              0.03000000 sec
D12              0.00002000 sec
D16              0.00020000 sec
IN0              0.00025000 sec
TDav                      1
SFO1            500.1623659 MHz
NUC1                     1H
P1                    10.20 usec
P2                    20.40 usec
P17                 2500.00 usec
PLW1            22.00000000 W
PLW10            2.54320002 W
GPNAM[1]         SMSQ10.100
GPZ1                  40.00 %
P16                 1000.00 usec

F1 - Acquisition parameters
TD                      256
SFO1               500.1624 MHz
FIDRES            31.250000 Hz
SW                    7.997 ppm
FnMODE          States-TPPI

F2 - Processing parameters
SI                     1024
SF              500.1600113 MHz
WDW                   QSINE
SSB                       2
LB            0 Hz
GB            0
PC                     1.00

F1 - Processing parameters
SI                     1024
MC2             States-TPPI
SF              500.1600113 MHz
WDW                   QSINE
SSB                       2
LB            0 Hz
GB            0
```
