# Supplementary material for: Oxa-Michael-initiated cascade reactions of levoglucosenone
Source: Beilstein J Org Chem. 2022 Oct 13;18:1457–62. doi: 10.3762/bjoc.18.151 (PMC9577383; doi:10.3762/bjoc.18.151)
Supplement: File 2 — 1H and 13C FID data and other spectra for new compounds. [file Beilstein_J_Org_Chem-18-1457-s002.zip › supporting information2/spectra files for new compounds - minimal/data for 5b/HRMS-JK-LGODi04.pdf]

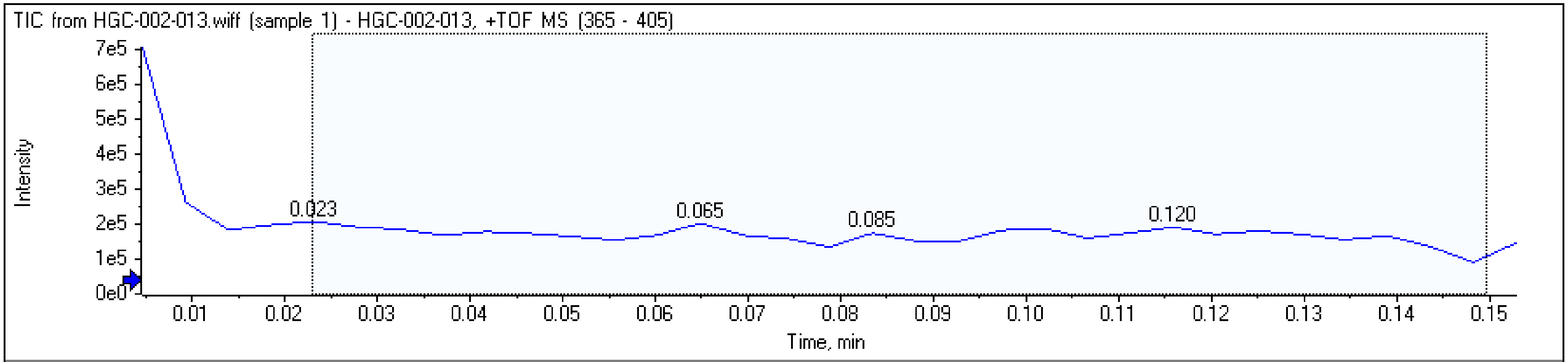

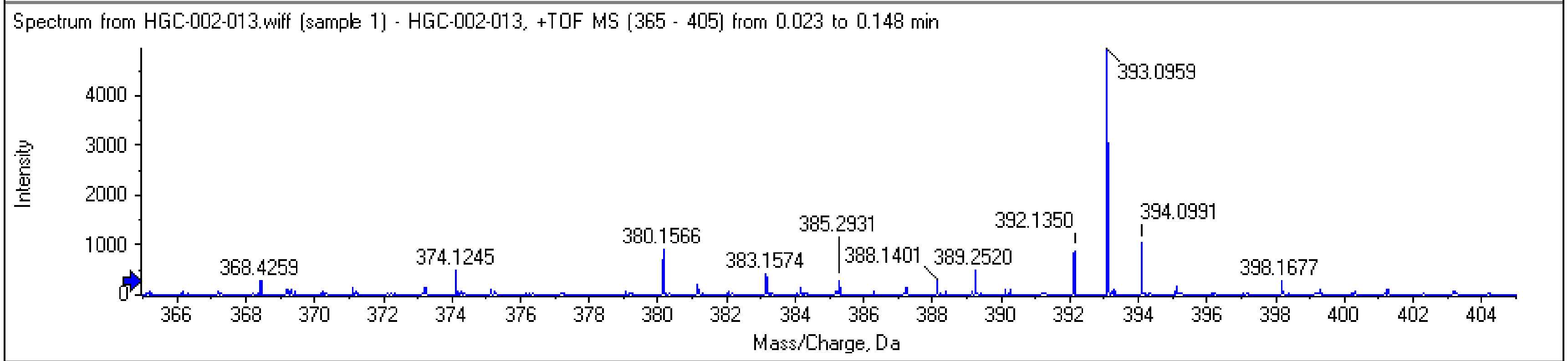

Mass Property | AA Property | Mass Accuracy | Isotopic Distribution | Elemental Composition | Hypermass | Unit Conversion | Custom Elements | AA Modifications

Measured m/z:	393.09590
Theoretical m/z:	393.0944
Error (mmu):	1.5
Error (ppm):	3.8

Calculate

5/29/2019 11:15:11 AM
