# Supplementary material for: Oxa-Michael-initiated cascade reactions of levoglucosenone
Source: Beilstein J Org Chem. 2022 Oct 13;18:1457–62. doi: 10.3762/bjoc.18.151 (PMC9577383; doi:10.3762/bjoc.18.151)
Supplement: File 2 — 1H and 13C FID data and other spectra for new compounds. [file Beilstein_J_Org_Chem-18-1457-s002.zip › supporting information2/spectra files for new compounds - minimal/data for 5c/ESI-JK-LGODio12.pdf]

MS Spectrum

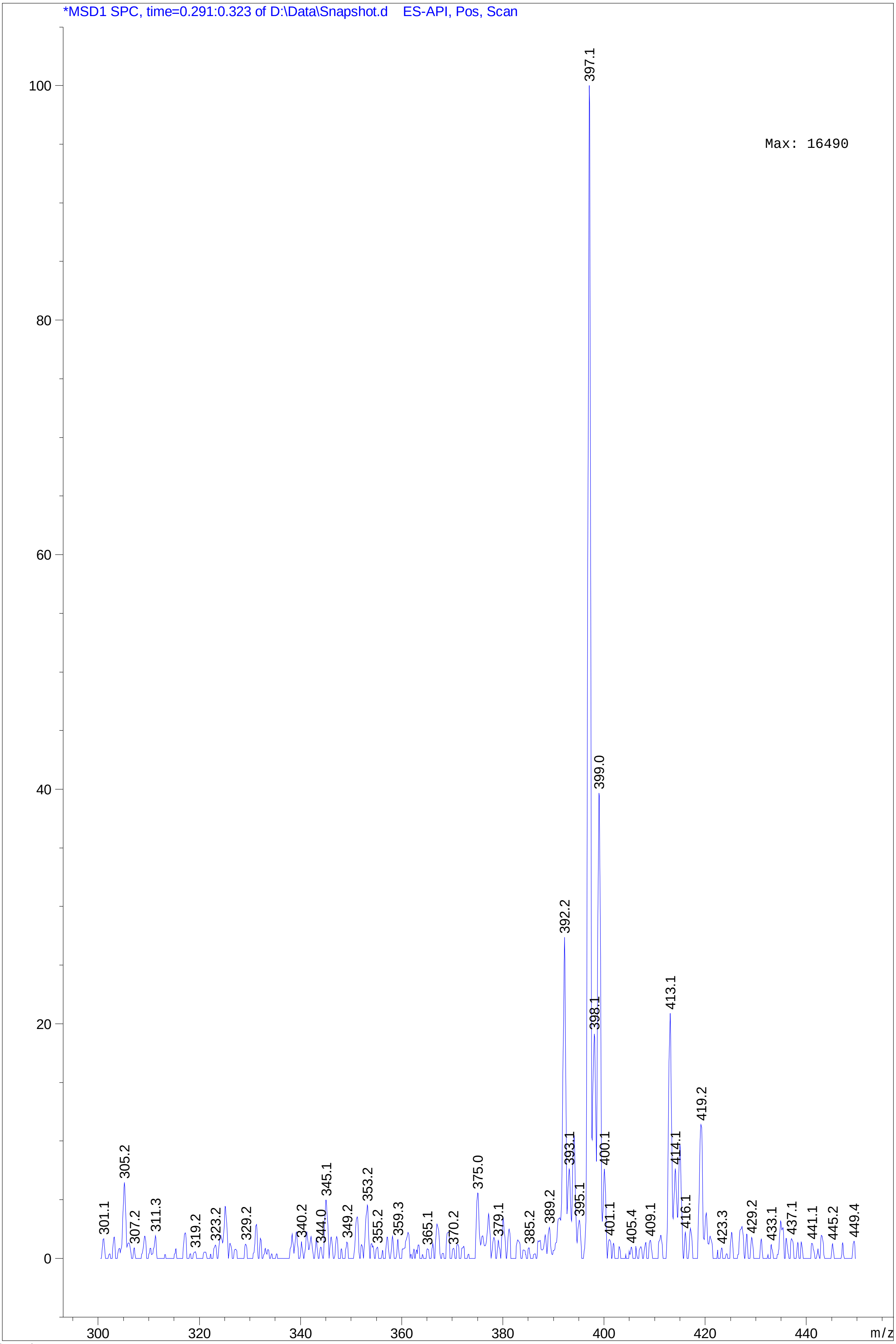
*MSD1 SPC, time=0.291:0.323 of D:\Data\Snapshot.d ES-API, Pos, Scan
Max: 16490
397.1
399.0
392.2
398.1
413.1
419.2
393.1
400.1
414.1
305.2
345.1
353.2
375.0
301.1
307.2
311.3
319.2
323.2
329.2
340.2
344.0
349.2
355.2
359.3
365.1
370.2
379.1
385.2
389.2
395.1
401.1
405.4
409.1
416.1
423.3
429.2
433.1
437.1
441.1
445.2
449.4
0
20
40
60
80
100
300
320
340
360
380
400
420
440
m/z
