# Supplementary material for: Oxa-Michael-initiated cascade reactions of levoglucosenone
Source: Beilstein J Org Chem. 2022 Oct 13;18:1457–62. doi: 10.3762/bjoc.18.151 (PMC9577383; doi:10.3762/bjoc.18.151)
Supplement: File 2 — 1H and 13C FID data and other spectra for new compounds. [file Beilstein_J_Org_Chem-18-1457-s002.zip › supporting information2/spectra files for new compounds - minimal/data for 5d/2/pdata/1/JK-LGODi19-S1_2_1.pdf]

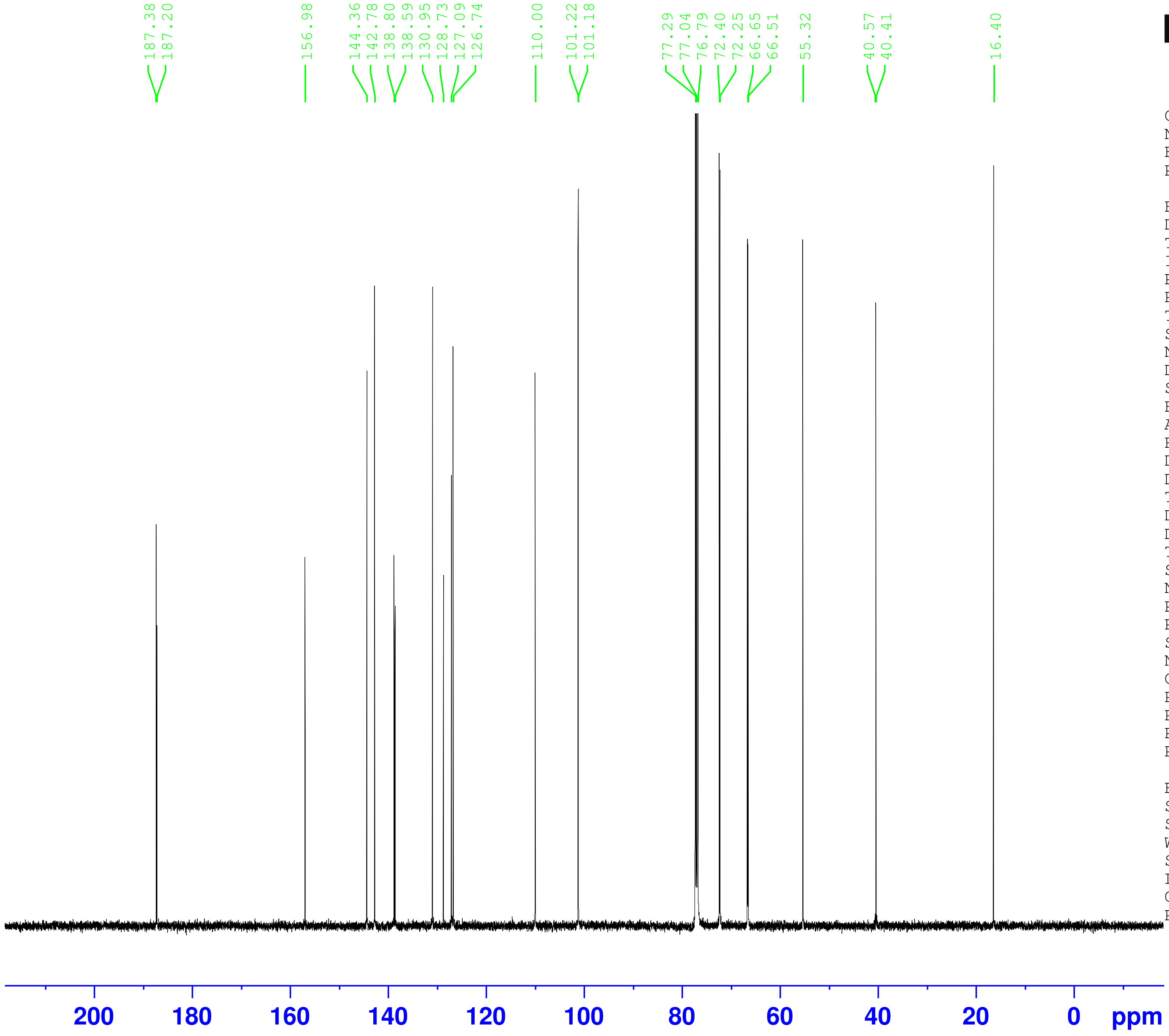

BRUKER

Current Data Parameters

NAME	JK-LGODi19-S1	
EXPNO	2	
PROCNO	1	

F2 - Acquisition Parameters

Date_	20180724	
Time	7.06	h
INSTRUM	spect	
PROBHD	Z119470_0196 (	
PULPROG	zgpg30	
TD	65536	
SOLVENT	CDCl3	
NS	6000	
DS	4	
SWH	29761.904	Hz
FIDRES	0.908261	Hz
AQ	1.1010048	sec
RG	191.36	
DW	16.800	usec
DE	6.50	usec
TE	293.1	K
D1	2.00000000	sec
D11	0.03000000	sec
TD0	1	
SFO1	125.7779086	MHz
NUC1	13C	
P1	10.00	usec
PLW1	78.00000000	W
SFO2	500.1620006	MHz
NUC2	1H	
CPDPRG[2	waltz16	
PCPD2	80.00	usec
PLW2	22.00000000	W
PLW12	0.35764000	W
PLW13	0.17989001	W

F2 - Processing parameters

SI	32768	
SF	125.7653320	MHz
WDW	EM	
SSB	0	
LB	1.00	Hz
GB	0	
PC	1.40	
