# Supplementary material for: Oxa-Michael-initiated cascade reactions of levoglucosenone
Source: Beilstein J Org Chem. 2022 Oct 13;18:1457–62. doi: 10.3762/bjoc.18.151 (PMC9577383; doi:10.3762/bjoc.18.151)
Supplement: File 2 — 1H and 13C FID data and other spectra for new compounds. [file Beilstein_J_Org_Chem-18-1457-s002.zip › supporting information2/spectra files for new compounds - minimal/data for 5e/ESI-JK-LGODio09.pdf]

MS Spectrum

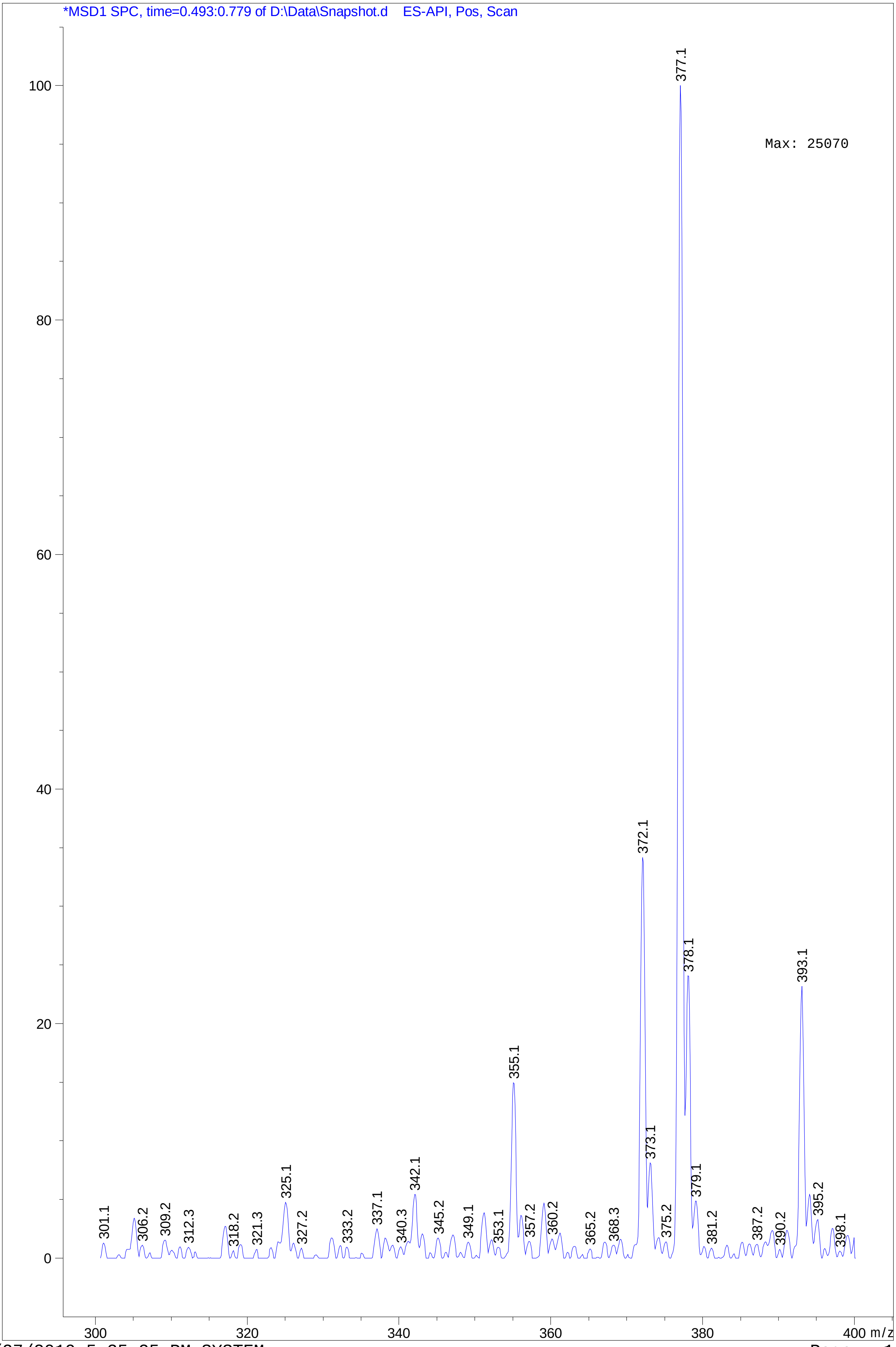
*MSD1 SPC, time=0.493:0.779 of D:\Data\Snapshot.d ES-API, Pos, Scan
Max: 25070
377.1
372.1
378.1
393.1
355.1
373.1
379.1
342.1
325.1
395.2
301.1
306.2
309.2
312.3
318.2
321.3
327.2
333.2
337.1
340.3
345.2
349.1
353.1
357.2
360.2
365.2
368.3
375.2
381.2
387.2
390.2
398.1
0
20
40
60
80
100
300
320
340
360
380
400 m/z
