# Supplementary material for: Oxa-Michael-initiated cascade reactions of levoglucosenone
Source: Beilstein J Org Chem. 2022 Oct 13;18:1457–62. doi: 10.3762/bjoc.18.151 (PMC9577383; doi:10.3762/bjoc.18.151)
Supplement: File 2 — 1H and 13C FID data and other spectra for new compounds. [file Beilstein_J_Org_Chem-18-1457-s002.zip › supporting information2/spectra files for new compounds - minimal/data for 5e/HRMS-JK-LGODi09.pdf]

TIC from JK-LGODi09.wiff (sample 1) - JK-LGODi09, +TOF MS (340 - 420)

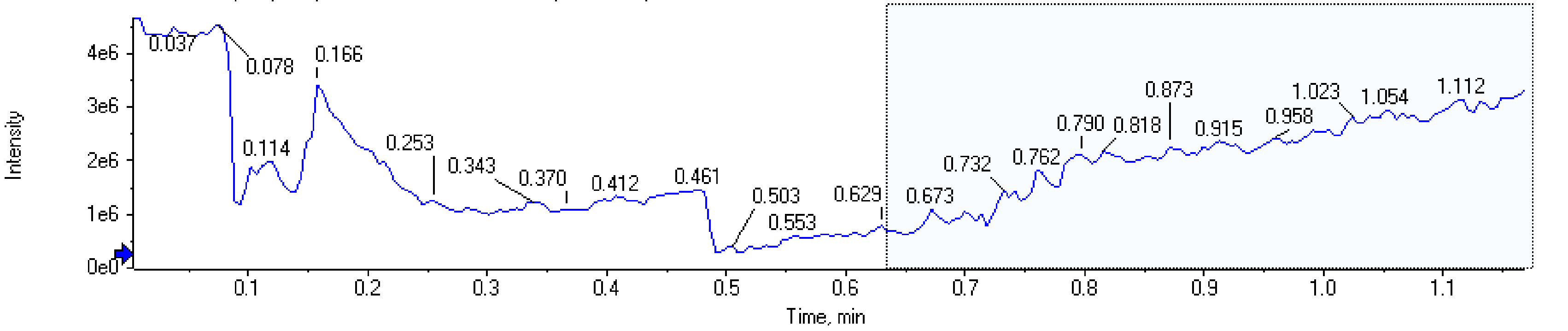

Spectrum from JK-LGODi09.wiff (sample 1) - JK-LGODi09, +TOF MS (340 - 420) from 0.635 to 1.168 min

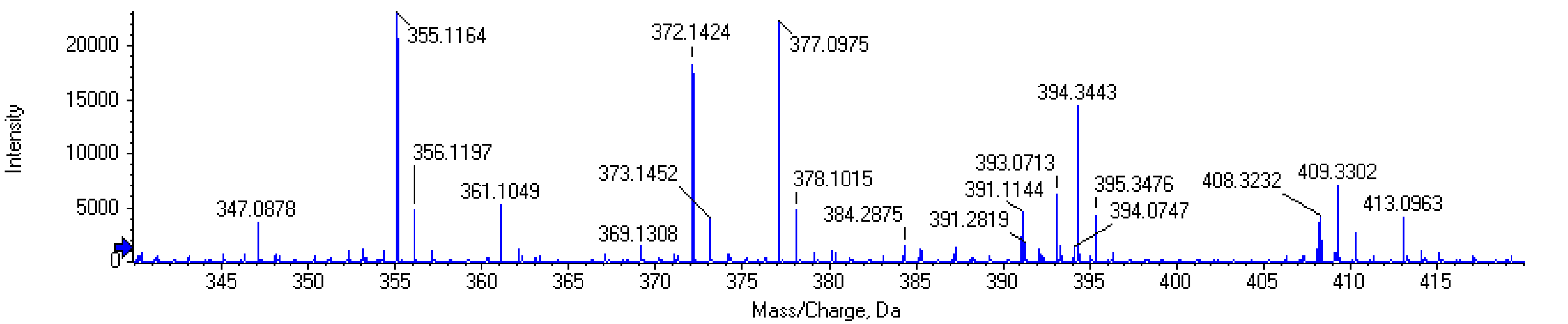

Mass Property | AA Property | Mass Accuracy | Isotopic Distribution | Elemental Composition | Hypermass | Unit Conversion | Custom Elements | AA Modifications

Measured m/z:	355.1164	Calculate
Theoretical m/z:	355.1176	
Error (mmu):	-1.2	
Error (ppm):	-3.4	

5/29/2019 11:26:32 AM
