# Supplementary material for: Oxa-Michael-initiated cascade reactions of levoglucosenone
Source: Beilstein J Org Chem. 2022 Oct 13;18:1457–62. doi: 10.3762/bjoc.18.151 (PMC9577383; doi:10.3762/bjoc.18.151)
Supplement: File 2 — 1H and 13C FID data and other spectra for new compounds. [file Beilstein_J_Org_Chem-18-1457-s002.zip › supporting information2/spectra files for new compounds - minimal/data for 5f/1/pdata/1/JK-LGODi07-S1_1_1.pdf]

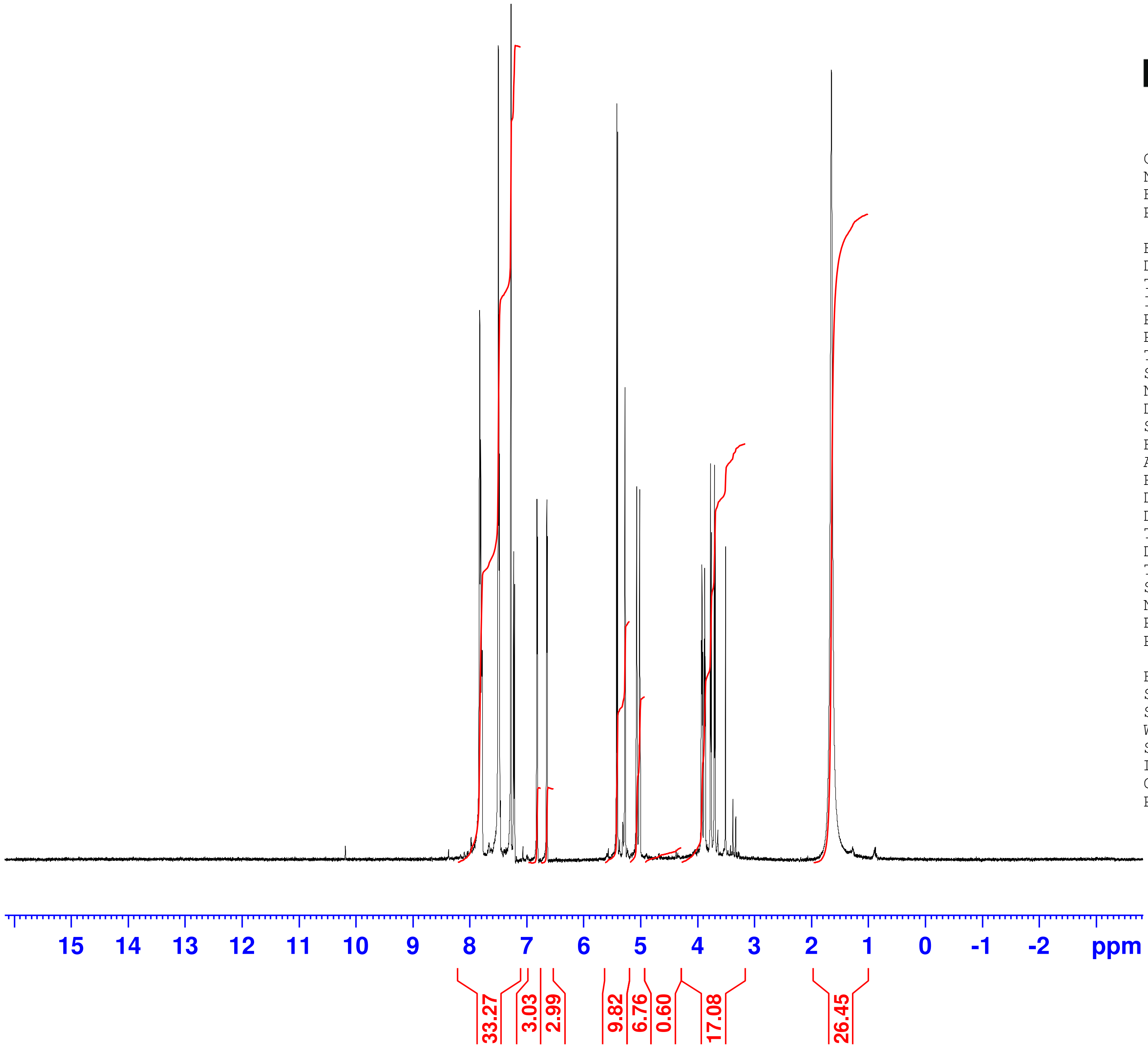

BRUKER

```
Current Data Parameters
NAME        JK-LGODi07-S1
EXPNO                   1
PROCNO                  1

F2 - Acquisition Parameters
Date_            20180714
Time                13.38 h
INSTRUM             spect
PROBHD   Z119470_0196 (
PULPROG              zg30
TD                  65536
SOLVENT             CDCl3
NS                     16
DS                      2
SWH             10000.000 Hz
FIDRES           0.305176 Hz
AQ              3.2767999 sec
RG                 138.85
DW                 50.000 usec
DE                   6.50 usec
TE                  295.0 K
D1             1.00000000 sec
TD0                     1
SFO1          500.1630885 MHz
NUC1                   1H
P1                  10.20 usec
PLW1          22.00000000 W

F2 - Processing parameters
SI                  65536
SF            500.1600000 MHz
WDW                    EM
SSB      0
LB                   0.30 Hz
GB       0
PC                   1.00
```
