# Supplementary material for: Oxa-Michael-initiated cascade reactions of levoglucosenone
Source: Beilstein J Org Chem. 2022 Oct 13;18:1457–62. doi: 10.3762/bjoc.18.151 (PMC9577383; doi:10.3762/bjoc.18.151)
Supplement: File 2 — 1H and 13C FID data and other spectra for new compounds. [file Beilstein_J_Org_Chem-18-1457-s002.zip › supporting information2/spectra files for new compounds - minimal/data for 5g/1/pdata/1/JK-LGODi15-S1_1_1.pdf]

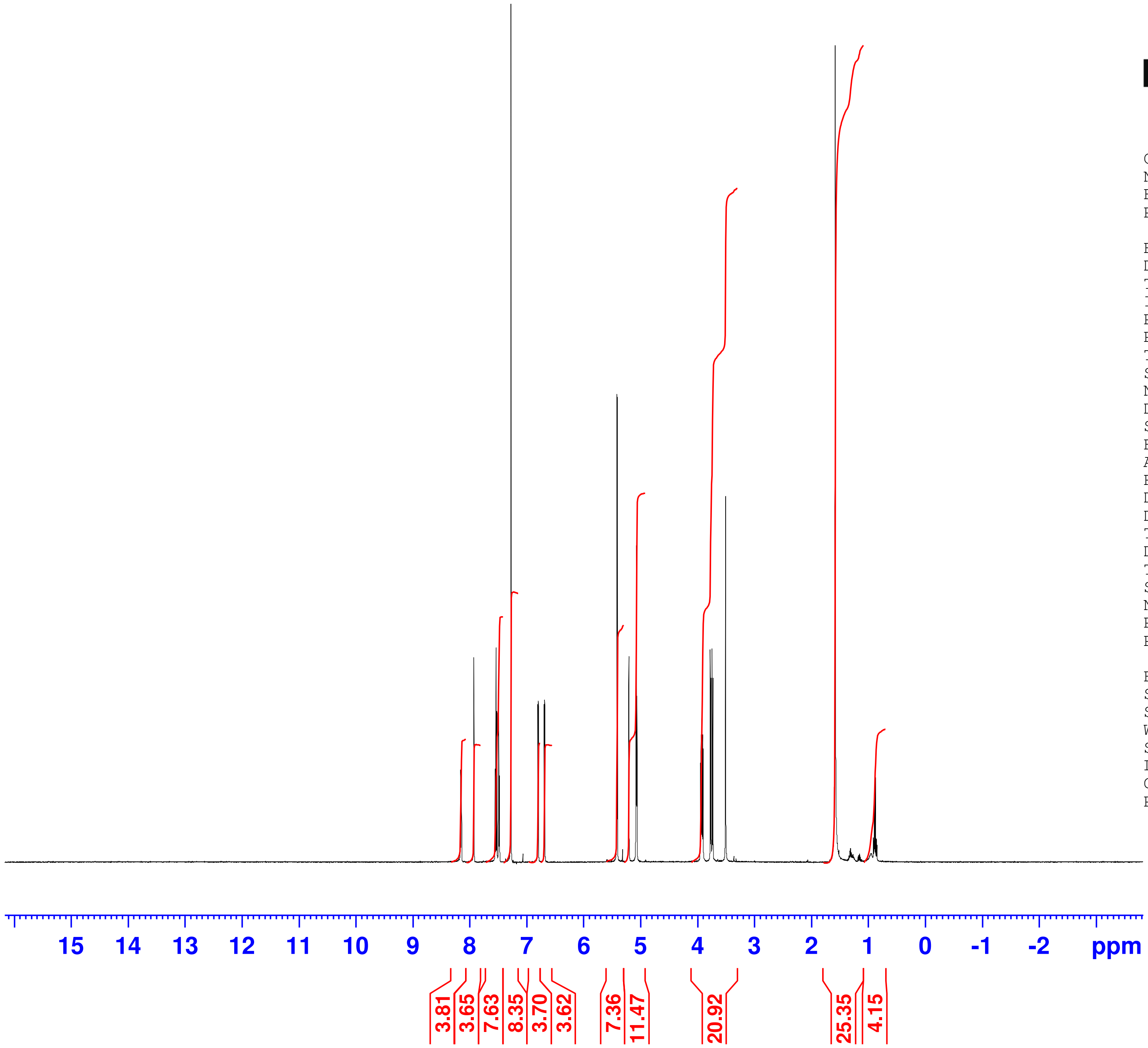

Current Data Parameters

NAME	JK-LGODi15-S1
EXPNO	1
PROCNO	1

F2 - Acquisition Parameters

Date_	20180718
Time	10.07 h
INSTRUM	spect
PROBHD	Z119470_0196 (
PULPROG	zg30
TD	65536
SOLVENT	CDCl3
NS	16
DS	2
SWH	10000.000 Hz
FIDRES	0.305176 Hz
AQ	3.2767999 sec
RG	123.82
DW	50.000 usec
DE	6.50 usec
TE	295.0 K
D1	1.00000000 sec
TD0	1
SFO1	500.1630885 MHz
NUC1	1H
P1	10.20 usec
PLW1	22.00000000 W

F2 - Processing parameters

SI	65536
SF	500.1600000 MHz
WDW	EM
SSB	0
LB	0.30 Hz
GB	0
PC	1.00
