# Supplementary material for: Oxa-Michael-initiated cascade reactions of levoglucosenone
Source: Beilstein J Org Chem. 2022 Oct 13;18:1457–62. doi: 10.3762/bjoc.18.151 (PMC9577383; doi:10.3762/bjoc.18.151)
Supplement: File 2 — 1H and 13C FID data and other spectra for new compounds. [file Beilstein_J_Org_Chem-18-1457-s002.zip › supporting information2/spectra files for new compounds - minimal/data for 5g/2/pdata/1/JK-LGODi15-S1_2_1.pdf]

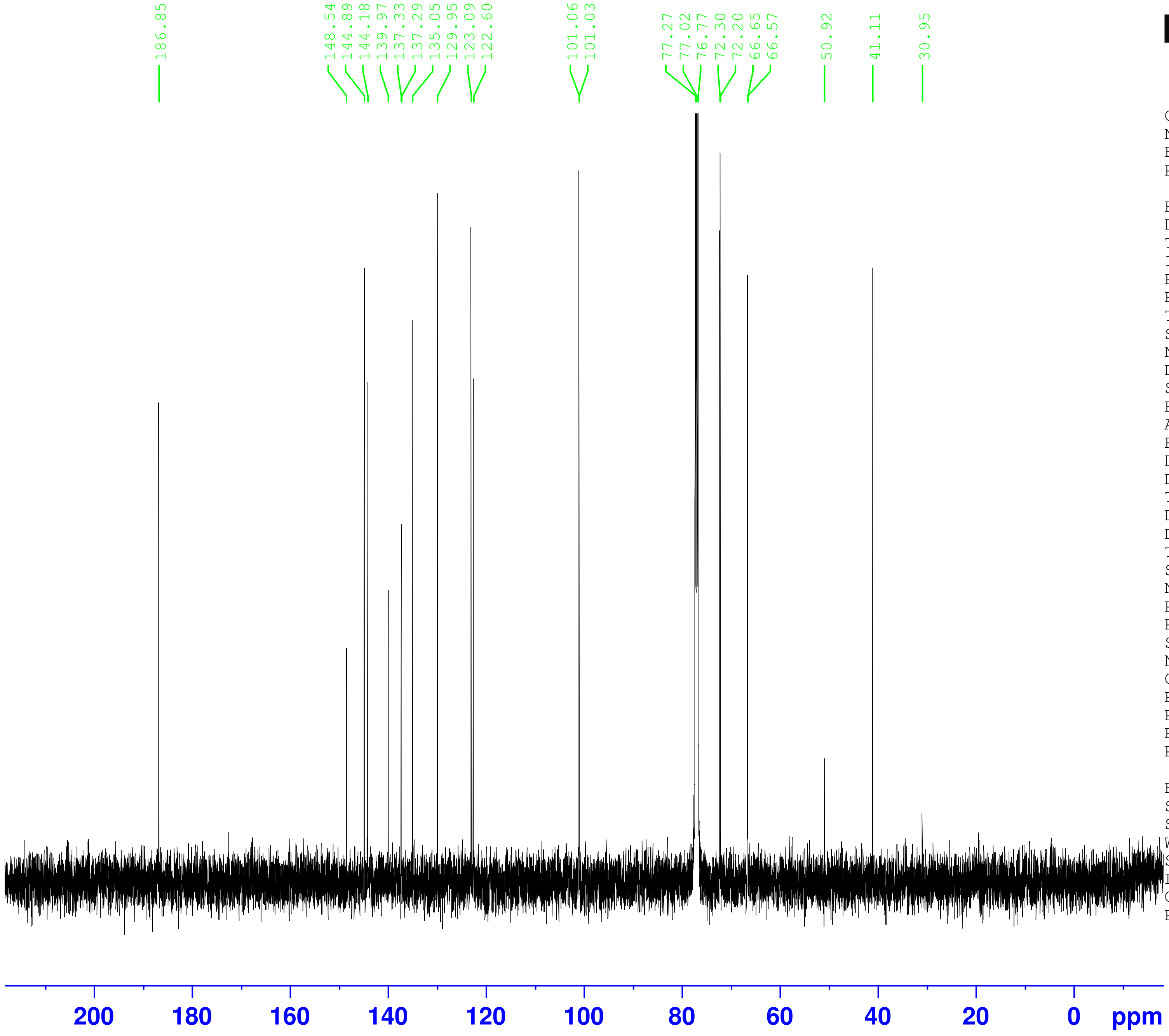


Current Data Parameters

| | |
|---|---|
| NAME | JK-LGODi15-S1 |
| EXPNO | 2 |
| PROCNO | 1 |

F2 - Acquisition Parameters

| | |
|---|---|
| Date_ | 20180719 |
| Time | 8.02 h |
| INSTRUM | spect |
| PROBHD | Z119470_0196 ( |
| PULPROG | zgpg30 |
| TD | 65536 |
| SOLVENT | CDCl3 |
| NS | 5000 |
| DS | 4 |
| SWH | 29761.904 Hz |
| FIDRES | 0.908261 Hz |
| AQ | 1.1010048 sec |
| RG | 191.36 |
| DW | 16.800 usec |
| DE | 6.50 usec |
| TE | 295.0 K |
| D1 | 2.00000000 sec |
| D11 | 0.03000000 sec |
| TD0 | 1 |
| SFO1 | 125.7779086 MHz |
| NUC1 | 13C |
| P1 | 10.00 usec |
| PLW1 | 78.00000000 W |
| SFO2 | 500.1620006 MHz |
| NUC2 | 1H |
| CPDPRG[2 | waltz16 |
| PCPD2 | 80.00 usec |
| PLW2 | 22.00000000 W |
| PLW12 | 0.35764000 W |
| PLW13 | 0.17989001 W |

F2 - Processing parameters

| | |
|---|---|
| SI | 32768 |
| SF | 125.7653320 MHz |
| WDW | EM |
| SSB | 0 |
| LB | 1.00 Hz |
| GB | 0 |
| PC | 1.40 |
