# Supplementary material for: Oxa-Michael-initiated cascade reactions of levoglucosenone
Source: Beilstein J Org Chem. 2022 Oct 13;18:1457–62. doi: 10.3762/bjoc.18.151 (PMC9577383; doi:10.3762/bjoc.18.151)
Supplement: File 2 — 1H and 13C FID data and other spectra for new compounds. [file Beilstein_J_Org_Chem-18-1457-s002.zip › supporting information2/spectra files for new compounds - minimal/data for 5g/ESI-5g.pdf]

MS Spectrum

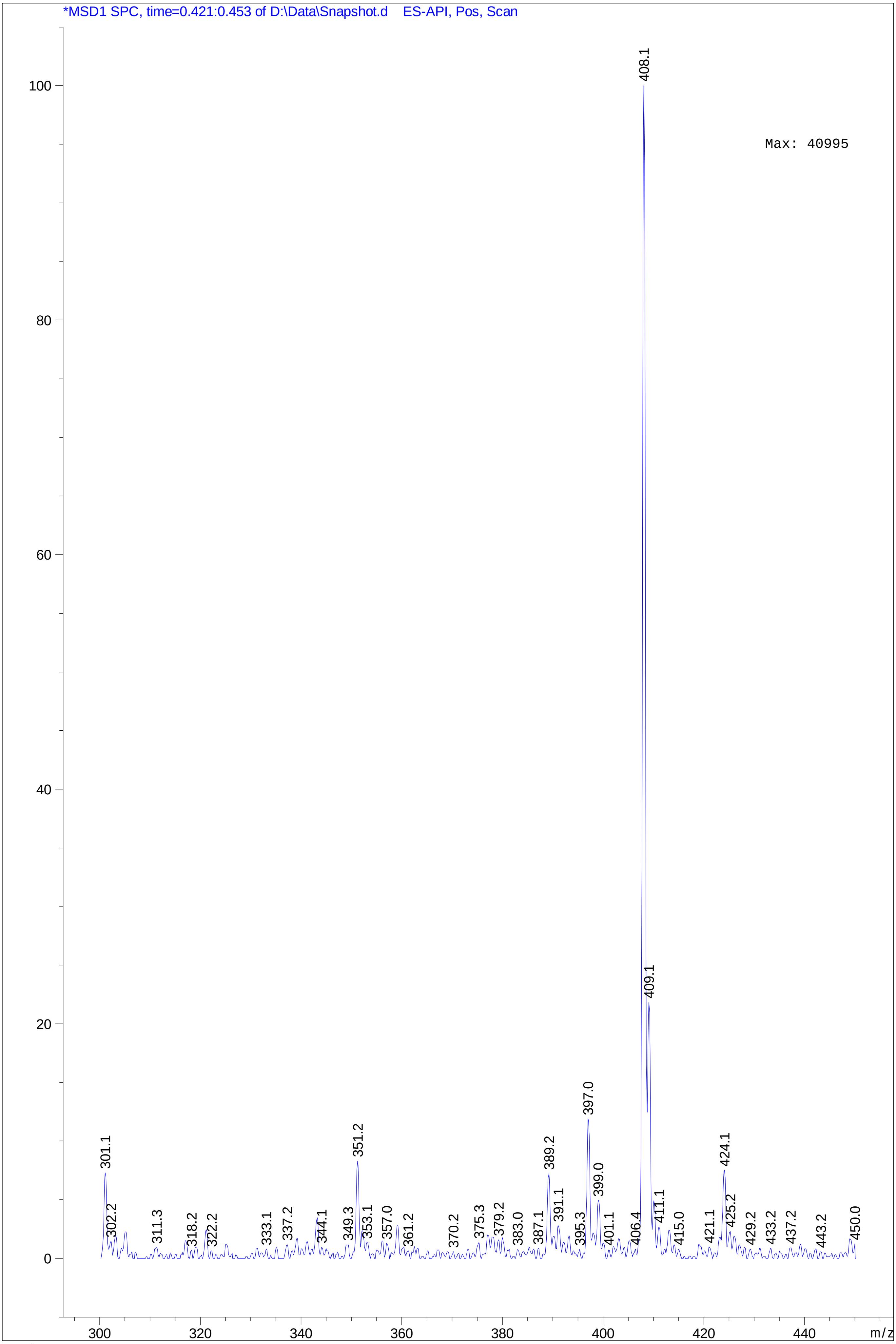
*MSD1 SPC, time=0.421:0.453 of D:\Data\Snapshot.d ES-API, Pos, Scan
Max: 40995
408.1
409.1
397.0
389.2
399.0
424.1
351.2
301.1
302.2
311.3
318.2
322.2
333.1
337.2
344.1
349.3
353.1
357.0
361.2
370.2
375.3
379.2
383.0
387.1
391.1
395.3
401.1
406.4
411.1
415.0
421.1
425.2
429.2
433.2
437.2
443.2
450.0
100
80
60
40
20
0
300
320
340
360
380
400
420
440
m/z
