# Supplementary material for: Oxa-Michael-initiated cascade reactions of levoglucosenone
Source: Beilstein J Org Chem. 2022 Oct 13;18:1457–62. doi: 10.3762/bjoc.18.151 (PMC9577383; doi:10.3762/bjoc.18.151)
Supplement: File 2 — 1H and 13C FID data and other spectra for new compounds. [file Beilstein_J_Org_Chem-18-1457-s002.zip › supporting information2/spectra files for new compounds - minimal/data for 5j/1/pdata/1/JK-LGODi02-CHCl3solid_1_1.pdf]

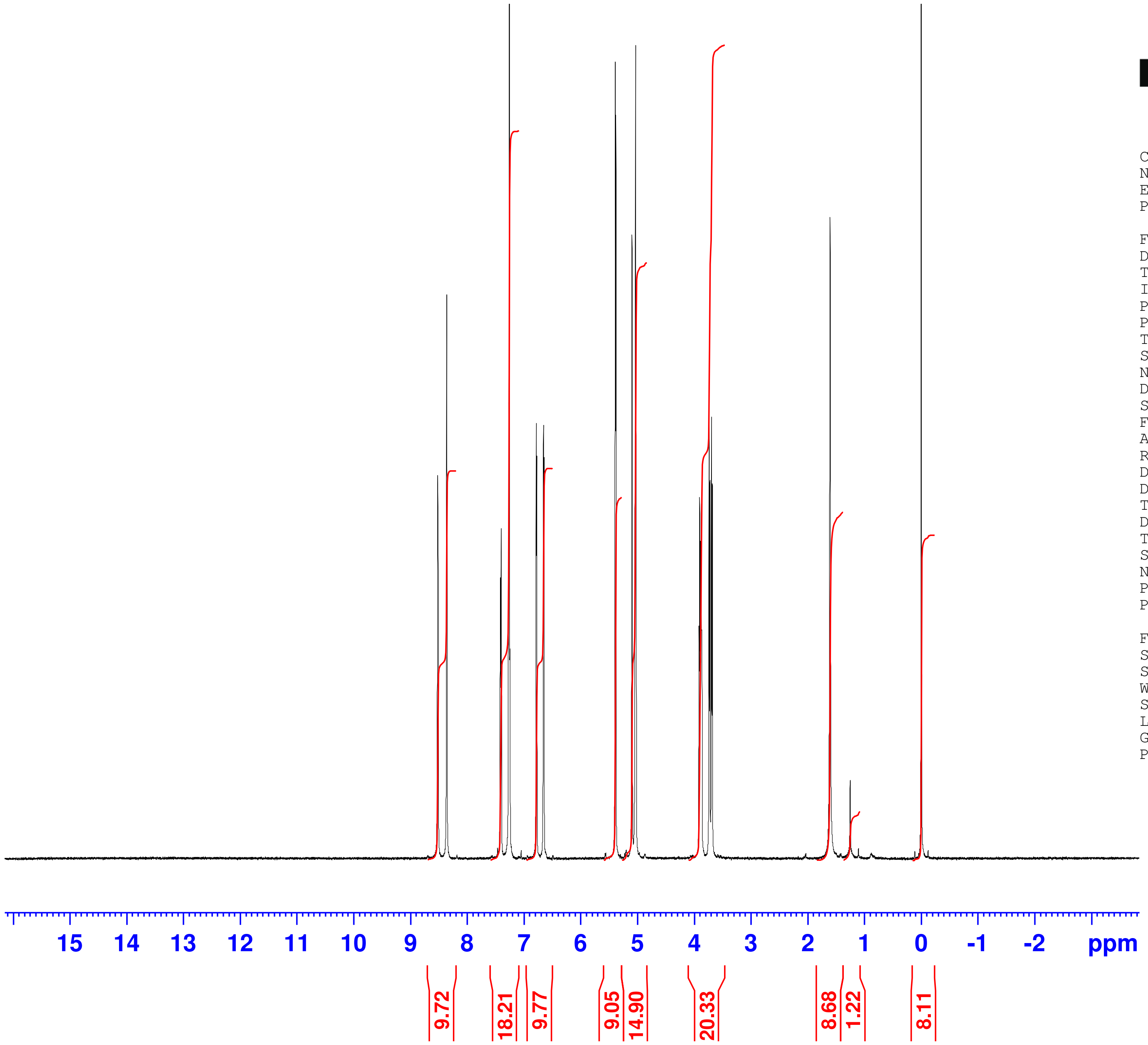


```
Current Data Parameters
NAME      JK-LGODi02-CHCl3solid
EXPNO                         1
PROCNO                        1

F2 - Acquisition Parameters
Date_              20180627
Time                  10.22 h
INSTRUM               spect
PROBHD    Z119470_0196 (
PULPROG                zg30
TD                    65536
SOLVENT               CDCl3
NS                       16
DS                        2
SWH            10000.000 Hz
FIDRES          0.305176 Hz
AQ             3.2767999 sec
RG                  138.85
DW                  50.000 usec
DE                    6.50 usec
TE                   295.0 K
D1            1.00000000 sec
TD0                       1
SFO1         500.1630885 MHz
NUC1                     1H
P1                   10.20 usec
PLW1         22.00000000 W

F2 - Processing parameters
SI                    65536
SF           500.1600092 MHz
WDW                      EM
SSB       0
LB                    0.30 Hz
GB        0
PC                     1.00
```
