# Supplementary material for: Oxa-Michael-initiated cascade reactions of levoglucosenone
Source: Beilstein J Org Chem. 2022 Oct 13;18:1457–62. doi: 10.3762/bjoc.18.151 (PMC9577383; doi:10.3762/bjoc.18.151)
Supplement: File 2 — 1H and 13C FID data and other spectra for new compounds. [file Beilstein_J_Org_Chem-18-1457-s002.zip › supporting information2/spectra files for new compounds - minimal/data for 5j/ESI-5j.pdf]

MS Spectrum

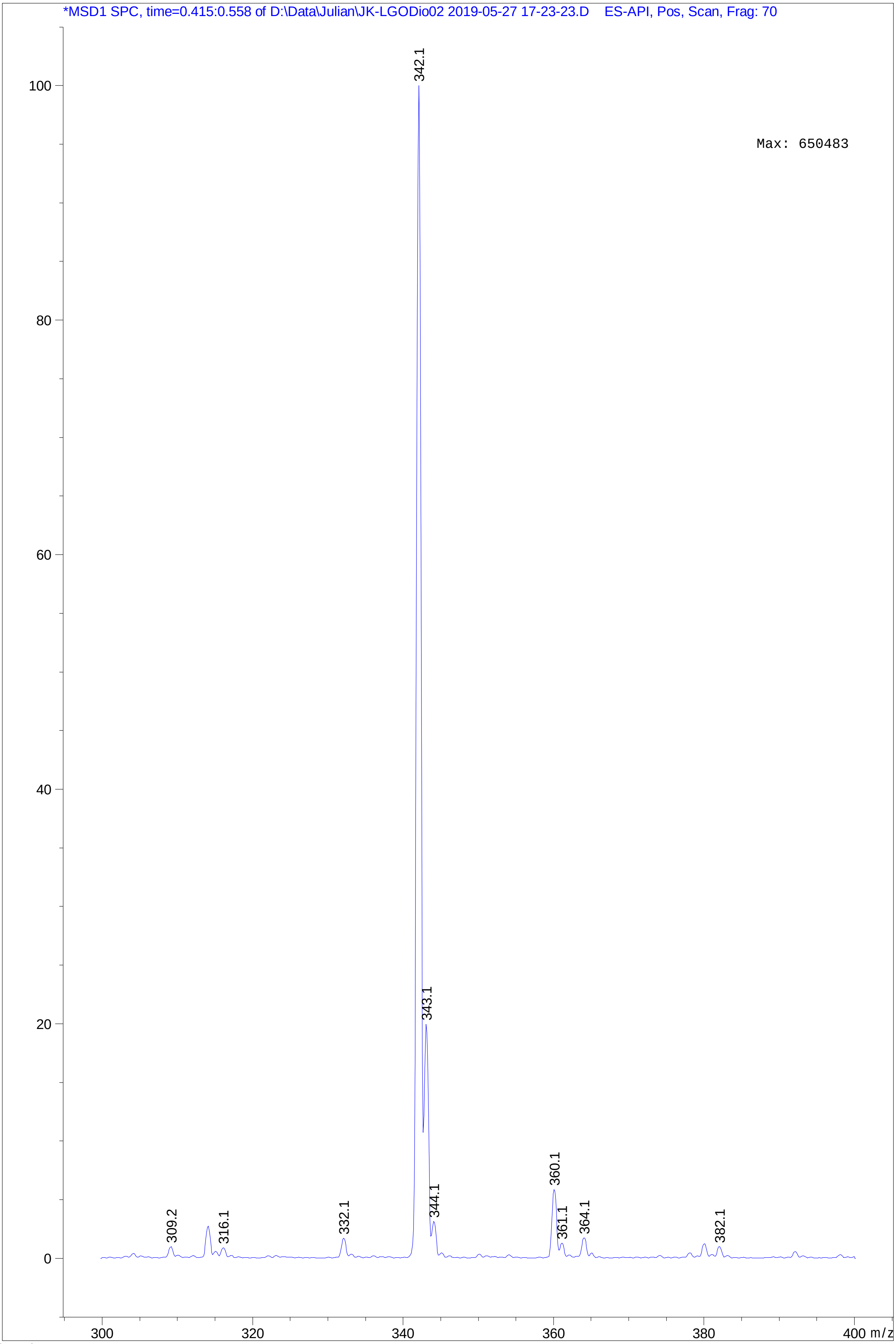
*MSD1 SPC, time=0.415:0.558 of D:\Data\Julian\JK-LGODio02 2019-05-27 17-23-23.D ES-API, Pos, Scan, Frag: 70
Max: 650483
342.1
343.1
344.1
360.1
361.1
364.1
309.2
316.1
332.1
382.1
100
80
60
40
20
0
300
320
340
360
380
400
m/z
