# Supplementary material for: Oxa-Michael-initiated cascade reactions of levoglucosenone
Source: Beilstein J Org Chem. 2022 Oct 13;18:1457–62. doi: 10.3762/bjoc.18.151 (PMC9577383; doi:10.3762/bjoc.18.151)
Supplement: File 2 — 1H and 13C FID data and other spectra for new compounds. [file Beilstein_J_Org_Chem-18-1457-s002.zip › supporting information2/spectra files for new compounds - minimal/data for 5j/IR-5j.pdf]

PerkinElmer Spectrum Version 10.4.00
Thursday, 19 July 2018 11:16 AM

**Analyst**	Student
**Date**	Thursday, 19 July 2018 11:16 AM

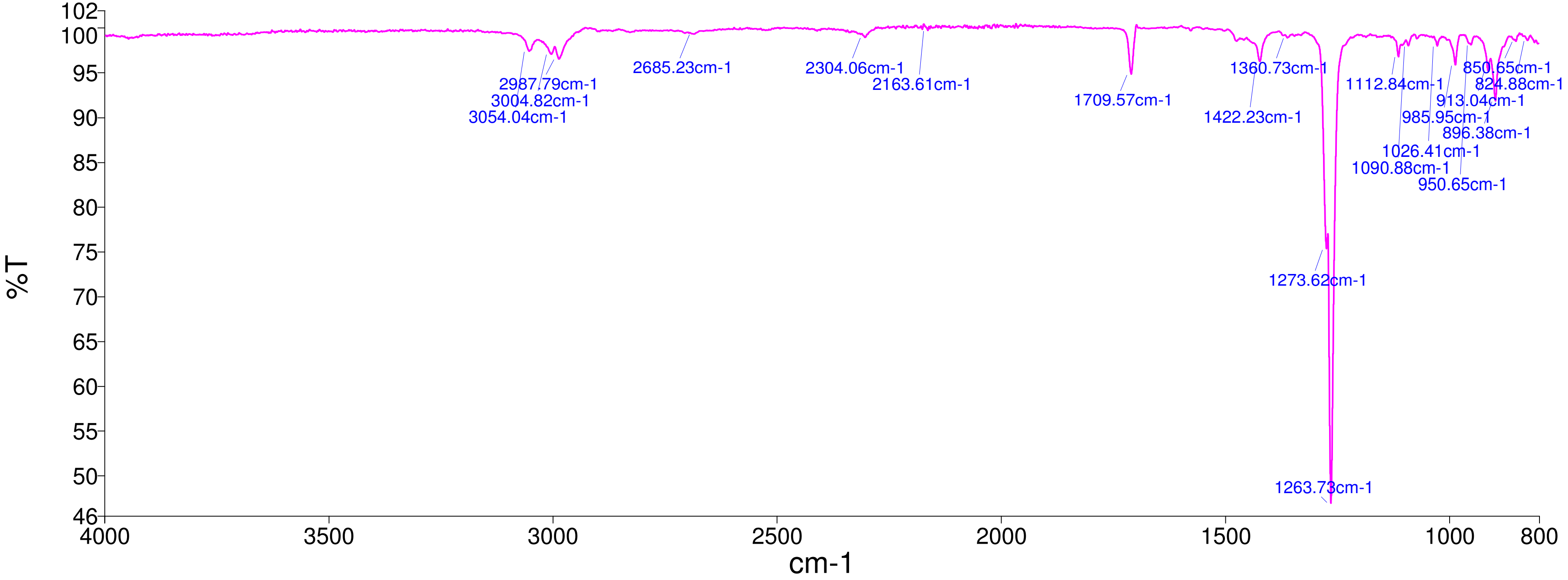

Sample Name	Description	Quality Checks
JK-LGODi02-2	011	The Quality Checks do not report any warnings for the sample.
