# Supplementary material for: Oxa-Michael-initiated cascade reactions of levoglucosenone
Source: Beilstein J Org Chem. 2022 Oct 13;18:1457–62. doi: 10.3762/bjoc.18.151 (PMC9577383; doi:10.3762/bjoc.18.151)
Supplement: File 2 — 1H and 13C FID data and other spectra for new compounds. [file Beilstein_J_Org_Chem-18-1457-s002.zip › supporting information2/spectra files for new compounds - minimal/data for 5k/1/pdata/1/email_JK-LGODi06-Preci1_1_1.pdf]

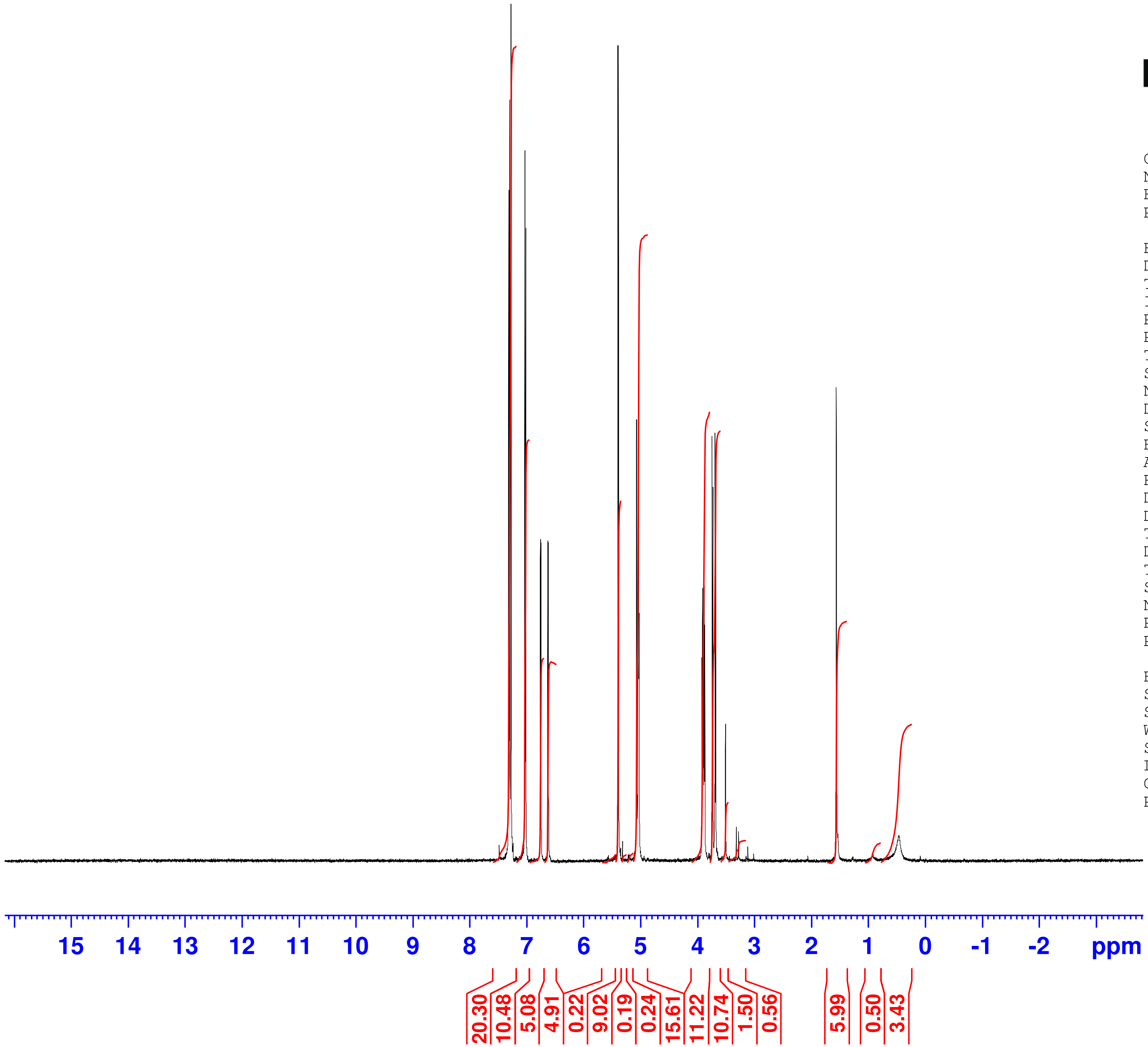

BRUKER

Current Data Parameters
NAME JK-LGODi06-Preci1
EXPNO 1
PROCNO 1

F2 - Acquisition Parameters
Date_ 20180713
Time 17.33 h
INSTRUM spect
PROBHD Z119470_0196 (
PULPROG zg30
TD 65536
SOLVENT CDCl3
NS 16
DS 2
SWH 10000.000 Hz
FIDRES 0.305176 Hz
AQ 3.2767999 sec
RG 123.82
DW 50.000 usec
DE 6.50 usec
TE 295.0 K
D1 1.00000000 sec
TD0 1
SFO1 500.1630885 MHz
NUC1 1H
P1 10.20 usec
PLW1 22.00000000 W

F2 - Processing parameters
SI 65536
SF 500.1600000 MHz
WDW EM
SSB 0
LB 0.30 Hz
GB 0
PC 1.00
