# Supplementary material for: Oxa-Michael-initiated cascade reactions of levoglucosenone
Source: Beilstein J Org Chem. 2022 Oct 13;18:1457–62. doi: 10.3762/bjoc.18.151 (PMC9577383; doi:10.3762/bjoc.18.151)
Supplement: File 2 — 1H and 13C FID data and other spectra for new compounds. [file Beilstein_J_Org_Chem-18-1457-s002.zip › supporting information2/spectra files for new compounds - minimal/data for 5k/ESI-5k.pdf]

MS Spectrum

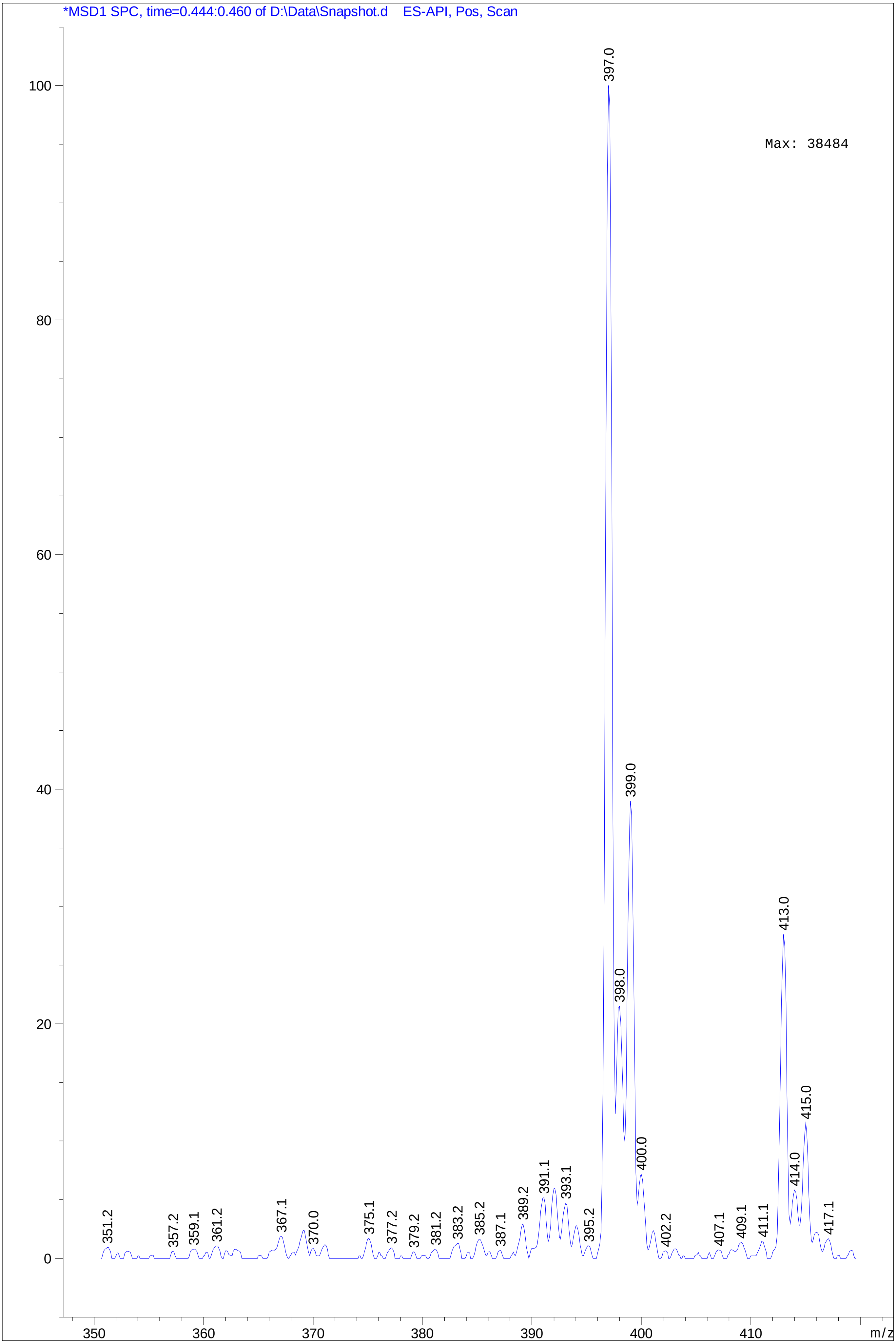
*MSD1 SPC, time=0.444:0.460 of D:\Data\Snapshot.d ES-API, Pos, Scan
Max: 38484
397.0
399.0
413.0
398.0
415.0
400.0
414.0
391.1
393.1
389.2
395.2
351.2
357.2
359.1
361.2
367.1
370.0
375.1
377.2
379.2
381.2
383.2
385.2
387.1
402.2
407.1
409.1
411.1
417.1
100
80
60
40
20
0
350
360
370
380
390
400
410
m/z
