# Supplementary material for: Oxa-Michael-initiated cascade reactions of levoglucosenone
Source: Beilstein J Org Chem. 2022 Oct 13;18:1457–62. doi: 10.3762/bjoc.18.151 (PMC9577383; doi:10.3762/bjoc.18.151)
Supplement: File 2 — 1H and 13C FID data and other spectra for new compounds. [file Beilstein_J_Org_Chem-18-1457-s002.zip › supporting information2/spectra files for new compounds - minimal/data for 5l/1/pdata/1/JK-LGODi22-S1_1_1.pdf]

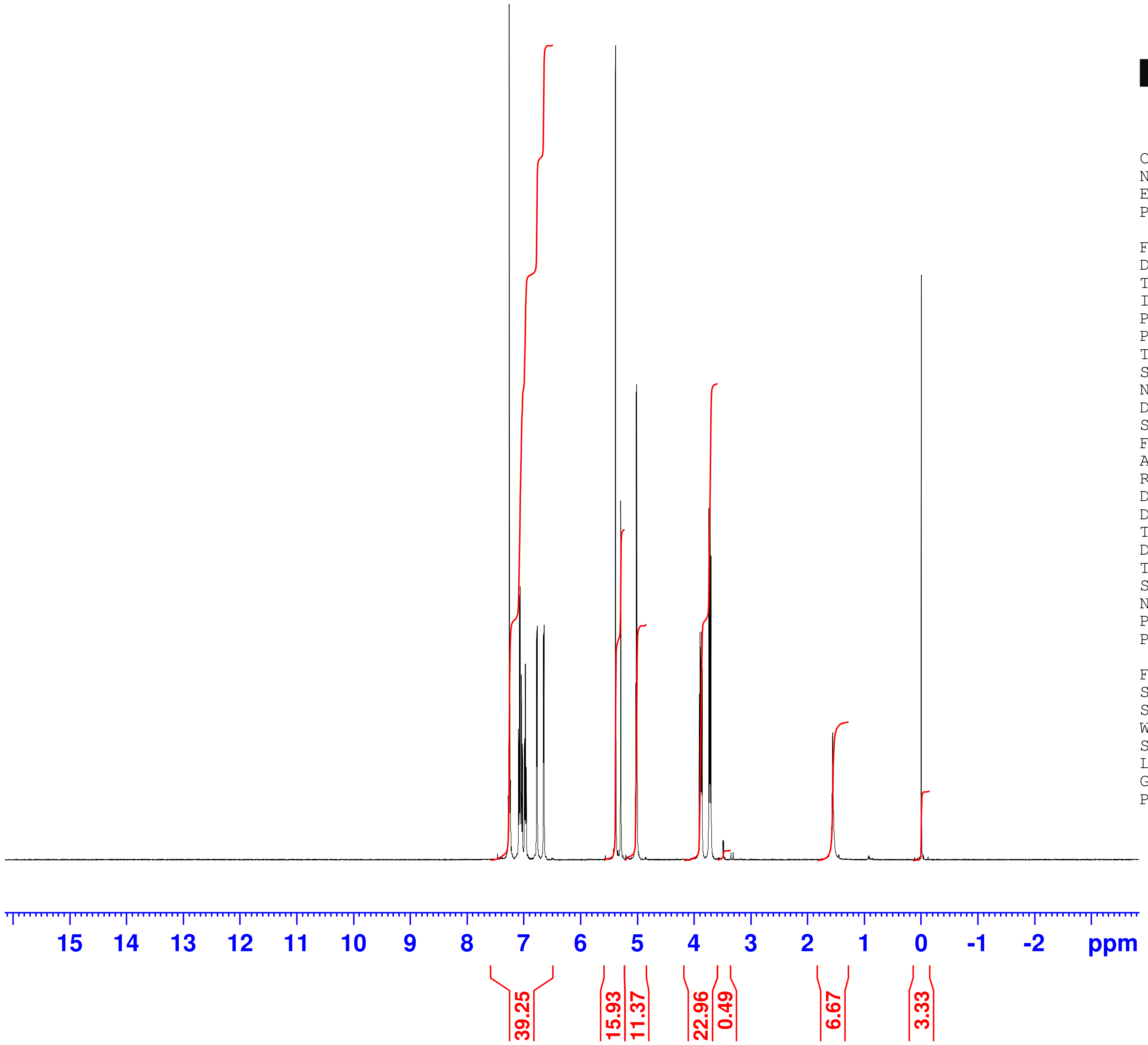

Current Data Parameters
NAME JK-LGODi22-S1
EXPNO 1
PROCNO 1

F2 - Acquisition Parameters

Date_	20180724	
Time	15.57	h
INSTRUM	spect	
PROBHD	Z119470_0196 (	
PULPROG	zg30	
TD	65536	
SOLVENT	CDCl3	
NS	16	
DS	2	
SWH	10000.000	Hz
FIDRES	0.305176	Hz
AQ	3.2767999	sec
RG	123.82	
DW	50.000	usec
DE	6.50	usec
TE	294.8	K
D1	1.00000000	sec
TD0	1	
SFO1	500.1630885	MHz
NUC1	1H	
P1	10.20	usec
PLW1	22.00000000	W

F2 - Processing parameters

SI	65536	
SF	500.1600109	MHz
WDW	EM	
SSB	0	
LB	0.30	Hz
GB	0	
PC	1.00	
