# Supplementary material for: Oxa-Michael-initiated cascade reactions of levoglucosenone
Source: Beilstein J Org Chem. 2022 Oct 13;18:1457–62. doi: 10.3762/bjoc.18.151 (PMC9577383; doi:10.3762/bjoc.18.151)
Supplement: File 2 — 1H and 13C FID data and other spectra for new compounds. [file Beilstein_J_Org_Chem-18-1457-s002.zip › supporting information2/spectra files for new compounds - minimal/data for 5l/2/pdata/1/JK-LGODi22-S1_2_1.pdf]

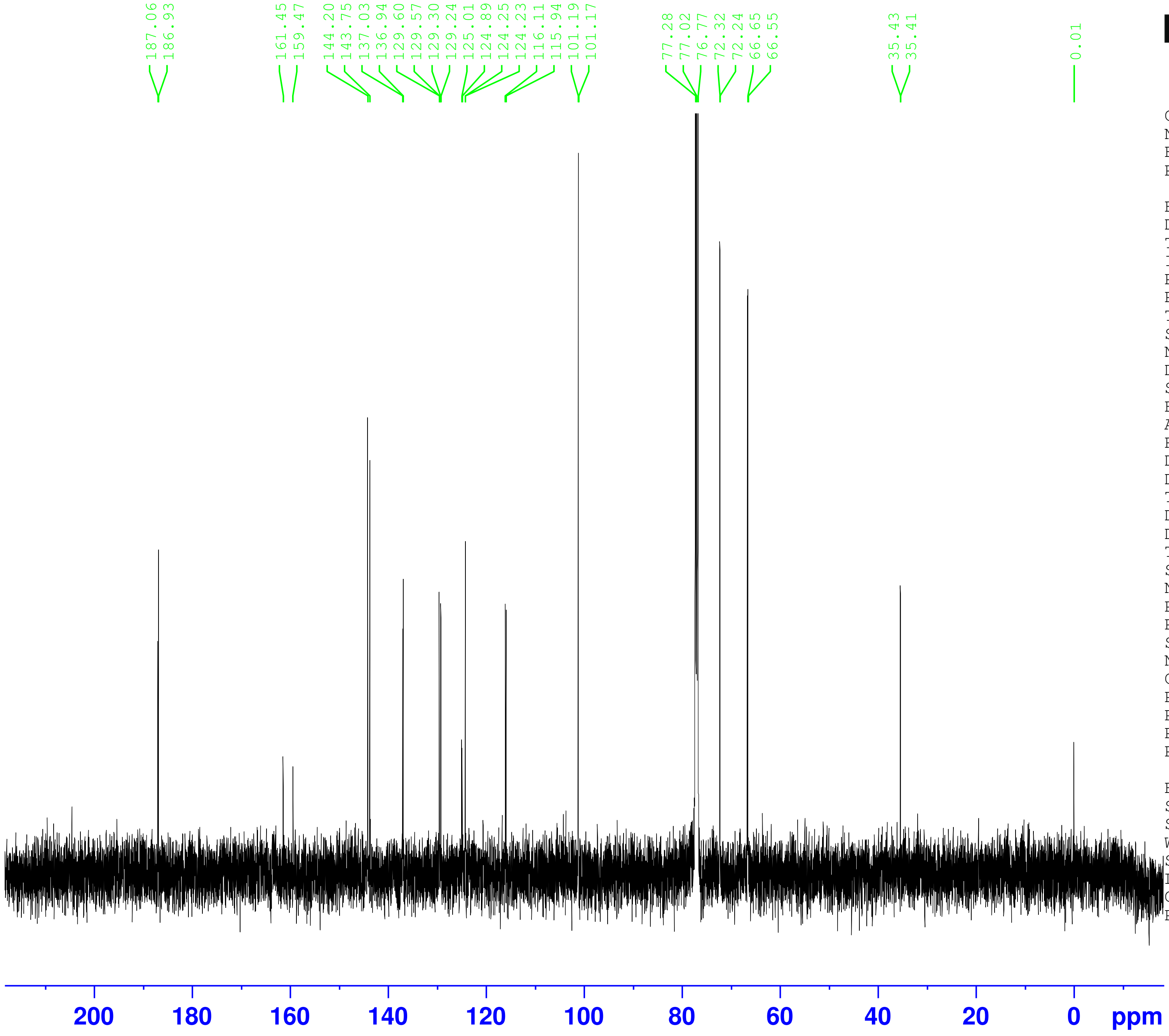

Current Data Parameters
NAME JK-LGODi22-S1
EXPNO 2
PROCNO 1

F2 - Acquisition Parameters
Date_ 20180725
Time 9.20 h
INSTRUM spect
PROBHD Z119470_0196 (
PULPROG zgpg30
TD 65536
SOLVENT CDCl3
NS 2000
DS 4
SWH 29761.904 Hz
FIDRES 0.908261 Hz
AQ 1.1010048 sec
RG 191.36
DW 16.800 usec
DE 6.50 usec
TE 293.7 K
D1 2.00000000 sec
D11 0.03000000 sec
TD0 1
SFO1 125.7779086 MHz
NUC1 13C
P1 10.00 usec
PLW1 78.00000000 W
SFO2 500.1620006 MHz
NUC2 1H
CPDPRG[2 waltz16
PCPD2 80.00 usec
PLW2 22.00000000 W
PLW12 0.35764000 W
PLW13 0.17989001 W

F2 - Processing parameters
SI 32768
SF 125.7653320 MHz
WDW EM
SSB 0
LB 1.00 Hz
GB 0
PC 1.40
