# Supplementary material for: Oxa-Michael-initiated cascade reactions of levoglucosenone
Source: Beilstein J Org Chem. 2022 Oct 13;18:1457–62. doi: 10.3762/bjoc.18.151 (PMC9577383; doi:10.3762/bjoc.18.151)
Supplement: File 2 — 1H and 13C FID data and other spectra for new compounds. [file Beilstein_J_Org_Chem-18-1457-s002.zip › supporting information2/spectra files for new compounds - minimal/data for 5l/ESI-5l.pdf]

MS Spectrum

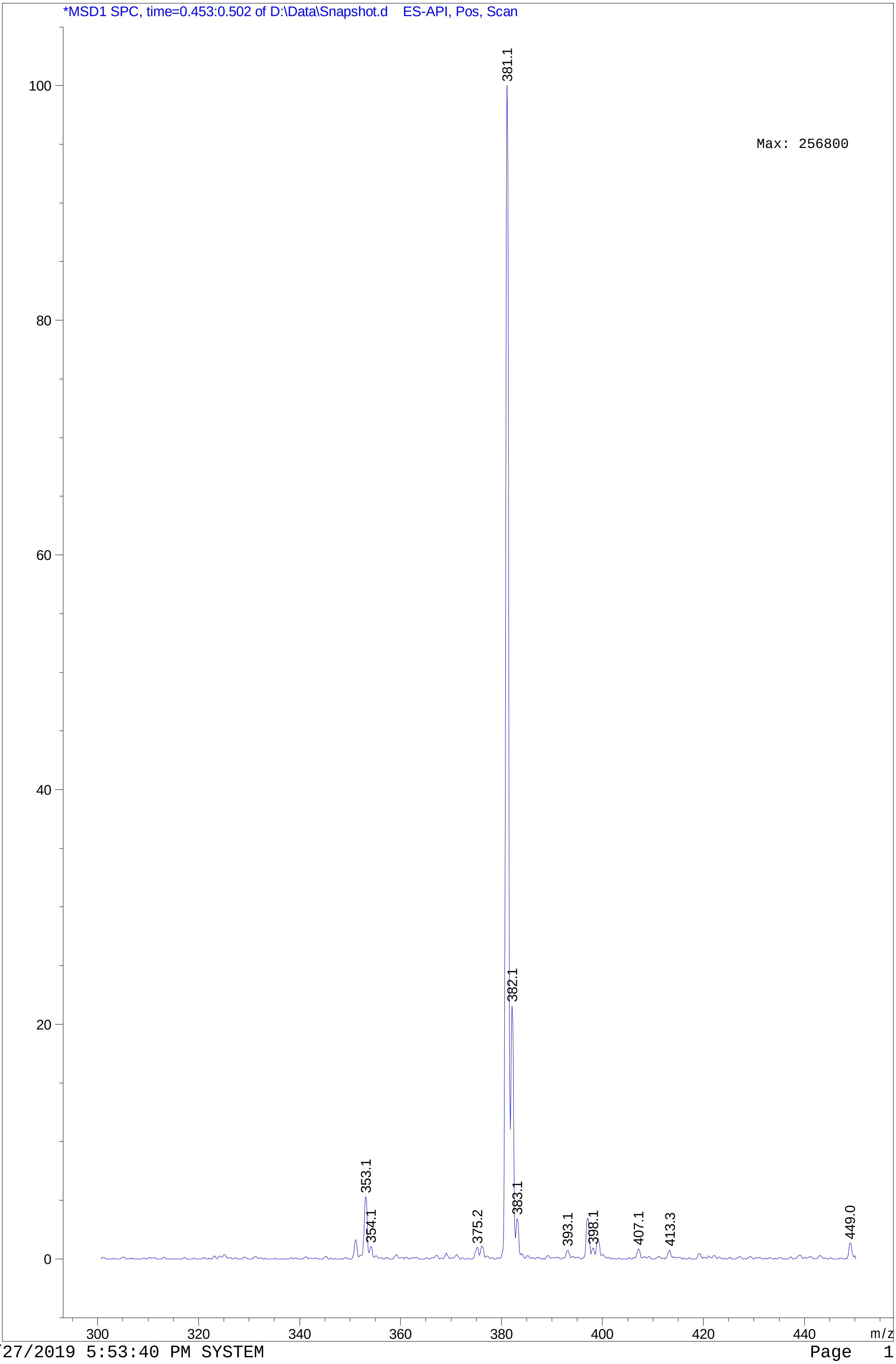
*MSD1 SPC, time=0.453:0.502 of D:\Data\Snapshot.d ES-API, Pos, Scan
Max: 256800
381.1
382.1
353.1
354.1
375.2
383.1
393.1
398.1
407.1
413.3
449.0
100
80
60
40
20
0
300
320
340
360
380
400
420
440
m/z
