# Supplementary material for: Oxa-Michael-initiated cascade reactions of levoglucosenone
Source: Beilstein J Org Chem. 2022 Oct 13;18:1457–62. doi: 10.3762/bjoc.18.151 (PMC9577383; doi:10.3762/bjoc.18.151)
Supplement: File 2 — 1H and 13C FID data and other spectra for new compounds. [file Beilstein_J_Org_Chem-18-1457-s002.zip › supporting information2/spectra files for new compounds - minimal/data for 5m/ESI-5m.pdf]

MS Spectrum

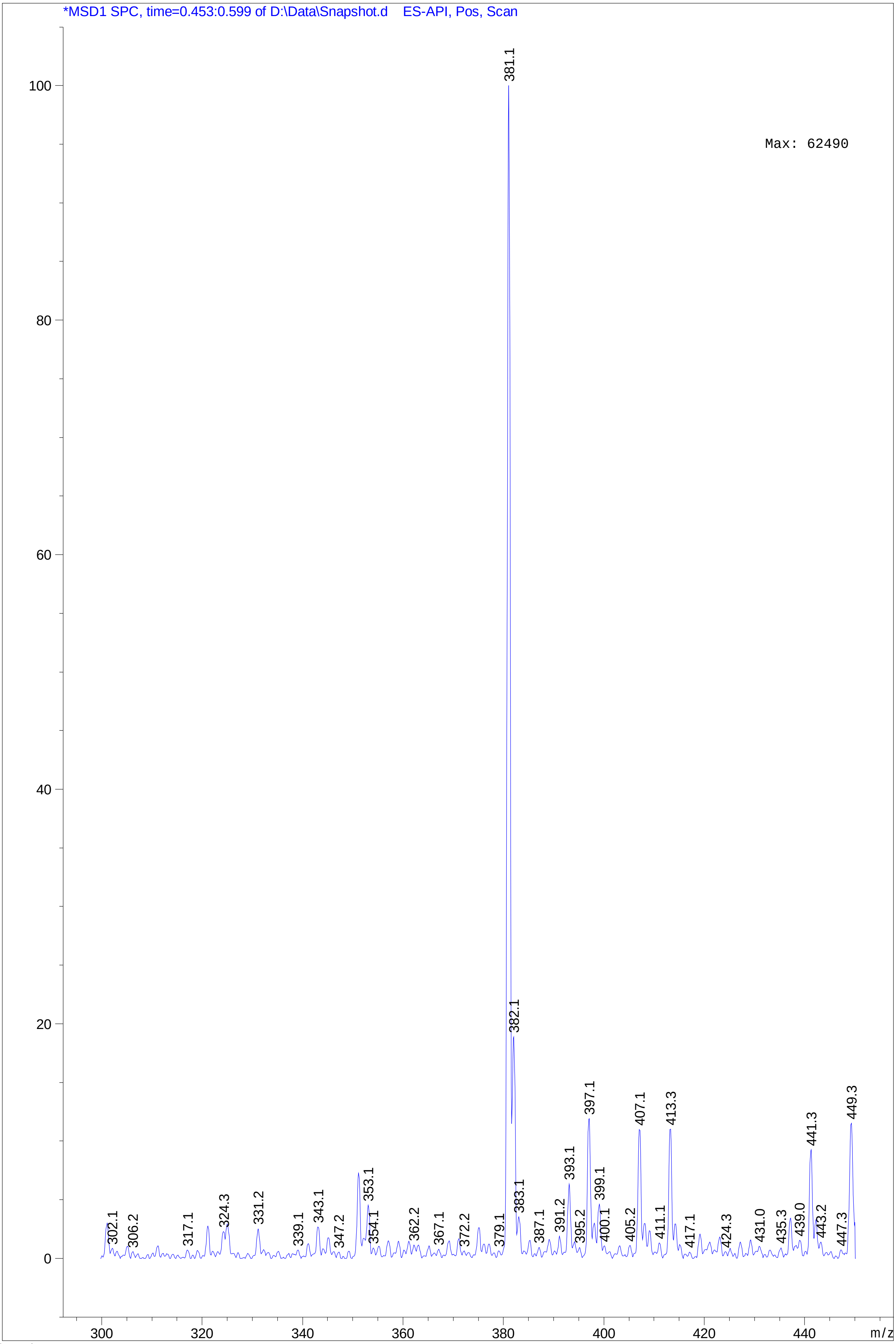

*MSD1 SPC, time=0.453:0.599 of D:\Data\Snapshot.d ES-API, Pos, Scan
Max: 62490
100
80
60
40
20
0
381.1
382.1
397.1
407.1
413.3
449.3
441.3
393.1
399.1
383.1
353.1
302.1
306.2
317.1
324.3
331.2
339.1
343.1
347.2
354.1
362.2
367.1
372.2
379.1
387.1
391.2
395.2
400.1
405.2
411.1
417.1
424.3
431.0
435.3
439.0
443.2
447.3
300
320
340
360
380
400
420
440
m/z
