# Supplementary material for: Oxa-Michael-initiated cascade reactions of levoglucosenone
Source: Beilstein J Org Chem. 2022 Oct 13;18:1457–62. doi: 10.3762/bjoc.18.151 (PMC9577383; doi:10.3762/bjoc.18.151)
Supplement: File 2 — 1H and 13C FID data and other spectra for new compounds. [file Beilstein_J_Org_Chem-18-1457-s002.zip › supporting information2/spectra files for new compounds - minimal/data for 6n/2/pdata/1/BWG0614C_2_1.pdf]

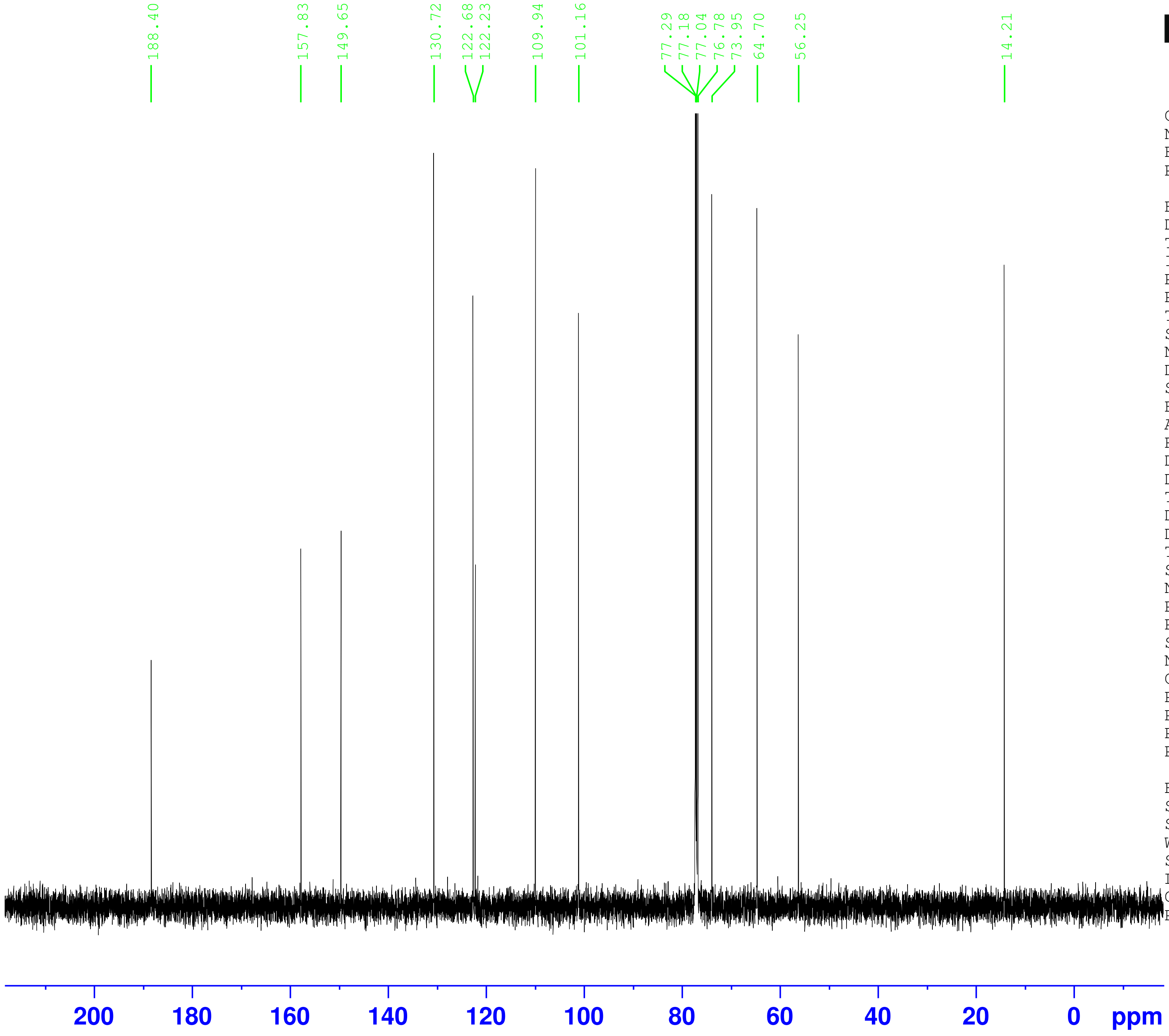

BRUKER

```
Current Data Parameters
NAME              BWG0614C
EXPNO                    2
PROCNO                   1

F2 - Acquisition Parameters
Date_             20201119
Time                 14.35 h
INSTRUM              spect
PROBHD   Z119470_0196 (
PULPROG            zgpg30
TD                   65536
SOLVENT              CDCl3
NS                     256
DS                       4
SWH              29761.904 Hz
FIDRES            0.908261 Hz
AQ               1.1010048 sec
RG                  191.36
DW                  16.800 usec
DE                    6.50 usec
TE                   298.0 K
D1              2.00000000 sec
D11             0.03000000 sec
TD0                      1
SFO1           125.7779086 MHz
NUC1                   13C
P1                   10.00 usec
PLW1           78.00000000 W
SFO2           500.1620006 MHz
NUC2                    1H
CPDPRG[2           waltz16
PCPD2                80.00 usec
PLW2           22.00000000 W
PLW12           0.35764000 W
PLW13           0.17989001 W

F2 - Processing parameters
SI                   32768
SF             125.7653320 MHz
WDW                     EM
SSB      0
LB                    1.00 Hz
GB       0
PC                    1.40
```
