# Supplementary material for: Oxa-Michael-initiated cascade reactions of levoglucosenone
Source: Beilstein J Org Chem. 2022 Oct 13;18:1457–62. doi: 10.3762/bjoc.18.151 (PMC9577383; doi:10.3762/bjoc.18.151)
Supplement: File 2 — 1H and 13C FID data and other spectra for new compounds. [file Beilstein_J_Org_Chem-18-1457-s002.zip › supporting information2/spectra files for new compounds - minimal/data for 7/NMR data for 7/1H/pdata/1/BWG0614B_1_1.pdf]

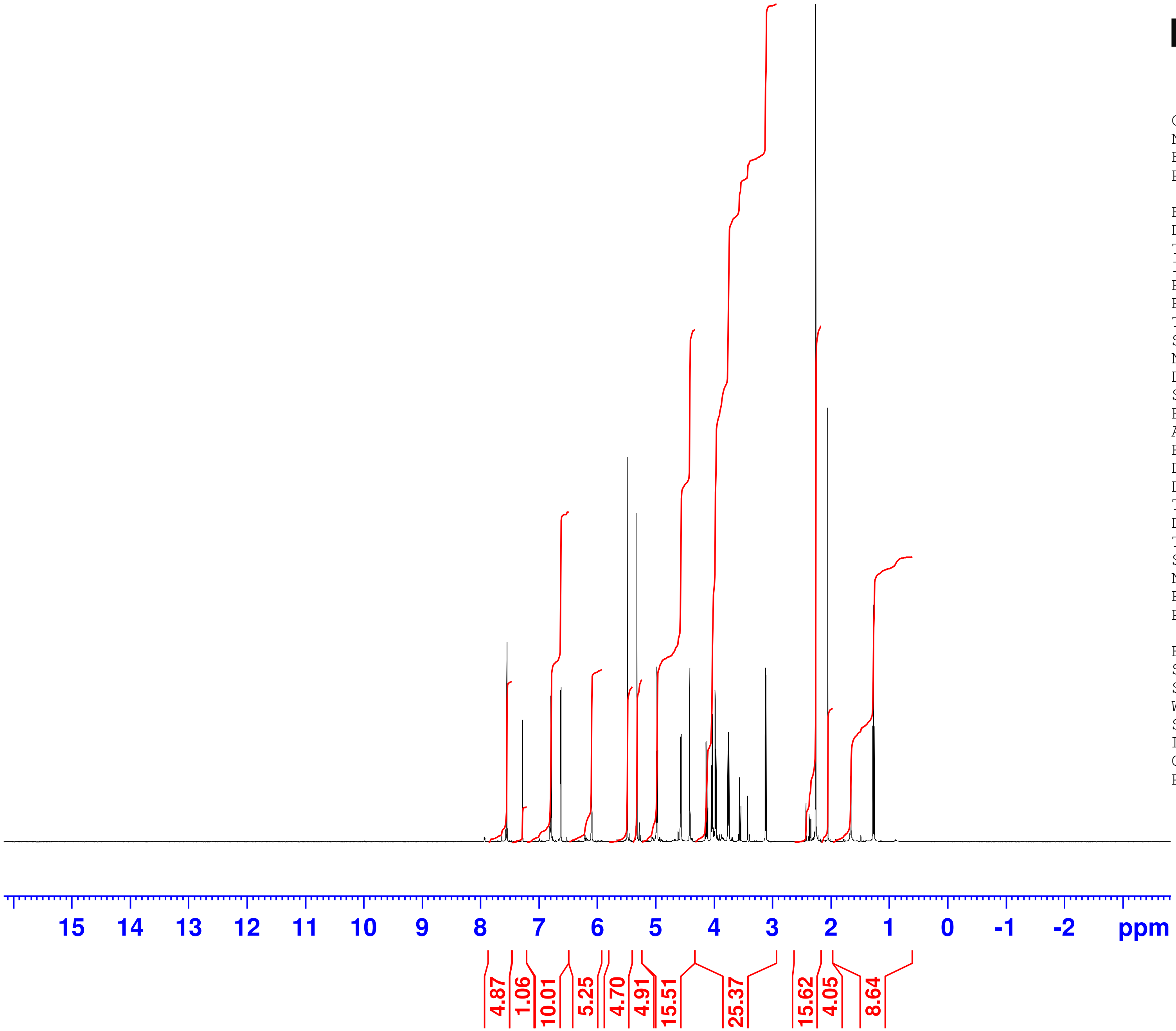


```
Current Data Parameters
NAME              BWG0614B
EXPNO                    1
PROCNO                   1

F2 - Acquisition Parameters
Date_             20201119
Time                 14.07 h
INSTRUM              spect
PROBHD    Z119470_0196 (
PULPROG               zg30
TD                   65536
SOLVENT              CDCl3
NS                      16
DS                       2
SWH              10000.000 Hz
FIDRES            0.305176 Hz
AQ               3.2767999 sec
RG                   108.2
DW                  50.000 usec
DE                    6.50 usec
TE                   298.0 K
D1              1.00000000 sec
TD0                      1
SFO1           500.1630885 MHz
NUC1                    1H
P1                   10.20 usec
PLW1           22.00000000 W

F2 - Processing parameters
SI                   65536
SF             500.1600000 MHz
WDW                     EM
SSB         0
LB                    0.30 Hz
GB          0
PC                    1.00
```
