# Supplementary material for: Oxa-Michael-initiated cascade reactions of levoglucosenone
Source: Beilstein J Org Chem. 2022 Oct 13;18:1457–62. doi: 10.3762/bjoc.18.151 (PMC9577383; doi:10.3762/bjoc.18.151)
Supplement: File 2 — 1H and 13C FID data and other spectra for new compounds. [file Beilstein_J_Org_Chem-18-1457-s002.zip › supporting information2/spectra files for new compounds - minimal/data for 7/NMR data for 7/cosy/pdata/1/email_BWG0614B_5_1.pdf]

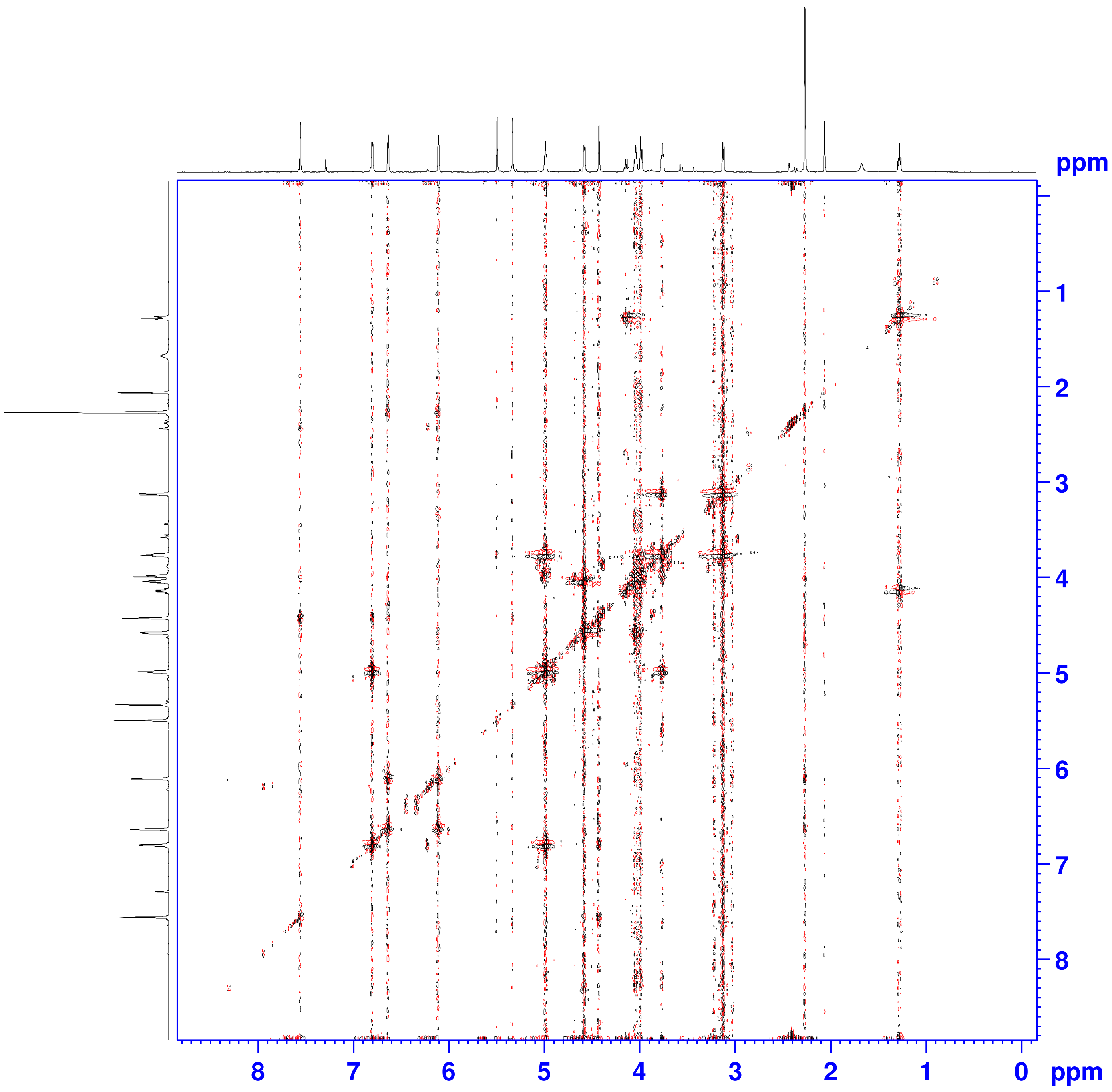

Current Data Parameters

NAME	BWG0614B
EXPNO	5
PROCNO	1

F2 - Acquisition Parameters

Date_	20201119
Time	15.23 h
INSTRUM	spect
PROBHD	Z119470_0196 (
PULPROG	cosygpmfphpp
TD	2048
SOLVENT	CDCl3
NS	4
DS	16
SWH	4504.504 Hz
FIDRES	4.398930 Hz
AQ	0.2273280 sec
RG	191.36
DW	111.000 usec
DE	6.50 usec
TE	298.0 K
D0	0.00009801 sec
D1	1.92995799 sec
D11	0.03000000 sec
D12	0.00002000 sec
D16	0.00020000 sec
IN0	0.00022200 sec
TDav	1
SFO1	500.1621725 MHz
NUC1	1H
P1	10.20 usec
P2	20.40 usec
P17	2500.00 usec
PLW1	22.00000000 W
PLW10	2.54320002 W
GPNAM[1]	SMSQ10.100
GPZ1	10.00 %
GPNAM[2]	SMSQ10.100
GPZ2	20.00 %
P16	1000.00 usec

F1 - Acquisition parameters

TD	256
SFO1	500.1622 MHz
FIDRES	35.191441 Hz
SW	9.006 ppm
FnMODE	States-TPPI

F2 - Processing parameters

SI	1024
SF	500.1600000 MHz
WDW	QSINE
SSB	2
LB	0 Hz
GB	0
PC	1.40

F1 - Processing parameters

SI	1024
MC2	States-TPPI
SF	500.1600000 MHz
WDW	QSINE
SSB	2
LB	0 Hz
GB	0
