# Supplementary material for: Oxa-Michael-initiated cascade reactions of levoglucosenone
Source: Beilstein J Org Chem. 2022 Oct 13;18:1457–62. doi: 10.3762/bjoc.18.151 (PMC9577383; doi:10.3762/bjoc.18.151)
Supplement: File 2 — 1H and 13C FID data and other spectra for new compounds. [file Beilstein_J_Org_Chem-18-1457-s002.zip › supporting information2/spectra files for new compounds - minimal/data for 7/NMR data for 7/DEPT/pdata/1/email_BWG0614B_3_1.pdf]

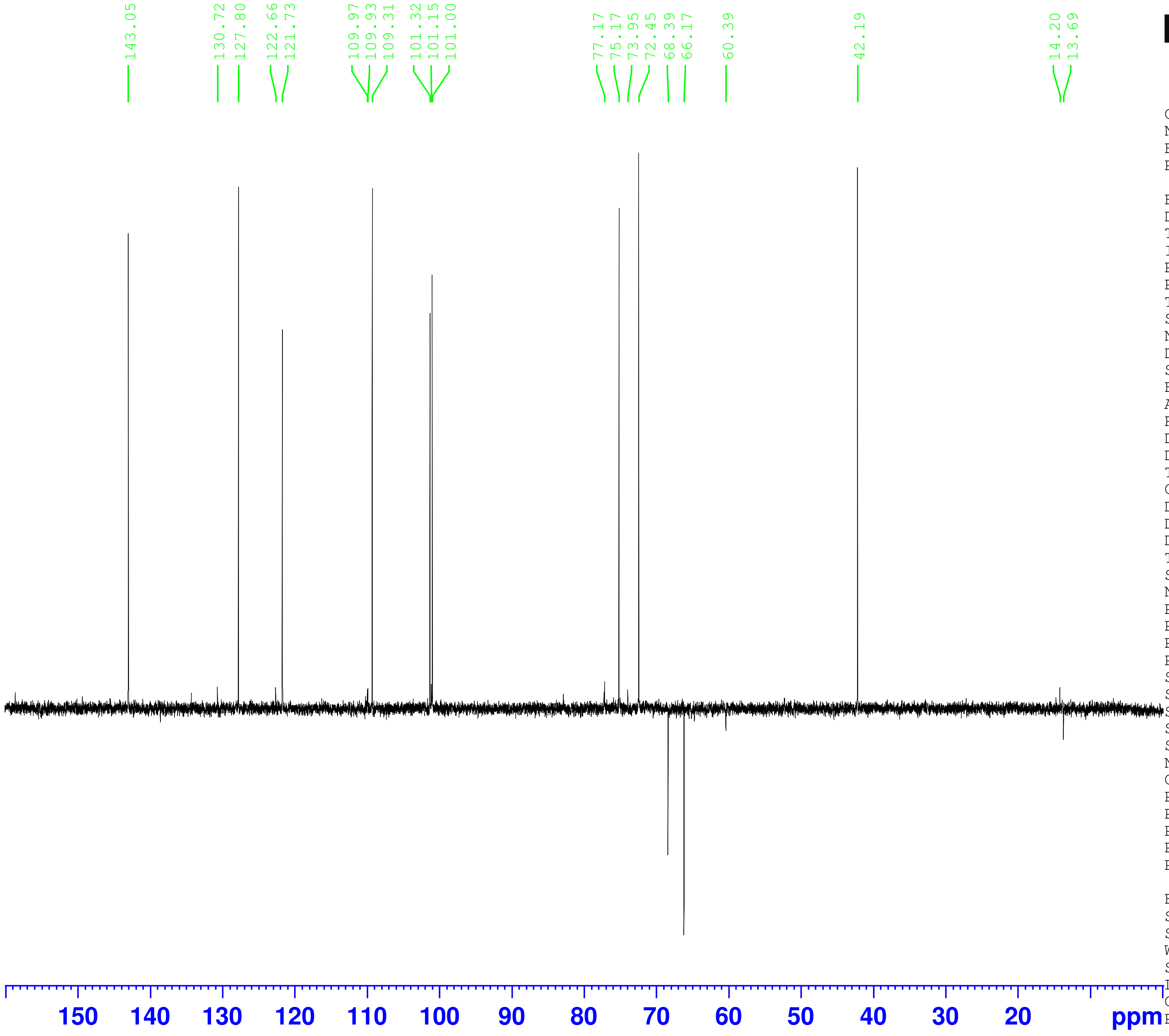

BRUKER

Current Data Parameters

NAME	BWG0614B
EXPNO	3
PROCNO	1

F2 - Acquisition Parameters

Date_	20201119
Time	15.19 h
INSTRUM	spect
PROBHD	Z119470_0196 (
PULPROG	deptsp135
TD	65536
SOLVENT	CDCl3
NS	256
DS	8
SWH	20161.291 Hz
FIDRES	0.615274 Hz
AQ	1.6252928 sec
RG	191.36
DW	24.800 usec
DE	6.50 usec
TE	298.0 K
CNST2	145.0000000
D1	2.00000000 sec
D2	0.00344828 sec
D12	0.00002000 sec
TD0	1
SFO1	125.7753933 MHz
NUC1	13C
P1	10.00 usec
P13	2000.00 usec
PLW0	0 W
PLW1	78.00000000 W
SPNAM[5]	Crp60comp.4
SPOAL5	0.500
SPOFFS5	0 Hz
SPW5	11.91800022 W
SFO2	500.1620006 MHz
NUC2	1H
CPDPRG[2	waltz16
P3	10.20 usec
P4	20.40 usec
PCPD2	80.00 usec
PLW2	22.00000000 W
PLW12	0.35764000 W

F2 - Processing parameters

SI	32768
SF	125.7653320 MHz
WDW	EM
SSB	0
LB	1.00 Hz
GB	0
PC	1.40
