# Supplementary material for: Oxa-Michael-initiated cascade reactions of levoglucosenone
Source: Beilstein J Org Chem. 2022 Oct 13;18:1457–62. doi: 10.3762/bjoc.18.151 (PMC9577383; doi:10.3762/bjoc.18.151)
Supplement: File 2 — 1H and 13C FID data and other spectra for new compounds. [file Beilstein_J_Org_Chem-18-1457-s002.zip › supporting information2/spectra files for new compounds - minimal/data for 7/NMR data for 7/HMBC/pdata/1/email_BWG0614B_6_1.pdf]

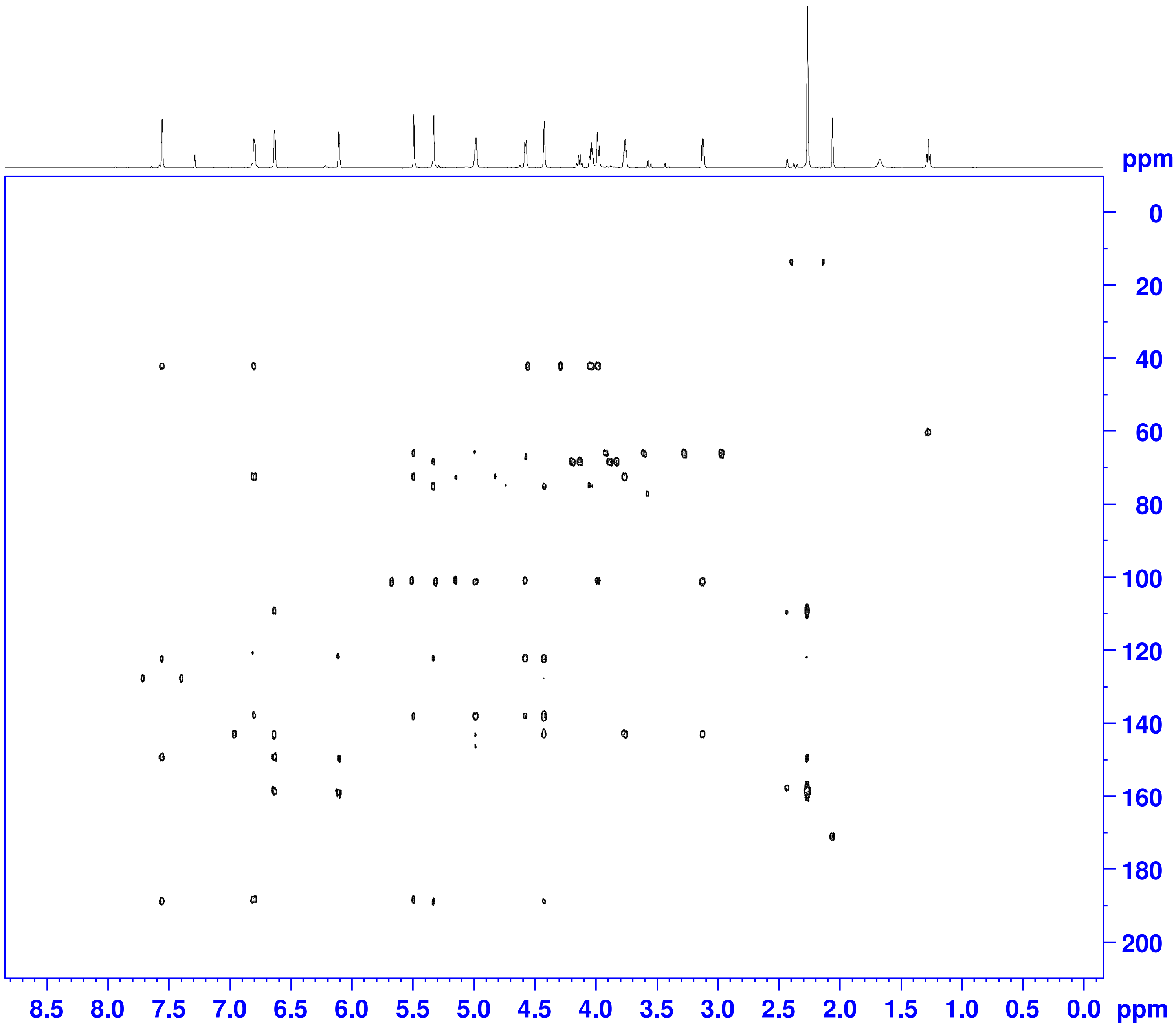

Current Data Parameters
NAME BWG0614B
EXPNO 6
PROCNO 1

F2 - Acquisition Parameters
Date_ 20201119
Time 16.02 h
INSTRUM spect
PROBHD Z119470_0196 (
PULPROG hmbcgpndqf
TD 4096
SOLVENT CDCl3
NS 8
DS 16
SWH 4504.504 Hz
FIDRES 2.199465 Hz
AQ 0.4546560 sec
RG 191.36
DW 111.000 usec
DE 6.50 usec
TE 298.0 K
CNST13 8.0000000
D0 0.00000300 sec
D1 1.35991704 sec
D6 0.06250000 sec
D16 0.00020000 sec
IN0 0.00001810 sec
TDav 1
SFO1 500.1621725 MHz
NUC1 1H
P1 10.20 usec
P2 20.40 usec
PLW1 22.00000000 W
SFO2 125.7779086 MHz
NUC2 13C
P3 10.00 usec
PLW2 78.00000000 W
GPNAM[1] SMSQ10.100
GPZ1 50.00 %
GPNAM[2] SMSQ10.100
GPZ2 30.00 %
GPNAM[3] SMSQ10.100
GPZ3 40.10 %
P16 1000.00 usec

F1 - Acquisition parameters
TD 128
SFO1 125.7779 MHz
FIDRES 431.629822 Hz
SW 219.628 ppm
FnMODE QF

F2 - Processing parameters
SI 4096
SF 500.1600000 MHz
WDW SINE
SSB 0
LB 0 Hz
GB 0
PC 1.40

F1 - Processing parameters
SI 1024
MC2 QF
SF 125.7653320 MHz
WDW SINE
SSB 0
LB 0 Hz
GB 0
